# UV-mediated hydrophosphinylation of unactivated alkenes with phosphinates under batch and flow conditions†

**DOI:** 10.1039/c7ra12977g

**Published:** 2018-02-23

**Authors:** Fabien Gelat, Maxime Roger, Christophe Penverne, Ahmed Mazzad, Christian Rolando, Laëtitia Chausset-Boissarie

**Affiliations:** USR 3290, MSAP, Miniaturisation pour la Synthèse l'Analyse et la Protéomique and FR 2638, Institut Eugène-Michel Chevreul, Université de Lille F-59000 Lille France Laetitia.chausset-boissarie@univ-lille1.fr Fabien.gelat@univ-lille1.fr

## Abstract

A UV-mediated hydrophosphinylation of unactivated alkenes with *H*-phosphinates and hypophosphorous acid under radical free conditions is presented. The reaction affords selectively a large number of structurally diverse organophosphorous compounds in moderate to good yields under mild reaction conditions in the presence of an organic sensitizer as catalyst irradiated by UV-A LEDs. Furthermore, the high yielding hydrophosphinylation in continuous flow is disclosed.

## Abstract 2

In this study, we report an efficient UV-mediated hydrophosphinylation of unactivated alkenes with *H*-phosphinates and hypophosphorous acid under radical free conditions in batch and in continuous flow.

## Graphical abstract

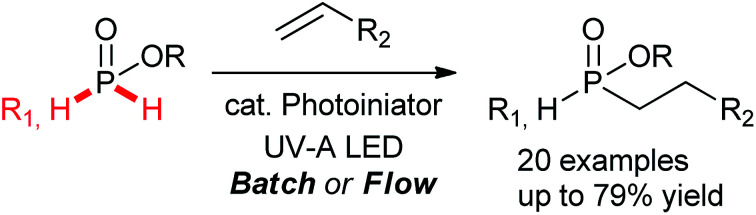

## Introduction

**Scheme 1:**
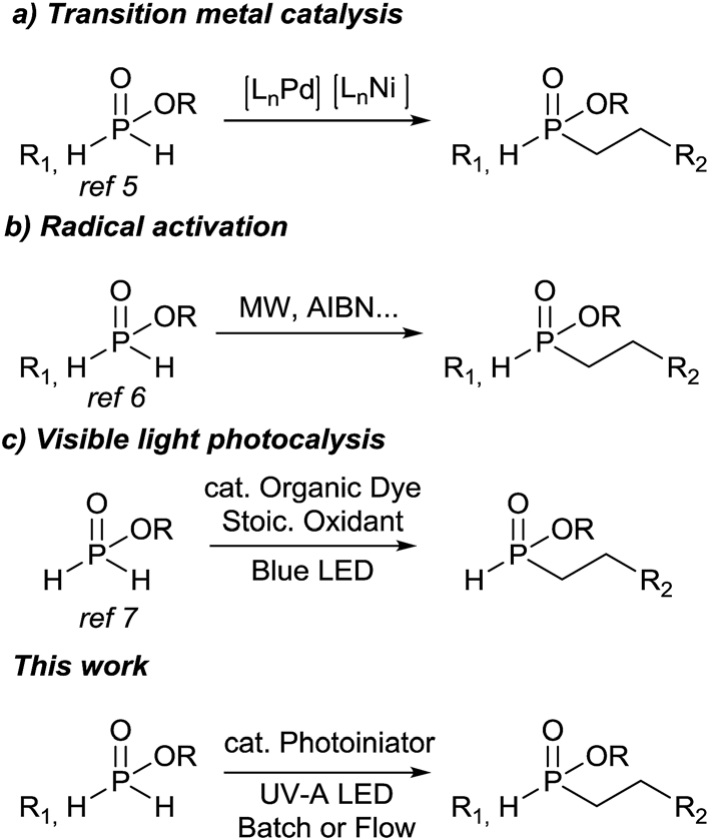
Hydrophosphinylation of phosphinates with unactivated alkenes.

Organophosphorus compounds have attracted much attention due to their wide range of applications in materials, catalysis, natural bioactive products and pharmaceuticals.^1^ In particular, phosphinates (P(O)(OR)R^1^R^2^, R^1^/R^2^ = hydrogen and/or carbon) have been the target of numerous synthetic efforts as they are versatile precursors to organophosphorus compounds and represent a sustainable alternative to the use of phosphorous trichloride.^2^ Indeed, phosphinates can be considered as the synthetic equivalents of phosphorous trichloride with several advantages in terms of stability and toxicity. In this regard, the development of efficient and selective methodologies for the preparation and functionalization of phosphinates (P(O)(OR)H_2_) and *H*-phosphinates (P(O)(OR)R^1^H) has been extensively studied, mostly by the group of Montchamp.^2,3^ Hydrophosphinylation of unactivated alkenes is one of these routes that provide a direct access to functionalized organophosphorous compounds.^4^ This atom economical process has been reported to proceed *via* transition metal catalysis,^5^ or radical activation (Scheme 1a and b).^6^ Despite the significant progress in this chemistry, these transformations suffer from some drawbacks like the relatively harsh conditions, narrow substrates scopes and costly catalytic system thus limiting their chemical and pharmaceutical applications. Recently, Lakhdar and co-workers reported a hydrophosphinylation of unactivated alkenes with ethyl and butyl phosphinates under photocatalytic conditions (Scheme 1c),^7^ even though diphenyl iodonium triflate which acted as sacrificial oxidant is not a heavy metal, the use of catalytic quantity is always preferred in term of sustainability and environmental benignity. Alternatively, pioneering work from the group of Dondoni^8^ demonstrated that P(O)-centered radical can be generated from *H*-phosphonates under UV-A irradiation, in the presence of 2,2-dimethoxy-2-phenylacetophenone (DMPA) as photoinitiator, and added to alkene functionalized carbohydrates. However, the reaction requires the use of a large excess of phosphonates (100 eq.). Mathé and coworkers^9^ then extended this free radical hydrophosphonylation to activated and unactivated alkenes, similar reaction with hypophosphorous acid and *H*-phosphinates derivatives^10^ have not been studied previously.

In this context, we envisaged that continuous-flow systems in combination with a sensitizer irradiated by UV-A LEDs (*λ* = 365 nm) would result in a significant enhancement of the reaction. Over the last decade, interest has grown for flow chemistry based on microfluidic technology in particular towards large-scale application due to its significant improvement over traditional batch reactors concerning reduced consumption of chemicals, solvents and time together with enhanced yields, selectivity and control over reaction conditions. An easy scale up is also one of the characteristic of photo-reactions which are conducted in microreactors. In batch reactors, light penetration through the reaction media is limited which restrains the efficiency of photochemical processes. This drawback can be overcome with continuous microflow reactors since their small optical lengths improve sample irradiation and also enhance heat and mass transfer.^11^ Therefore, in view of the growing demand to develop efficient, mild and sustainable methods to access phosphinates and as a continuation of our interest in photocatalyzed processes,^12^ we herein report an original UV-mediated hydrophosphinylation of unactivated alkenes with hypophosphorous acid and less reactive *H*-phosphinates derivatives under batch and flow conditions.

## Results and discussion

### Hydrophosphinylation of unactivated alkenes with *H*-phosphinates under batch conditions

Initially, the hydrophosphinylation of relatively challenging *H*-phosphinate (1a) with octene (2a) in equimolar amounts in the presence of a photoinitiator under UV-A irradiation was selected as a model to investigate the reaction. The use of one equivalent of 4,4′-dimethoxybenzophenone (4,4′-DMBP) was found to be effective furnishing 3a after stirring in acetonitrile under an ambient inert atmosphere for 3 h (Table 1, entry 1, 42%). An investigation of solvents showed that the reaction efficiency was further enhanced when performed in DMSO (Table 1, entry 4, 69%) or acetic acid^6*b*,13^ (entry 5, 70%) as previously observed for radical process with *H*-phosphinates, whereas moderate yields were obtained with ethyl acetate (entry 2, 46%) or DMF (entry 3, 52%). Importantly a very low catalyst loading could promote the reaction yield up to 84% (entries 6 and 7). Moreover, the loading could be further decreased but a prolonged reaction time was required (Table 1, entry 8, 86%). Interestingly, the photoinitiator DMPA (2,2-dimethoxy-2-phenylacetophenone) was not efficient in our case since the phosphorylated product was formed in a moderate yield (Table 1, entry 15, 32%). Furthermore, none of the common photoinitiators (thioxantone, 4-methoxyacetophenone, benzophenone) exhibited a better catalytic effect (Table 1, entries 12–14). Control experiments revealed that the photoinitiator, inert conditions and light were essential for this reaction (Table 1, entries 9–11). It is noteworthy that using a 4,4′-DMBP derivative functionalized with an ionic liquid moiety^14^ (4,4′-(2-(1-methylimidazolium)ethoxy)benzophenone dibromide, DIBP) as photosensitizer led to the same conversions as previously observed furnishing an excellent way to simplify the purification procedure by simple washing of the reaction media thus improving the synthetic efficiency by avoiding chromatography (Table 1, entry 16).

**Table 1:** Optimization of the reaction conditions^a^

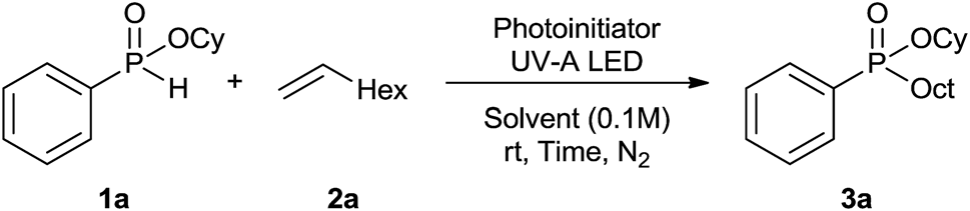					
Entry	Photoinitiator	Equivalent	Solvent	Time (h)	Yield^b^ (%)
1	4,4′-DMBP^c^	1	MeCN	3	42
2	4,4′-DMBP	1	EtOAc	3	46
3	4,4′-DMBP	1	DMF	3	52
4	4,4′-DMBP	1	DMSO	3	69
5	4,4′-DMBP	1	AcOH	3	70
6	4,4′-DMBP	0.5	DMSO	3	72
7	4,4′-DMBP	0.1	DMSO	5	84
8	4,4′-DMBP	0.05	DMSO	17	86
9	—	—	DMSO	5	10
10^d^	4,4′-DMBP	0.1	DMSO	5	—
11^e^	4,4′-DMBP	0.1	DMSO	5	5
12	Thioxantone	0.1	DMSO	5	76
13	4-MAP^f^	0.1	DMSO	5	19
14	Benzophenone	0.1	DMSO	5	77
15	DMPA^g^	0.1	DMSO	5	32
16	DIBP^h^	0.1	DMSO	16	79 (76)^i^

a Reaction condition: 1a (0.3 mmol, 1 equiv.), 2a (0.3 mmol, 1 equiv.), photoinitiator (*x* equiv.), solvent ([0.1 M]), under nitrogen and UV-A LED irradiation (*λ* = 365 ± 15 nm, 230 mW cm^−2^) at room temperature.

b Derived from ^31^P crude NMR spectra on integration of all formed species.

c 4,4′-DMBP = 4,4′-dimethoxybenzophenone.

d Without irradiation.

e Under ambient atmosphere.

f 4-MAP = 4-methoxyacetophenone.

g DMPA = 2,2-dimethoxy-2-phenylacetophenone.

h DIBP = 4,4′-(2-(1-methylimidazolium)ethoxy)benzophenone dibromide.

i The isolated yield is shown in parentheses.

The generality of the method was investigated, with the scope of the reaction being explored with respect to the *H*-phosphinate component, and the results are summarized in Table 2. Various P(O)–H compounds were employed to react with 1-octene 2a giving the corresponding 3a–g with isolated yields ranging from 30 to 76% (Table 2, entries 1–7). As expected, in all of the cases the anti Markovnikov product was observed. The reaction is not limited to *H*-phosphinate esters as *H*-phosphinate acid 1b could also deliver the desired product with a good yield (entry 2, 64%) in contrast to *H*-phosphinate acid 1c whose corresponding phosphinate acid product 3c was difficult to obtain in high purity (entry 3, 30%). However, *H*-phosphinate including unactivated alkyl and benzyl derivatives reacted successfully regardless of the ester chosen (Table 2, entries 4–6). When using (hydroxymethyl)-*H*-phosphinate ester, which was reported to have several application,^15^ the branched product 3g was obtained with a significant yield of 75% (Table 2, entry 7). To further evaluate the substrate scope, a series of alkenes were tested with *H*-phosphinate 1a (Table 2, entries 8–12). The reaction with allyl benzene gave the corresponding product 3h with 48% of yield (Table 2, entry 8). Interestingly, 1a reacted with hindered alkene giving 3i with a modest yield of 27% (Table 2, entry 9). Note that halogens like Br and alcohol groups were well tolerated which indicates a potential for further functionalization (Table 2, entries 10–12).

**Table 2:** Hydrophosphinylation of alkenes with *H*-phosphinates^a^1

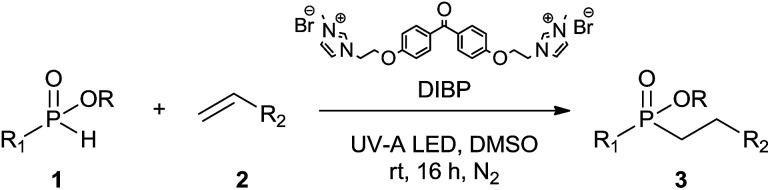				
Entry	*H*-Phosphinate 1	Alkene 2	Product 3	Yield (%)
1	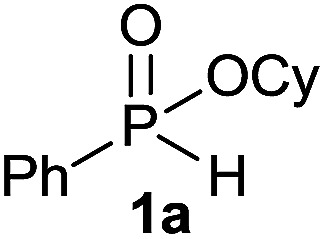	1-Octene 2a	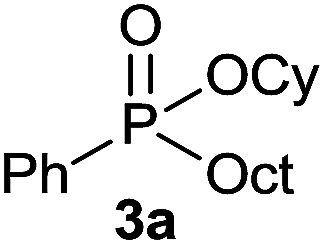	76
2	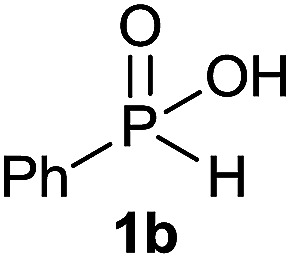	1-Octene 2a	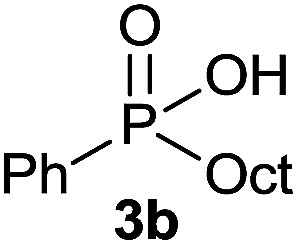	64
3	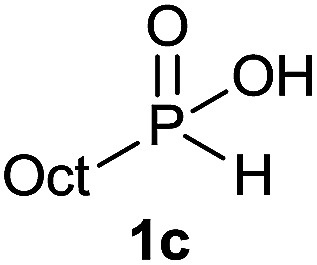	1-Octene 2a	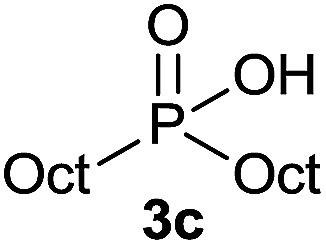	30^b^
4	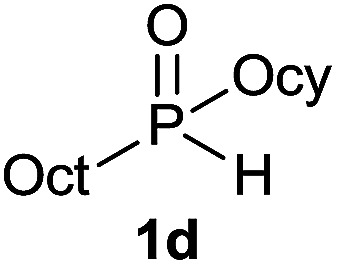	1-Octene 2a	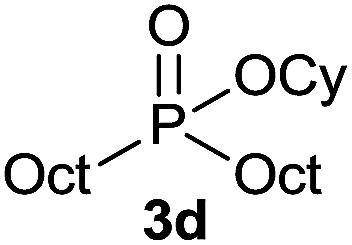	73
5	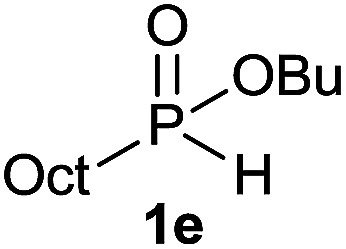	1-Octene 2a	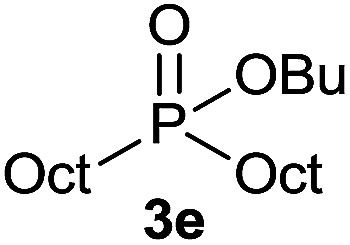	71
6^c^	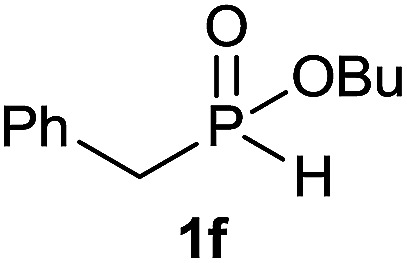	1-Octene 2a	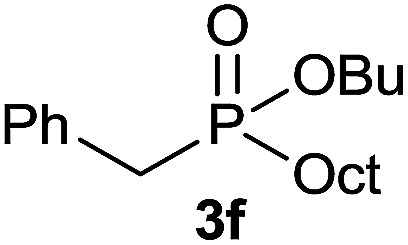	51
7	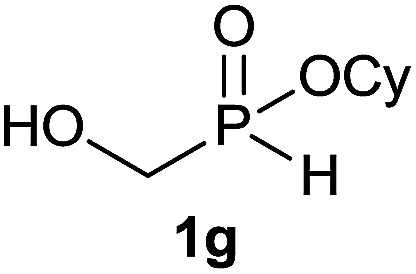	1-Octene 2a	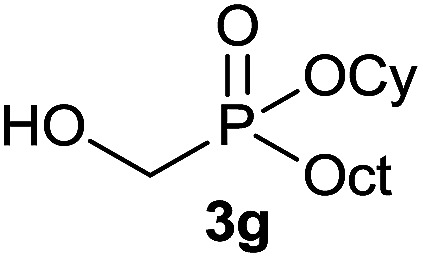	75
8	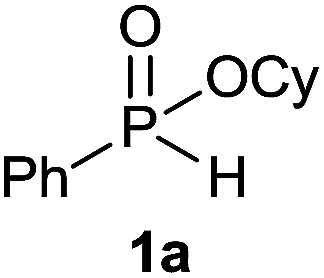	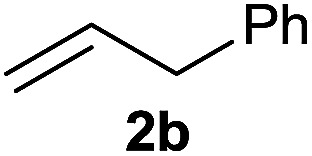	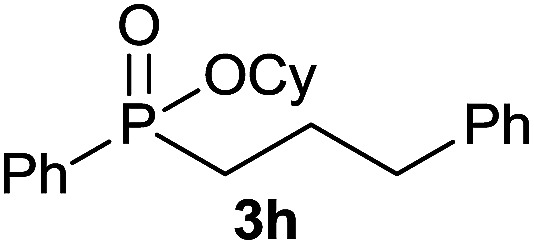	48
9^c^	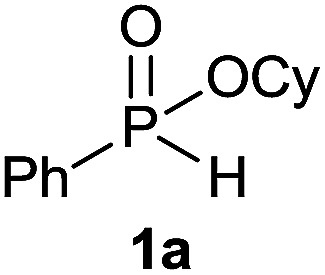	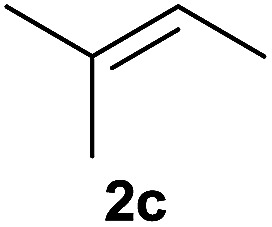	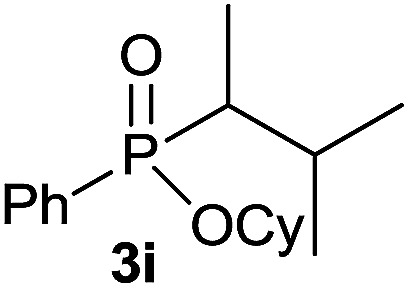	27
10	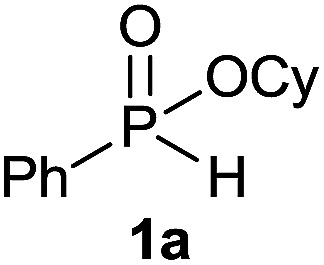	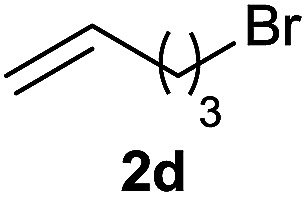	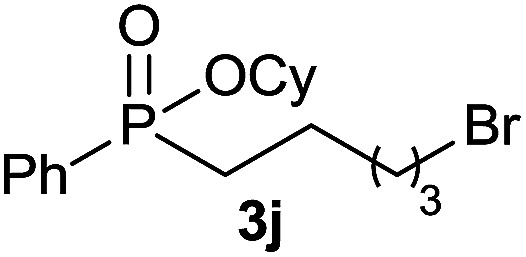	38
11^c^	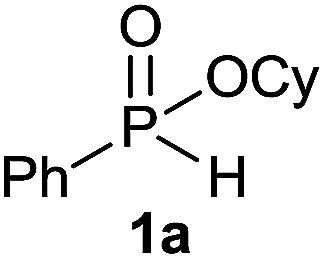	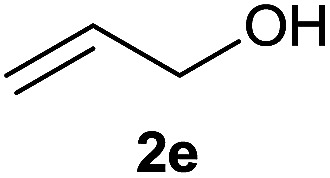	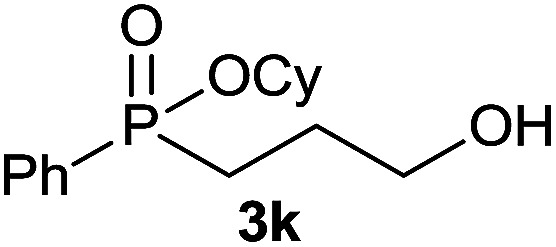	56
12^c^	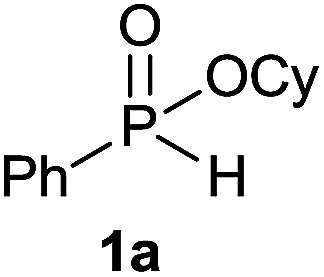	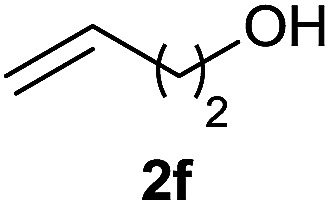	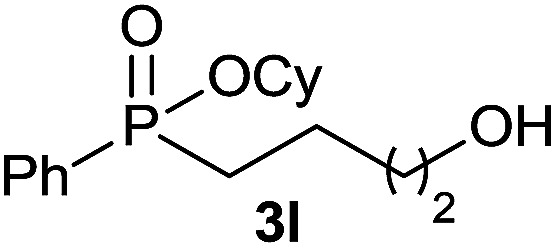	69

a Reaction condition: all reactions unless specified were carried out using 1 (0.3 mmol, 1 equiv.), 2 (0.3 mmol, 1 eq.), DIBP (0.1 equiv.) in DMSO ([0.1 M]) under nitrogen and UV-A LED irradiation (*λ* = 365 ± 15 nm, 230 mW cm^−2^) at room temperature for 16 h, isolated yield.

b Derived from ^31^P crude NMR spectra on integration of all formed species.

c (0.5 equiv.) of DIBP.

**Scheme 2:**
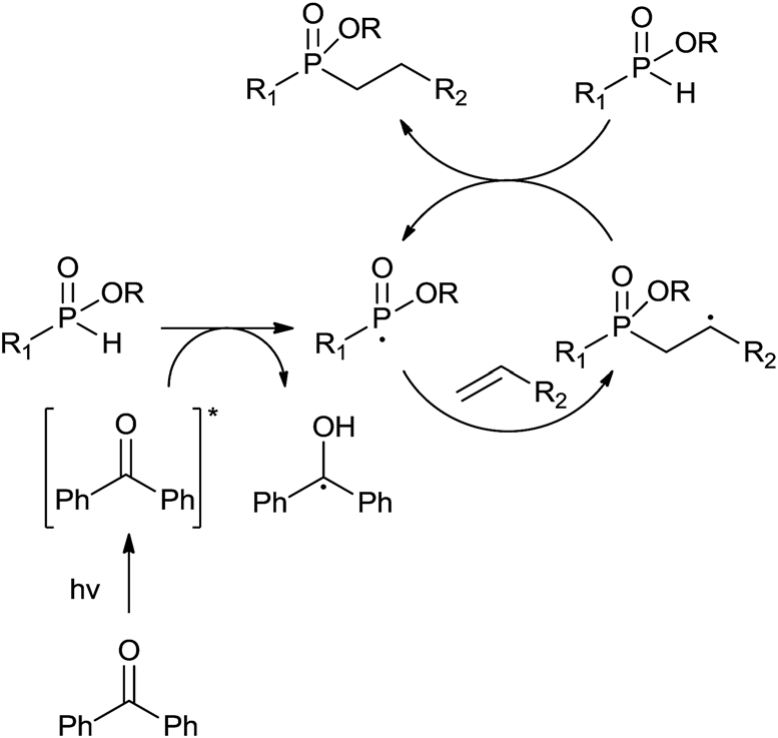
Proposed reaction mechanism for the photoinduced hydrophosphinylation of unactivated alkenes with *H*-phosphinates.

Although the detailed reaction mechanism is unclear, based on literature precedents^6*i*,16^ all these results could be well explained by a radical chain mechanism as depicted in Scheme 2. Upon irradiation, the excited state of the photoinitiator^17^ abstracts a proton from *H*-phosphinates to form the phosphoryl radical^18^ which adds to the terminal carbon of the alkenes. The carbon centered radical can then abstract a proton from *H*-phosphinate 1 to regenerate the phosphoryl radical.

### Hydrophosphinylation of unactivated alkenes with *H*-phosphinates under flow conditions

With the successful results for the hydrophosphinylation under batch conditions, the reaction was then performed under continuous flow to further improve its efficiency in a shorter amount of time. The continuous flow hydrophosphinylation was performed using a continuous flow microfluidic system composed of a high pressure syringe pump delivering the homogeneous reaction mixture at specific flow rates to a commercially available microreactor (Mikroglas Dwell Device® microreactor from Invenios Europe, Langen, Germany) irradiated by HP UV-A lamp. This microreactor is made up of Foturan® glass that is transparent up to 300 nm allowing to work at a wide range of wavelengths (UV-A & visible).

**Scheme 3:**
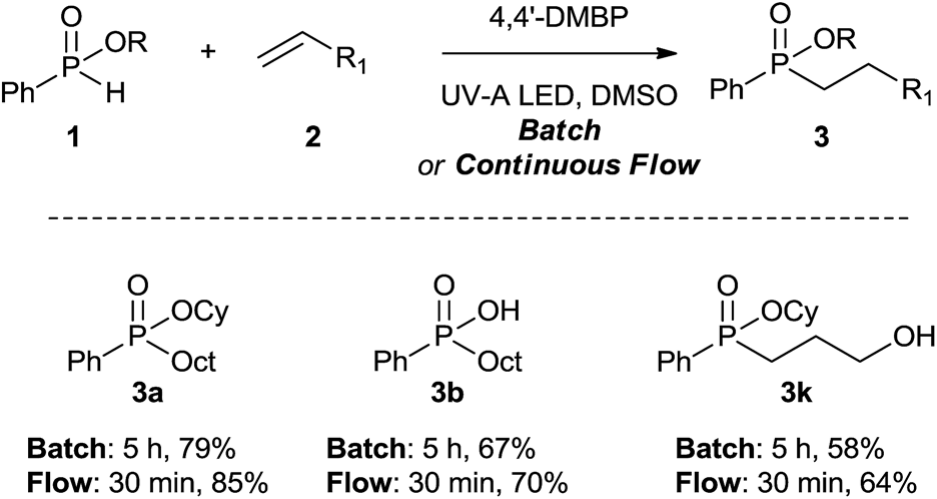
Hydrophosphinylation of alkenes with *H*-phosphinates 1 under batch and flow conditions. (a) Reactions conditions in batch: 1 (0.3 mmol, 1 equiv.), 2 (0.3 mmol, 1 eq.), 4,4′-DMPB (0.1 equiv.) in DMSO ([0.1 M]) under nitrogen and UV-A LED irradiation (*λ* = 365 ± 15 nm, 230 mW cm^−2^) at room temperature, 5 h, isolated yield. (b) Reaction conditions in continuous flow: 1 (0.3 mmol, 1 equiv.), 2 (0.3 mmol, 1 equiv.), 4,4′-DMPB (0.1 equiv.) in DMSO ([0.1 M]) under nitrogen and UV-A LED irradiation (*λ* = 365 ± 15 nm, 230 mW cm^−2^) with Dwell device manufactured by Mikroglass Chemtech Mainz, Germany with a rectangular shape of dimensions 115 mm × 2 mm × 0.5 mm as photo-microreactor, at room temperature for 30 min of residence time, isolated yield.

We were delighted to find that *H*-phoshinate derivatives (3a, b, k) could be obtained with 4,4′-DMBP as photoinitiator within a shorter reaction time than in batch (30 min *vs.* 5 h), indicating the importance of the short light path length provided by the microreactor, with slightly higher isolated yield, highlighting the potentials of this process (Scheme 3). This results offer the possibility to conduct the reaction on large scale without erosion of the yields.

### Hydrophosphinylation of unactivated alkenes with hypophosphorous acid under batch conditions

**Scheme 4:**
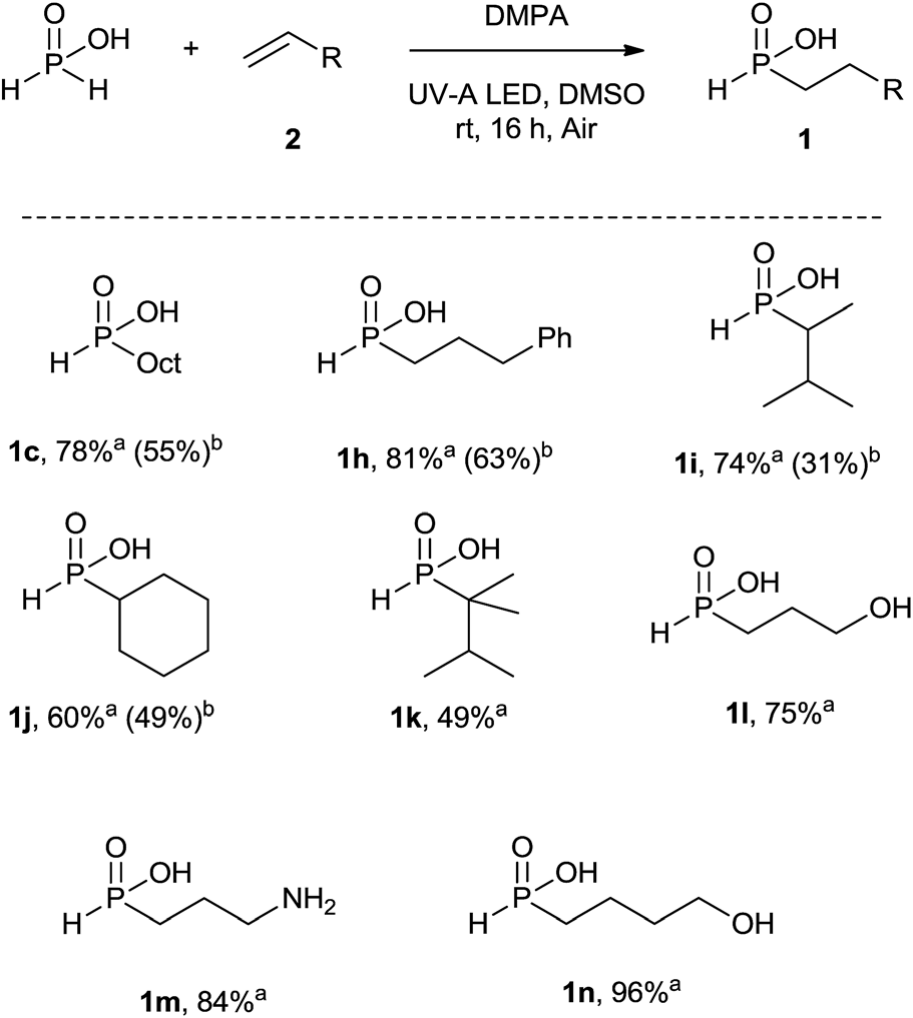
Hydrophosphinylation of alkenes with hypophosphorous acid. All reactions (unless otherwise specified) were carried out using H_3_PO_2_ (0.3 mmol, 2 equiv.), 2 (0.15 mmol, 1 equiv.), DMPA (0.2 equiv.) in DMSO ([0.1 M]) under UV-A LED irradiation (*λ* = 365 ± 15 nm, 230 mW cm^−2^) at room temperature for 16 h. ^a^Derived from ^31^P crude NMR spectra on integration of all formed species. ^b^Isolated yield.

To further demonstrate the utility of our protocol, we sought to test its potential for the hydrophosphinylation of various alkenes with hypophosphorous acid to form the previous precursor *H*-phosphinate acid (Scheme 4).

A slight modification of the previous batch conditions (see Table 1, ESI†) allowed us to obtain the corresponding *H*-phosphinates 1 with ^31^P NMR yield ranging from 49 to 96%. A 2 : 1 molar ratio of hypophosphorous acid–alkenes was necessary to obtain a high conversion without the formation of the disubstituted by-product. However despite the observed high conversions, isolated yields were modest due to difficult purifications. In all of the cases, the reaction was chemoselective where only mono substituted phosphinates were observed. As for *H*-phosphinate, the reaction proceeded well with terminal (1c–h) and cyclic alkenes (1j). Hindered alkenes delivered the desired compounds like 1i with a moderate yield of 31%, which is still higher than that obtained with *H*-phosphinate ester. *H*-Phosphinate acid 1k however was difficult to obtain in high purity. Similar to the first reaction assessed, the reaction tolerates functional groups such as amines or alcohols (1l–n) but the obtained *H*-phosphinate acids were not isolated. Although the scope of this reaction seems to be limited, *H*-phosphinate acids could be easily esterified^19^*in situ* and therefore the resulting procedure offers significant synthetic advantages.

## Experimental section

### General information

All reagents were purchased from commercial suppliers (Strem Chemicals Inc., Sigma-Aldrich or Alfa Aesar) and were used without further purification. Thin-layer chromatography (TLC) was performed on Silica gel 60 F254 plates (Merck) and visualized under UV (254 nm) or by staining with potassium permanganate or phosphomolybdic acid. Column chromatography was performed with 63–200 mesh silica gel. NMR spectra were recorded on a Bruker AVANCE 300 spectrometer at 300 MHz (75 MHz). Chemical shifts are reported in parts per million relative to solvent signal and coupling constants are reported in hertz (Hz). High-resolution mass spectra (HRMS) were performed on a Thermo LTQ Orbitrap mass spectrometer using nanoESI ionization. *H*-Phosphinates derivatives 1 and 4,4′-(2-(1-methylimidazolium)ethoxy)benzophenone dibromide (DIBP)^14^ were prepared according to the reported procedure. The illumination was performed by UV-A LEDs (365 nm, irradiance = 230 mW cm^−2^) Omnicure® AC475 model from Lumen Dynamics (Excelitas Technologies, Waltham, MA, USA). Note that the irradiance was measured at the surface of the reactor using a radiometer.

### General procedure A for the hydrophosphinylation with *H*-phosphinate 1 derivatives under batch conditions

A solution of a selected *H*-phosphinate 1 (0.3 mmol, 1 equiv.), an alkene 2 (0.3 mmol, 1 equiv.) and DIPB (0.03–0.15 mmol, 10–50 mol%) in degazed DMSO (1.5 mL) was irradiated under 365 nm and N_2_ for 16 h. Ethyl acetate (30 mL) was added and the reaction mixture was washed with saturated solution of NaHCO_3_ (2 × 15 mL) and brine (2 × 15 mL). The organic layer was dried over MgSO_4_, filtered and concentrated under reduce pressure. The residue was then purified by flash chromatography on silica gel if necessary to afford the corresponding organophosphorous compound.

### General procedure B for the hydrophosphinylation with hypophosphorous acid

To a 50% aqueous solution of hypophosphorous acid (79.2 mg, 0.6 mmol, 2 equiv.) in DMSO (3 mL) was added an alkene 2 (0.3 mmol, 1 equiv.), 4,4′-DMPA (15.5 mg, 0.06 mmol, 20 mol%). The reaction mixture was irradiated under 365 nm and ambient atmosphere for 16 h. Ethyl acetate (30 mL) was added and the organic layer was washed with brine (3 × 15 mL). To the organic layer a 0.03 M solution of NaHCO_3_ (30 mL, 1 mmol) was added. The aqueous layer was washed with ethyl acetate (2 × 15 mL), acidified with 1 M HCl (2 mL, 2 mmol), saturated with NaCl and extracted with ethyl acetate (30 mL). The last ethyl acetate layer was dried over Na_2_SO_4_, filtered and concentrated under reduce pressure. The residue was then purified by flash chromatography on silica gel using 9 : 1 : 0.5 DCM–MeOH–AcOH as the eluant to afford the desired pure *H*-phosphinate acid 1.

### Hydrophosphinylation of cyclohexyl phenyl-*H*-phosphinate 1a under flow condition

A solution of a cyclohexyl phenyl-*H*-phosphinate^20^1a (67.2 mg, 0.3 mmol, 1 equiv.), 1-octene 2a (33.6 mg, 0.3 mmol, 1 equiv.) and 10 mol% of 4,4′-dimethoxybenzophenone (7.2 mg) in degazed DMSO (1.5 mL) was pumped through the Mikroglas Dwell Device reactor (*V*_int_ = 1.15 mL) at 38.33 μL min^−1^, residence time of 30 min and irradiated by UV-A LEDs. Ethyl acetate (30 mL) was added to the collected reaction mixture and the reaction mixture was washed with saturated solution of NaHCO_3_ (2 × 15 mL) and brine (2 × 15 mL). To the organic layer was dried over MgSO_4_, filtered and the solvent was removed under reduce pressure to afford 3a (85.6 mg, 0.25 mmol, 85%) as a colorless oil.

#### Cyclohexyl octyl(phenyl)phosphinate (3a)

Following the general procedure A, using cyclohexyl phenyl-*H*-phosphinate 1a (67.2 mg, 0.3 mmol, 1 equiv.), 1-octene (33.6 mg, 0.3 mmol, 1 equiv.) and 10 mol% of catalyst (17.8 mg) affords 3a (76.6 mg, 0.23 mmol, 76%) as a colorless oil. ^31^P NMR (121 MHz, CDCl_3_): *δ* = 43.5 (s); ^1^H NMR (300 MHz, CDCl_3_): *δ* = 7.81–7.72 (m, 2H), 7.55–7.39 (m, 3H), 4.30–4.16 (m, 1H), 2.06–2.11 (m, 24H), 0.83 (t, *J* = 6.8 Hz, 3H); ^13^C NMR (75 MHz, CDCl_3_): *δ* = 132.3 (d, *J*_PC_ = 122 Hz), 132.0 (d, *J*_PCCCC_ = 2.7 Hz), 131.7 (d, *J*_PCCC_ = 9.7 Hz, 2C), 128.5 (d, *J*_PCC_ = 12.2 Hz, 2C), 74.3 (d, *J*_POC_ = 6.8 Hz), 34.4 (d, *J*_POCC_ = 2.9 Hz), 33.8 (d, *J*_POCC_ = 4.3 Hz), 31.9, 31.0 (d, *J*_PCC_ = 16.0 Hz), 30.4 (d, *J*_PC_ = 101 Hz), 29.1 (2C), 25.3, 23.8, 23.8, 22.7, 21.8 (d, *J*_PCCC_ = 3.7 Hz), 14.2. HRMS (ESI) *m*/*z* calcd for C_20_H_34_O_2_P ([M + H]^+^) 337.2290, found 337.2289.

#### Octyl phenylphosphinic acid (3b)

A solution of phenyl phosphinic acid 1b (47.4 mg, 0.3 mmol, 1 equiv.), 1-octene 2a (33.6 mg, 0.3 mmol, 1 equiv.) and DIPB (6.7 mg, 0.03 mmol, 10 mol%) in degazed DMSO (3 mL) under N_2_ was irradiated under 365 nm for 15 h. Ethyl acetate (30 mL) was added and the reaction mixture was washed with brine (3 × 15 mL). To the organic layer was added a 0.03 M solution of sodium bicarbonate (30 mL, 1 mmol). The aqueous layer was washed with ethyl acetate (2 × 15 mL), acidified with 1 M HCl (2 mL, 2 mmol) and extracted with ethyl acetate (30 mL). The last ethyl acetate layer was dried over Na_2_SO_4_, filtered and the solvent was removed under reduce pressure to afford the desired pure compound 3b (51.8 mg, 0.19 mmol, 64%). ^31^P NMR (121 MHz, CDCl_3_): *δ* = 45.8 (s); ^1^H NMR (300 MHz, CDCl_3_): *δ* = 11.65 (br s, 1H), 7.72–7.58 (m, 2H), 7.45–7.24 (m, 3H), 1.83–1.64 (m, 2H), 1.46–1.26 (m, 2H), 1.24–1.03 (m, 10H), 0.78 (t, *J* = 6.7 Hz, 3H); ^13^C NMR (75 MHz, CDCl_3_): *δ* = 132.5 (d, *J*_PC_ = 127 Hz), 131.9, 131.2 (d, *J*_PCCC_ = 9.4 Hz, 2C), 128.4 (d, *J*_PCC_ = 11.4 Hz, 2C), 31.9, 30.8 (d, *J*_PCC_ = 16.3 Hz), 30.6 (d, *J*_PC_ = 98.7 Hz), 29.2 (2C), 22.7, 21.8 (d, *J*_PCCC_ = 2.6 Hz), 14.2; HRMS (ESI) *m*/*z* calcd for C_14_H_24_O_2_P ([M + H]^+^) 255.1508, found 255.1507.

#### Octyl octylphosphinic acid (3c)

A solution of Octyl phosphinic acid^5*b*^1c (53.5 mg, 0.3 mmol, 1 equiv.), 1-octene 2a (33.6 mg, 0.3 mmol, 1 equiv.) and DIPB (6.7 mg, 0.03 mmol, 10 mol%) in degazed DMSO (3 mL) was irradiated under 365 nm and argon for 16 h. Ethyl acetate (30 mL) was added and the reaction mixture was washed with brine (3 × 15 mL). To the organic layer was added a 0.03 M solution of sodium bicarbonate (30 mL, 1 mmol). The aqueous layer was washed with ethyl acetate (2 × 15 mL), acidified with 1 M HCl (2 mL, 2 mmol) and extracted with ethyl acetate (30 mL). The last ethyl acetate layer was dried over Na_2_SO_4_, filtered and the solvent was removed under reduce pressure to afford compound 3c with impurities (51.8 mg, 0.19 mmol, derived from ^31^P NMR spectra on integration of all formed species). All attempts to purify the compound were unfruitful. ^31^P NMR (121 MHz, CDCl_3_): *δ* = 56.5 (s).

#### Cyclohexyl dioctylphosphinate (3d)

Following the general procedure A, using cyclohexyl octyl-*H*-phosphinate^20^1d (78 mg, 0.3 mmol, 1 equiv.), 1-octene 2a (33.6 mg, 0.3 mmol, 1 equiv.) and 10 mol% of catalyst (17.8 mg) affords 3d (81.2 mg, 0.22 mmol, 73%) as a colorless oil. ^31^P NMR (121 MHz, CDCl_3_): *δ* = 56.5 (s); ^1^H NMR (300 MHz, CDCl_3_): *δ* = 4.41–4.27 (m, 1H), 1.92–1.81 (m, 2H), 1.74–1.41 (m, 12H), 1.40–1.17 (m, 24H), 0.85 (t, *J* = 6.7 Hz, 6H); ^13^C NMR (75 MHz, CDCl_3_): *δ* = 73.2 (d, *J*_POC_ = 6.8 Hz), 34.4 (d, *J*_POCC_ = 4.3 Hz, 2C), 31.9 (2C), 31.0 (d, *J*_PCC_ = 15.1 Hz, 2C), 29.2 (2C), 29.2 (2C), 28.9 (d, *J*_PC_ = 89.9 Hz, 2C), 25.4, 23.9 (2C), 22.7 (2C), 22.1 (d, *J*_PCCC_ = 3.9 Hz, 2C), 14.2 (2C); HRMS (ESI) *m*/*z* calcd for C_22_H_46_O_2_P ([M + H]^+^) 373.3229, found 373.3227.

#### Butyl dioctylphosphinate (3e)

Following the general procedure A, using butyl octyl-*H*-phosphinate^5*d*^1e (70.3 mg, 0.3 mmol, 1 equiv.), 1-octene 2a (33.6 mg, 0.3 mmol, 1 equiv.) and 10 mol% of catalyst (17.8 mg) affords 3e (74 mg, 0.21 mmol, 71%) as a colorless oil. ^31^P NMR (121 MHz, CDCl_3_): *δ* = 57.6 (s); ^1^H NMR (300 MHz, CDCl_3_): *δ* = 3.92 (q, *J* = 6.7 Hz, 2H), 1.71–1.19 (m, 32H), 0.89 (t, *J* = 7.4 Hz, 3H), 0.84 (t, *J* = 6.7 Hz, 6H); ^13^C NMR (75 MHz, CDCl_3_): *δ* = 63.7 (d, *J*_POC_ = 6.9 Hz), 32.9 (d, *J*_POCC_ = 5.8 Hz), 31.9 (2C), 31.0 (d, *J*_PCC_ = 15.0 Hz, 2C), 29.2 (2C), 29.1 (2C), 28.0 (d, *J*_PC_ = 89.6 Hz, 2C), 22.7 (2C), 22.0 (d, *J*_PCCC_ = 3.9 Hz, 2C), 18.9, 14.1 (2C), 13.7. HRMS (ESI) *m*/*z* calcd for C_20_H_44_O_2_P ([M + H]^+^) 347.3073, found 347.3073.

#### Butyl benzyl(octyl)phosphinate (3f)

Following the general procedure A, using butyl benzyl-*H*-phosphinate^4*a*^1f (63.6 mg, 0.3 mmol, 1 equiv.), 1-octene 2a (33.6 mg, 0.3 mmol, 1 equiv.) and 50 mol% of catalyst (89 mg) affords 3f (49.6 mg, 0.15 mmol, 51%) as a colorless oil. ^31^P NMR (121 MHz, CDCl_3_): *δ* = 52.9 (s); ^1^H NMR (300 MHz, CDCl_3_): *δ* = 7.28–7.16 (m, 5H), 3.96–3.76 (m, 2H), 3.06 (d, *J*_PCH_ = 16.7 Hz, 2H), 1.60–1.39 (m, 6H), 1.35–1.13 (m, 12H), 0.84 (t, *J* = 7.3 Hz, 3H), 0.80 (t, *J* = 6.7 Hz, 3H); ^13^C NMR (75 MHz, CDCl_3_): *δ* = 132.2 (d, *J*_PCC_ = 7.2 Hz), 129.8 (d, *J*_PCCC_ = 5.6 Hz, 2C), 128.8 (d, *J*_PCCCC_ = 2.7 Hz, 2C), 126.9 (d, *J*_PCCCCC_ = 3.2 Hz), 64.3 (d, *J*_POC_ = 6.9 Hz), 36.5 (d, *J*_PC_ = 84.0 Hz), 32.9 (d, *J*_POCC_ = 5.8 Hz), 31.9, 30.9 (d, *J*_PCC_ = 15.2 Hz), 29.2, 29.1, 27.6 (d, *J*_PC_ = 92.5 Hz), 22.8, 21.8 (d, *J*_PCCC_ = 4.3 Hz), 18.9, 14.2, 13.4. HRMS (ESI) *m*/*z* calcd for C_19_H_34_O_2_P ([M + H]^+^) 325.2290, found 325.2289.

#### Cyclohexyl hydroxymethyl(octyl)phosphinate (3g)

To a solution of hypophosphorous acid (6.3 g, 48 mmol, 1 equiv.) was added paraformaldehyde (1.575 g, 52.5 mmol, 1.1 equiv.) and the reaction mixture was stirred under N_2_ for 24 h. Then, cyclohexanol (10 mL, 96 mmol, 2 equiv.) and toluene (85 mL) were added and the solution was refluxed for 16 h under Ar using a Dean-Stark apparatus. During the reaction, the formation of a gel occurred. The reaction mixture was cooled to room temperature and transferred into another round bottom flask to remove the gel and concentrated under vaccum to afford the desired hydroxymethyl-*H*-phosphinate 1g (9.4 g, 57% purity, 30.1 mmol, 63%) which was used without any further purification for the next step.^21^ A solution of compound 1g (93.7 mg, 0.3 mmol, 1 equiv.), 1-octene 2a (50.4 mg, 0.45 mmol, 1.5 equiv.) and DIPB (17.5 mg, 0.03 mmol, 10 mol%) in degazed DMSO (3 mL) under N_2_ was irradiated under 365 nm for 16 h. Ethyl acetate (30 mL) was added and the reaction mixture was washed with saturated solution of NaHCO_3_ (2 × 15 mL) and brine (2 × 15 mL). The organic layer was dried over MgSO_4_, filtered and the solvent was removed under reduce pressure. The residue was purified on a column of silica gel to afford the desired pure desired *H*-phosphinate 3g (65.2 mg, 0.23 mmol, 75%). ^31^P NMR (121 MHz, CDCl_3_): *δ* = 52.9 (s); ^1^H NMR (300 MHz, CDCl_3_): *δ* = 4.82 (s br, 1H), 4.43–4.28 (m, 1H), 3.87–3.70 (m, 2H), 1.92–1.14 (m, 24H), 0.84 (d, *J* = 6.6 Hz, 3H); ^13^C NMR (75 MHz, CDCl_3_): *δ* = 74.4 (d, *J*_POC_ = 7.2 Hz), 59.3 (d, *J*_PC_ = 104.4 Hz), 34.3 (d, *J*_POCC_ = 3.2 Hz), 34.2 (d, *J*_POCC_ = 3.3 Hz), 31.9, 31.0 (d, *J*_PCC_ = 14.9 Hz), 29.2 (2C), 26.5 (d, *J*_PC_ = 89.6 Hz), 25.2, 23.8 (2C), 22.7, 21.5 (d, *J*_PCCC_ = 4.2 Hz), 14.2; HRMS (ESI) *m*/*z* calcd for C_15_H_32_O_3_P ([M + H]^+^) 291.2083, found 291.2080.

#### Cyclohexyl phenyl(3-phenylpropyl)phosphinate (3h)

Following the general procedure A, using cyclohexyl phenyl-*H*-phosphinate 1a (67.2 mg, 0.3 mmol, 1 equiv.), allylbenzene 2b (35.4 mg, 0.3 mmol, 1 equiv.) and 50 mol% of catalyst (89 mg) affords 3h (48.3 mg, 0.14 mmol, 48%) as a colorless oil. ^31^P NMR (121 MHz, CDCl_3_): *δ* = 43.0 (s); ^1^H NMR (300 MHz, CDCl_3_): *δ* = 7.74–7.64 (m, 2H), 7.49–7.33 (m, 3H), 7.21–7.00 (m, 5H), 4.24–4.09 (m, 1H), 2.66–2.49 (m, 2H), 1.98–1.04 (m, 14H); ^13^C NMR (75 MHz, CDCl_3_): *δ* = 141.2, 132.1 (d, *J*_PC_ = 123 Hz), 132.0 (d, *J*_PCCCC_ = 2.7 Hz), 131.6 (d, *J*_PCCC_ = 9.8 Hz, 2C), 128.5 (2C), 128.5 (d, *J*_PCC_ = 12.2 Hz, 2C), 128.4 (2C), 126.0, 74.4 (d, *J*_POC_ = 6.7 Hz), 36.6 (d, *J*_PCC_ = 16.0 Hz), 34.4 (d, *J*_POCC_ = 2.9 Hz), 33.7 (d, *J*_POCC_ = 4.4 Hz), 29.8 (d, *J*_PC_ = 101 Hz), 25.2, 23.7, 23.7, 23.5 (d, *J*_PCCC_ = 3.2 Hz); HRMS (ESI) *m*/*z* calcd for C_21_H_28_O_2_P ([M + H]^+^) 342.1749, found 426.0564.

#### Cyclohexyl (3-methylbutan-2-yl)(phenyl)phosphinate (3i)

Following the general procedure A, using cyclohexyl phenyl-*H*-phosphinate 1a (67.2 mg, 0.3 mmol, 1 equiv.), 2-methylbut-2-ene 2c (22.1 mg, 0.3 mmol, 1 equiv.) and 50 mol% of catalyst (89 mg) affords 3i (24 mg, 0.08 mmol, 27%) as a colorless oil (mixture 1 : 1 of the both diastereoisomers). ^31^P NMR (121 MHz, CDCl_3_): *δ* = 42.7 (s), 46.3 (s); ^1^H NMR (300 MHz, CDCl_3_): *δ* = 7.76–7.66 (m, 2H), 7.49–7.34 (m, 3H), 4.26–4.10 (m, 1H), 2.31–1.48 (m, 7H), 1.42–1.03 (8H), 0.96–0.79 (m, 6H); ^13^C NMR (75 MHz, CDCl_3_): *δ* = 132.3 (d, *J*_PCC_ = 5.1 Hz, 2 × 0.5C), 132.2 (d, *J*_PCC_ = 5.2 Hz, 2 × 0.5C), 131.9 (d, *J*_PCCCC_ = 2.2 Hz, 0.5C), 131.9 (d, *J*_PCCCC_ = 2.2 Hz, 0.5C), 128.5 (d, *J*_PCCC_ = 3.2 Hz, 2 × 0.5C), 128.3 (d, *J*_PCCC_ = 3.1 Hz, 2 × 0.5C), 74.2 (d, *J*_POC_ = 7.2 Hz, 0.5C), 74.0 (d, *J*_POC_ = 7.1 Hz, 0.5C), 39.6 (d, *J*_PC_ = 100 Hz 2 × 0.5C), 34.4 (d, *J*_POCC_ = 2.4 Hz, 0.5C), 34.4 (d, *J*_POCC_ = 2.3 Hz, 0.5C), 33.8 (d, *J*_POCC_ = 1.9 Hz, 0.5C), 33.8 (d, *J*_POCC_ = 1.9 Hz, 0.5C), 26.8 (d, *J*_PCCC_ = 2.8 Hz, 0.5C), 26.1 (0.5C), 25.4 (2 × 0.5C), 23.8 (2 × 0.5C), 23.7 (2 × 0.5C), 22.6 (d, *J*_PCC_ = 6.1 Hz, 0.5C), 22.4 (d, *J*_PCC_ = 8.7 Hz, 0.5C), 18.3 (d, *J*_PCC_ = 3.2 Hz, 0.5C), 17.7 (d, *J*_PCCC_ = 2.2 Hz, 0.5C), 7.7 (0.5C), 6.9 (d, *J*_PCC_ = 4.6 Hz, 0.5C) (1 signal missing); HRMS (ESI) *m*/*z* calcd for C_17_H_28_O_2_P ([M + H]^+^) 295.1821, found 295.1819.

#### Cyclohexyl (5-bromopentyl)(phenyl)phosphinate (3j)

Following the general procedure A, using cyclohexyl phenyl-*H*-phosphinate 1a (67.2 mg, 0.3 mmol, 1 equiv.), 5-bromo-1-pentene 2d (46.6 mg, 0.3 mmol, 1 equiv.) and 10 mol% of catalyst (17.8 mg) affords 3j (43 mg, 0.12 mmol, 38%) as a colorless oil. ^31^P NMR (121 MHz, CDCl_3_): *δ* = 42.7 (s); ^1^H NMR (300 MHz, CDCl_3_): *δ* = 7.82–7.72 (m, 2H), 7.56–7.39 (m, 3H), 4.31–4.18 (m, 1H), 3.34 (t, *J* = 6.8 Hz, 2H), 2.06–1.11 (m, 18H); ^13^C NMR (75 MHz, CDCl_3_): *δ* = 132.2 (d, *J*_PC_ = 123 Hz), 132.1 (d, *J*_PCCCC_ = 2.7 Hz), 131.7 (d, *J*_PCCC_ = 9.7 Hz, 2C), 128.6 (d, *J*_PCC_ = 12.3 Hz, 2C), 74.5 (d, *J*_POC_ = 6.7 Hz), 34.5 (d, *J*_POCC_ = 2.9 Hz), 33.8 (d, *J*_POCC_ = 4.4 Hz), 33.5, 32.4, 30.3 (d, *J*_PC_ = 101 Hz), 29.3 (d, *J*_PCC_ = 15.7 Hz), 25.3, 23.8, 23.8, 21.2 (d, *J*_PCCC_ = 3.6 Hz); HRMS (ESI) *m*/*z* calcd for C_17_H_27_BrO_2_P ([M + H]^+^) 372.0854, found.

#### Cyclohexyl phenyl(3-hydroxypropyl)phosphinate (3k)

Following the general procedure A, using cyclohexyl phenyl-*H*-phosphinate 1a (67.2 mg, 0.3 mmol, 1 equiv.), allyl alcohol 2e (17.4 mg, 0.3 mmol, 1 equiv.) and 50 mol% of catalyst (89 mg) affords 3k (47.0 mg, 0.168 mmol, 56%) as a colorless oil. ^31^P NMR (121 MHz, CDCl_3_): *δ* = 44.9 (s); ^1^H NMR (300 MHz, CDCl_3_): *δ* = 7.84–7.72 (m, 2H), 7.58–7.40 (m, 3H), 4.32–4.18 (m, 1H), 3.63–3.70 (m, 2H), 2.12–1.12 (m, 15H); ^13^C NMR (75 MHz, CDCl_3_): *δ* = 132.3 (d, *J*_PCCCC_ = 2.6 Hz), 131.7 (d, *J*_PCCC_ = 9.9 Hz, 2C), 131.6 (d, *J*_PC_ = 123.9 Hz), 128.7 (d, *J*_PCC_ = 12.4 Hz, 2C), 74.9 (d, *J*_POC_ = 6.7 Hz), 62.8 (d, *J*_PCC_ = 11.0 Hz), 34.4 (d, *J*_POCC_ = 2.7 Hz), 33.8 (d, *J*_POCC_ = 4.3 Hz), 27.9 (d, *J*_PC_ = 101.3 Hz), 25.4 (d, *J*_PCCC_ = 3.8 Hz), 25.2, 23.7, 14.3; HRMS (ESI) *m*/*z* calcd for C_15_H_24_O_3_P ([M + H]^+^) 283.1457, found 283.1455.

#### Cyclohexyl (4-hydroxybutyl) (phenyl) phosphinate (3l)

Following the general procedure A, using cyclohexyl phenyl-*H*-phosphinate 1a (67.2 mg, 0.3 mmol, 1 equiv.), 3-buten-1-ol 2f (21.6 mg, 0.3 mmol, 1 equiv.) and 50 mol% of catalyst (89 mg) affords 3l (61 mg, 0.207 mmol, 69%) as a colorless oil. ^31^P NMR (121 MHz, CDCl_3_): *δ* = 43.4 (s); ^1^H NMR (300 MHz, CDCl_3_): *δ* = 7.81–7.72 (m, 2H), 7.58–7.41 (m, 3H), 4.30–4.16 (m, 1H), 3.65–3.57 (m, 2H), 2.06–1.13 (m, 17H); ^13^C NMR (75 MHz, CDCl_3_): *δ* = 132.2 (d, *J*_PCCCC_ = 2.6 Hz), 132.0 (d, *J*_PC_ = 123.0 Hz), 131.7 (d, *J*_PCCC_ = 9.8 Hz, 2C), 128.6 (d, *J*_PCC_ = 12.3 Hz, 2C), 74.6 (d, *J*_POC_ = 6.8 Hz), 62.0, 34.5 (d, *J*_POCC_ = 2.8 Hz), 33.8 (d, *J*_POCC_ = 4.4 Hz), 33.5 (d, *J*_PCC_ = 14 Hz), 29.8 (d, *J*_PC_ = 101 Hz), 25.3, 23.8, 18.2 (d, *J*_PCCC_ = 3.6 Hz) (1 signal missing); HRMS (ESI) *m*/*z* calcd for C_16_H_26_O_3_P ([M + H]^+^) 297.1614, found 297.1612.

#### Octyl phosphinic acid (1c)

Following the general procedure B, using 50% aqueous solution of hypophosphorous acid (79.2 mg, 0.6 mmol, 2 equiv.), 1-octene 2a (33.6 mg, 0.3 mmol, 1 equiv.) and 20 mol% of catalyst (15.5 mg, 0.06 mmol) affords 1b (29 mg, 0.165 mmol, 55%) as a colorless oil. ^31^P NMR (121 MHz, CDCl_3_): *δ* = 37.7 (d, *J*_PH_ = 544 Hz); ^1^H NMR (300 MHz, CDCl_3_): *δ* = 12.53 (br s, 1H), 7.11 (d, *J*_PH_ = 544 Hz, 1H), 1.85–1.71 (m, 2H), 1.70–1.53 (m, 2H), 1.49–1.24 (m, 10H), 0.91 (t, *J* = 6.7 Hz, 3H); ^13^C NMR (75 MHz, CDCl_3_): *δ* = 31.9, 30.5 (d, *J*_PCC_ = 16 Hz), 29.4 (d, *J*_PC_ = 94 Hz), 29.2, 29.1, 22.7, 20.5 (d, *J*_PCCC_ = 2.7 Hz), 14.2; HRMS (ESI) *m*/*z* calcd for C_8_H_20_O_2_P ([M + H]^+^) 179.1195, found 179.1190.

#### 3-Phenylpropyl phosphinic acid (1h)

Following the general procedure B, using 50% aqueous solution of hypophosphorous acid (79.2 mg, 0.6 mmol, 2 equiv.), allylbenzene 2b (35.4 mg, 0.3 mmol, 1 equiv.) and 20 mol% of catalyst (15.5 mg, 0.06 mmol) affords 1h (34.7 mg, 0.189 mmol, 63%) as a colorless oil. ^31^P NMR (121 MHz, CDCl_3_): *δ* = 34.2 (d, *J*_PH_ = 541 Hz); ^1^H NMR (300 MHz, CDCl_3_): *δ* = 7.32–7.24 (m, 2H), 7.23–7.10 (m, 3H), 7.07 (d, *J*_PH_ = 541 Hz, 1H), 2.76–2.63 (m, 2H), 2.02–1.62 (m, 4H); ^13^C NMR (75 MHz, CDCl_3_): *δ* = 140.9, 128.6 (4C), 126.4, 36.4 (d, *J*_PCC_ = 16 Hz), 28.8 (d, *J*_PC_ = 94 Hz), 22.4 (d, *J*_PCCC_ = 2.9 Hz); HRMS (ESI) *m*/*z* calcd for C_9_H_14_O_2_P ([M + H]^+^) 185.0725, found 185.0720.

#### 1,2-Dimethylpropyl phosphinic acid (1i)

Following the general procedure B, using 50% aqueous solution of hypophosphorous acid (79.2 mg, 0.6 mmol, 2 equiv.), 2-methylbut-2-ene 2c (21 mg, 0.3 mmol, 1 equiv.) and 20 mol% of catalyst (15.5 mg, 0.06 mmol) affords 1i (12.6 mg, 0.09 mmol, 31%) as a colorless oil. ^31^P NMR (121 MHz, CDCl_3_): *δ* = 44.0 (d, *J*_PH_ = 534 Hz); ^1^H NMR (300 MHz, CDCl_3_): *δ* = 8.90 (br s, 1H), 7.01 (d, *J*_PH_ = 534 Hz, 1H), 2.22–2.04 (m, 1H), 1.72–1.54 (m, 1H), 1.18–0.95 (m, 9H); ^13^C NMR (75 MHz, CDCl_3_): *δ* = 39.5 (d, *J*_PC_ = 94 Hz), 27.1, 21.6 (d, *J*_PCC_ = 12 Hz), 19.0 (d, *J*_PCC_ = 5.9 Hz), 7.3; HRMS (ESI) *m*/*z* calcd for C_5_H_14_O_2_P ([M + H]^+^) 137.0725, found 137.0720.

#### Cyclohexyl phosphinic acid (1j)

Following the general procedure B, using 50% aqueous solution of hypophosphorous acid (79.2 mg, 0.6 mmol, 2 equiv.), cyclohexene (21 mg, 0.3 mmol, 1 equiv.) and 20 mol% of catalyst (15.5 mg, 0.06 mmol) affords 1k (21.7 mg, 0.147 mmol, 49%) as a colorless oil. ^31^P NMR (121 MHz, CDCl_3_): *δ* = 42.8 (d, *J*_PH_ = 537 Hz); ^1^H NMR (300 MHz, CDCl_3_): *δ* = 6.82 (d, *J*_PH_ = 537 Hz, 1H), 2.01–1.54 (m, 6H), 1.39–1.14 (m, 5H); ^13^C NMR (75 MHz, CDCl_3_): *δ* = 37.6 (d, *J*_PC_ = 95 Hz), 26.0 (2C), 25.8, 24.0 (2C); HRMS (ESI) *m*/*z* calcd for C_6_H_14_O_2_P ([M + H]^+^) 149.0725, found 149.0720.

## Conclusions

In conclusion, we developed an efficient UV-mediated hydrophosphinylation of unactivated alkenes with *H*-phosphinate under free radical conditions giving a straightforward access to a wide range of phosphorous compounds providing a metal free alternative for the preparation and functionalization of P(O)–H bonds. This reaction can be carried out using an ionic liquid soluble photosensitizer while maintaining good yield. Furthermore, the reaction with hypophosphorous acid is disclosed for the first time. Finally, a continuous flow hydrophosphinylation was also reported which allowed faster reaction time with high yields, highlighting the potential of our methodology for rapid scale up and in-line synthesis. Taking into account the simplicity of our reaction conditions we believe this procedure will be appealing for further chemical and pharmaceutical applications.

## Conflicts of interest

There are no conflicts to declare.

## Supplementary Material

RA-008-C7RA12977G-s001
