# Supplementary material for: UV-mediated hydrophosphinylation of unactivated alkenes with phosphinates under batch and flow conditions
Source: RSC Adv. 2018 Feb 23;8(15):8385–92. doi: 10.1039/c7ra12977g (PMC9078595; doi:10.1039/c7ra12977g)

Electronic Supplementary Material (ESI) for RSC Advances.
This journal is © The Royal Society of Chemistry 2018

Supporting Information

UV mediated hydrophosphinylation of unactivated alkenes with phosphinates in batch and flow

Fabien Gelat*, Maxime Roger, Christophe Penverne, Ahmed Mazzah, Christian Rolando and Laetitia Chausset-Boissarie*

Materials and methods 2
Optimisation of hydrophosphinylation of Alkenes with hypophosphorous acid 3
Copies of NMR spectra 4

Materials and methods

All reagents were purchased from commercial suppliers (Strem Chemicals Inc., Sigma-Aldrich or Alfa Aesar) and were used without further purification unless otherwise indicated. Acetone and methanol (Chromasolv®) were stored with active molecular sieves (3 Å) for 24 hours before usage. Thin-layer chromatography (TLC) were performed on Silica gel 60 F254 plates (Merck) and visualized under UV (254 nm) or by staining with potassium permanganate or phosphomolybdic acid.
The purification of the obtained products was performed by flash chromatography using PuriFlash® 215 equipped with UV 1 (90-840 nm) and ELSD detectors purchased from Interchim. Pre-packed flash chromatography Silica HP columns (30µm) were used.
NMR spectra were recorded on a Bruker AVANCE 300 spectrometer at 300 MHz (75 MHz). Chemical shifts are given in parts per million relative to solvent signal. The following abbreviations are used for the proton spectra multiplicities: s, singlet; d, doublet; t, triplet; q, quartet; m, multiplet. Coupling constant are reported in hertz (Hz). High-resolution mass spectra (HRMS) were performed on a Thermo LTQ orbitrap mass spectrometer.

Flow System

A Mikroglas Dwell Device® microreactor made of Foturan glass (dimensions: 1.15 m × 2000 µm × 500 µm).

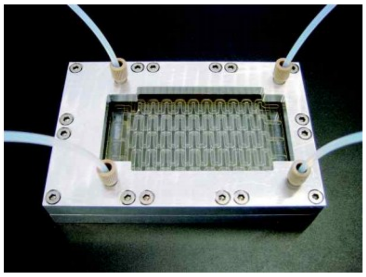

Figure 1. (i) Mikroglas Dwell Device®

The different flow rates of the performed reactions were regulated using a Harvard Apparatus (Holliston, MA, USA) PHD ULTRA CP syringe pump.
For UV Irradiation: UV LEDs (Omnicure® AC475 of λ =365 nm and irradiance up to 250 mW.cm^{-2}) purchased from Lumen were used.

Optimisation of hydrophosphinylation of Alkenes with hypophosphorous acid

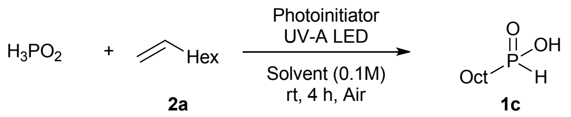

Entry[a]	Photoinitiator	Ratio H_3PO_2/2	Solvent	Yield (%)[b]
1	4,4'-DMBP[c] 1eq	2:1	DMF	70
2	4,4'-DMBP 1eq	2:1	CH_3CN	50
3	4,4'-DMBP 1eq	2:1	*i*PrOH	80
4	4,4'-DMBP 1eq	2:1	DMSO	80
5	4,4'-DMBP 0.5eq	2:1	DMSO	76
6	4,4'-DMBP 0.2eq	2:1	DMSO	70
7	DMPA[d] 1 eq	1:1	DMF	40
8	DMPA 1 eq	2:1	DMF	70
9	DMPA 1 eq	2:1	DMSO	75
**10**	**DMPA 0.2 eq**	**2:1**	**DMSO**	**80**
11	Eosin Y	2:1	DMF	-
12	Rhodamine	2:1	DMF	-
13[e]	4,4'-DMBP 0.2eq	2:1	DMSO	-
14[f]	4,4'-DMBP 0.2eq	2:1	DMSO	-
15[g]	4,4'-DMBP 0.2eq	2:1	DMSO	60
16	-	2:1	DMSO	-
17[h]	4,4'-DMBP 0.2eq	2:1	DMSO	60
18[i]	4,4'-DMBP 0.2eq	2:1	DMSO	-
19[j]	4,4'-DMBP 0.2eq	2:1	DMSO	-

[a]Reaction condition: H_3PO_2 (x mmol), **2a** (0.3 mmol), Photoinitiator (x equiv), solvent ([0.1 M]), under Air and UV-A LED irradiation (λ = 365 ± 15 nm, 230 mW.cm^{-2}) at room temperature. [b]Derived from ^{31}P crude NMR spectra on integration of all formed species. [c]4,4'-DMBP = 4,4'-dimethoxybenzophenone. [d]DMPA = 2,2-dimethoxy-2-phenylacetophenone. [e]Without irradiation. [f]Under ambient atmosphere. [g]Under O_2 atmosphere. [h]with TEMPO. [h]Irradiation with black light. [h]Irradiation with white LEDs.

Copies of NMR spectra

Cyclohexyl octyl(phenyl)phosphinate 3a

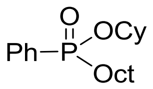

^{31}P-^{1}H decoupled NMR Spectrum (CDCl$_3$, 121 MHz)

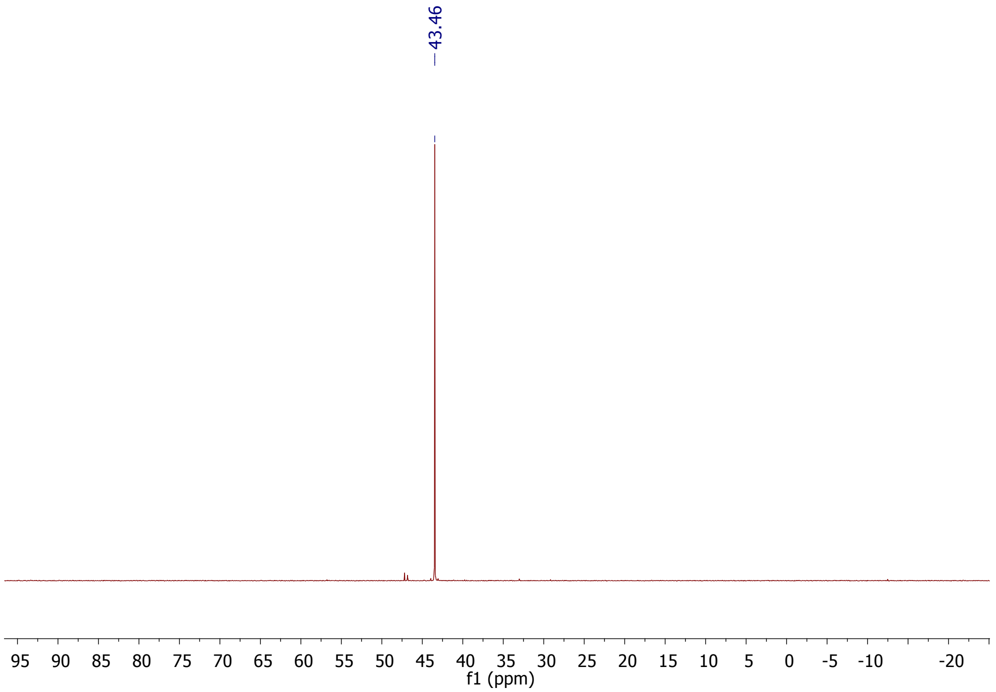

^{31}P-^{1}H coupled NMR Spectrum (CDCl$_3$, 121 MHz)

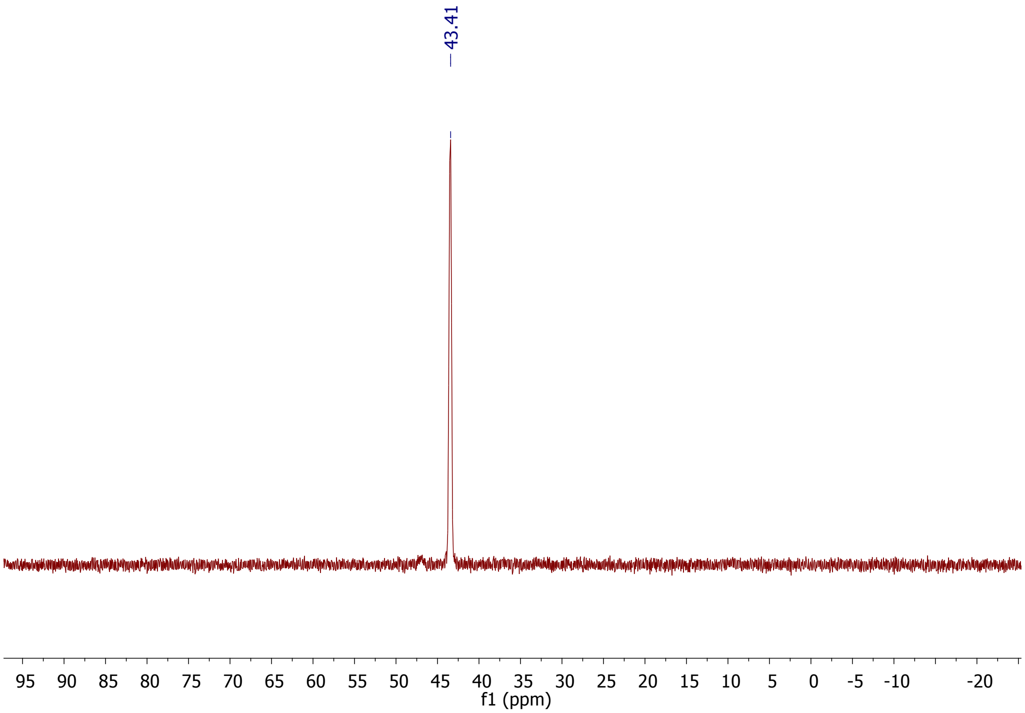

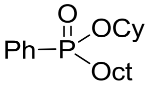

^1H NMR Spectrum ($CDCl_3$, 300 MHz)

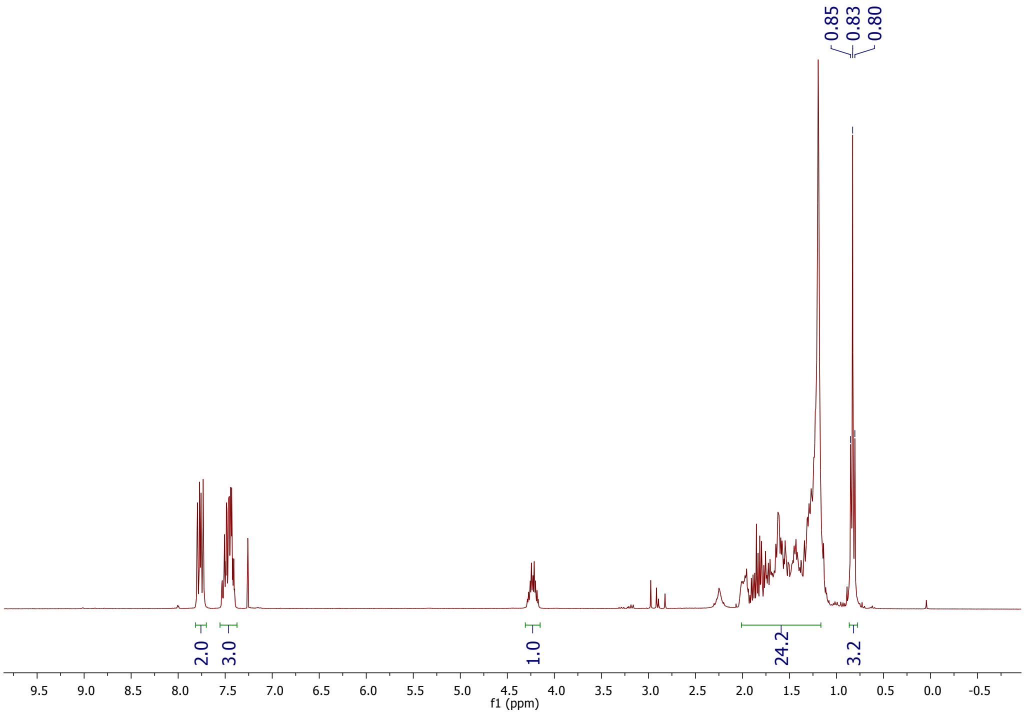

^{13}C NMR Spectrum ($CDCl_3$, 75 MHz)

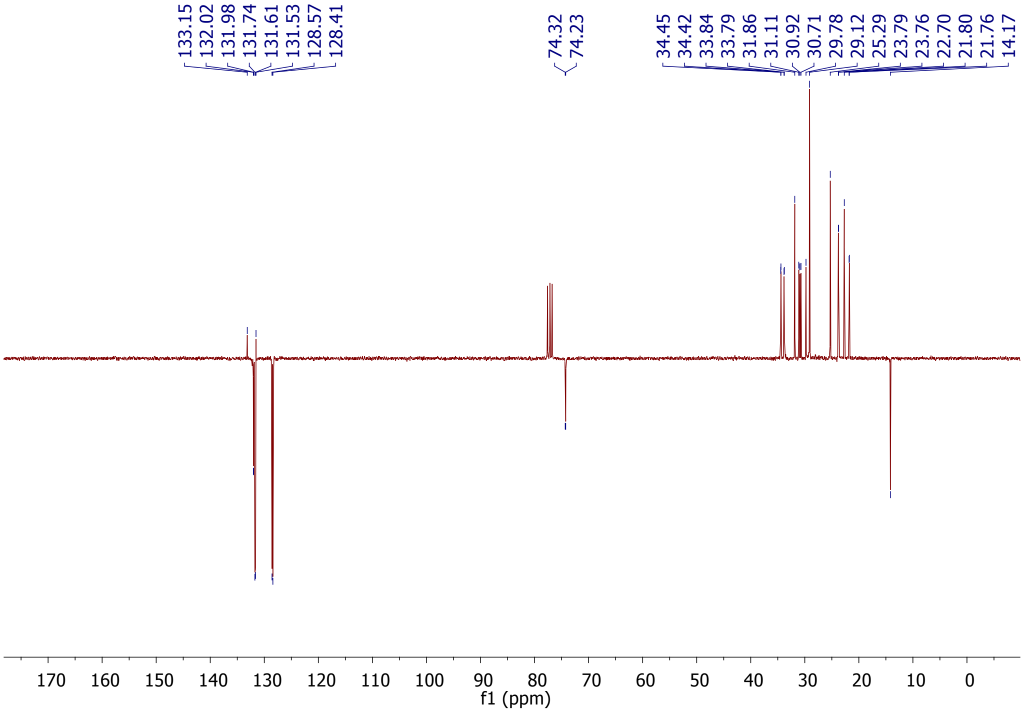

Octyl phenylphosphinic acid 3b

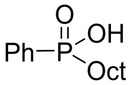

^{31}P-^{1}H decoupled NMR Spectrum ($CDCl_3$, 121 MHz)

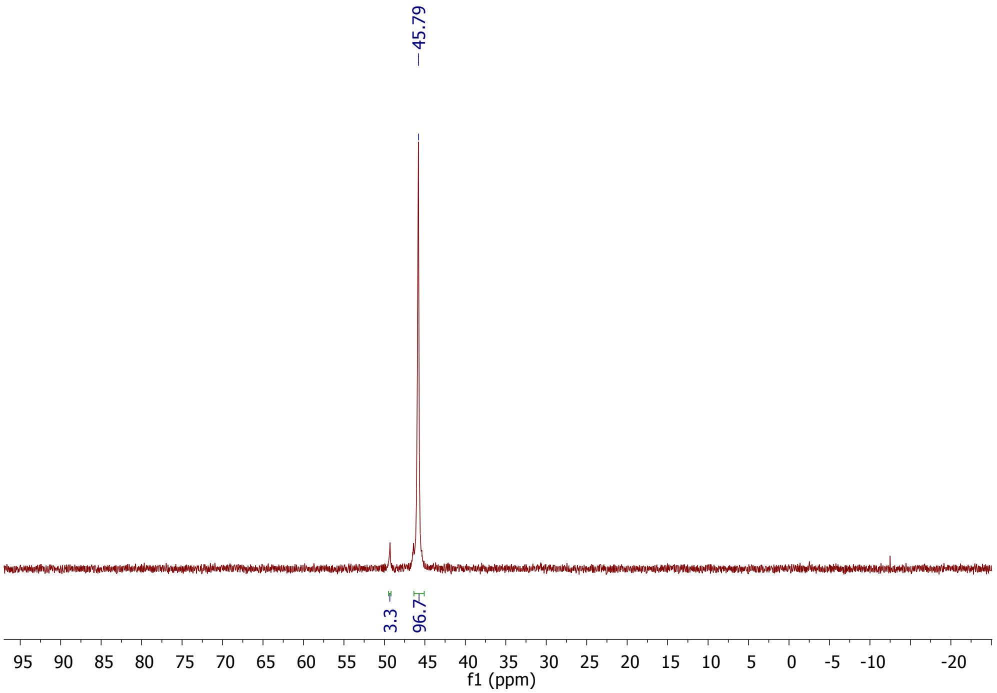

^{31}P-^{1}H coupled NMR Spectrum ($CDCl_3$, 121 MHz)

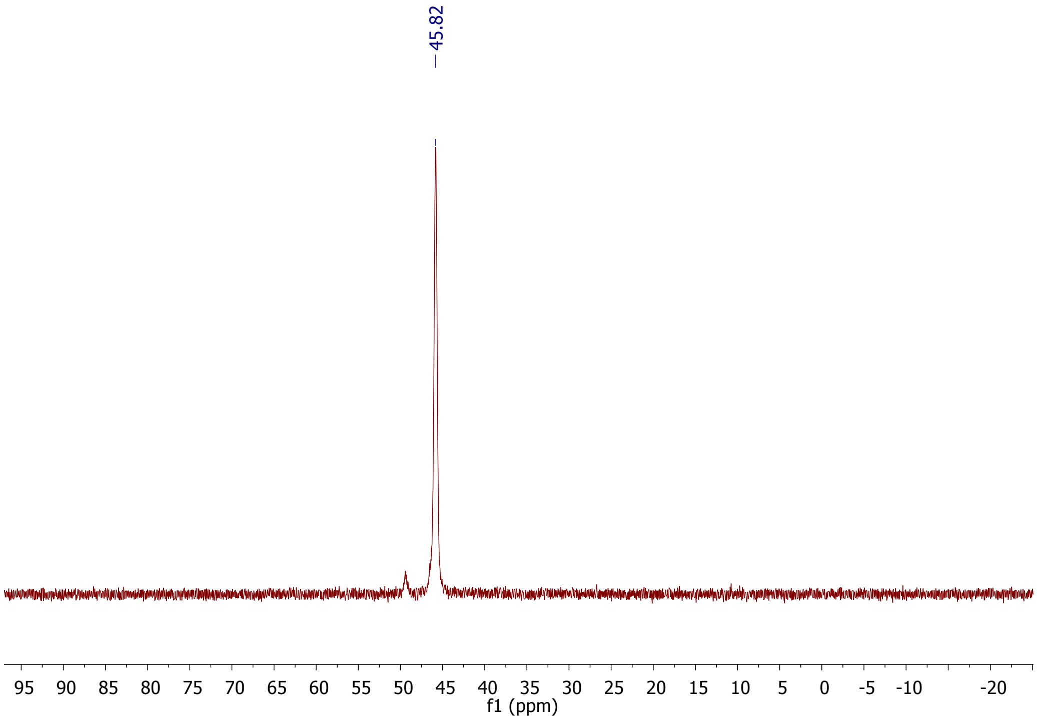

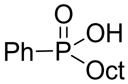

^{1}H NMR Spectrum ($CDCl_3$, 300 MHz)

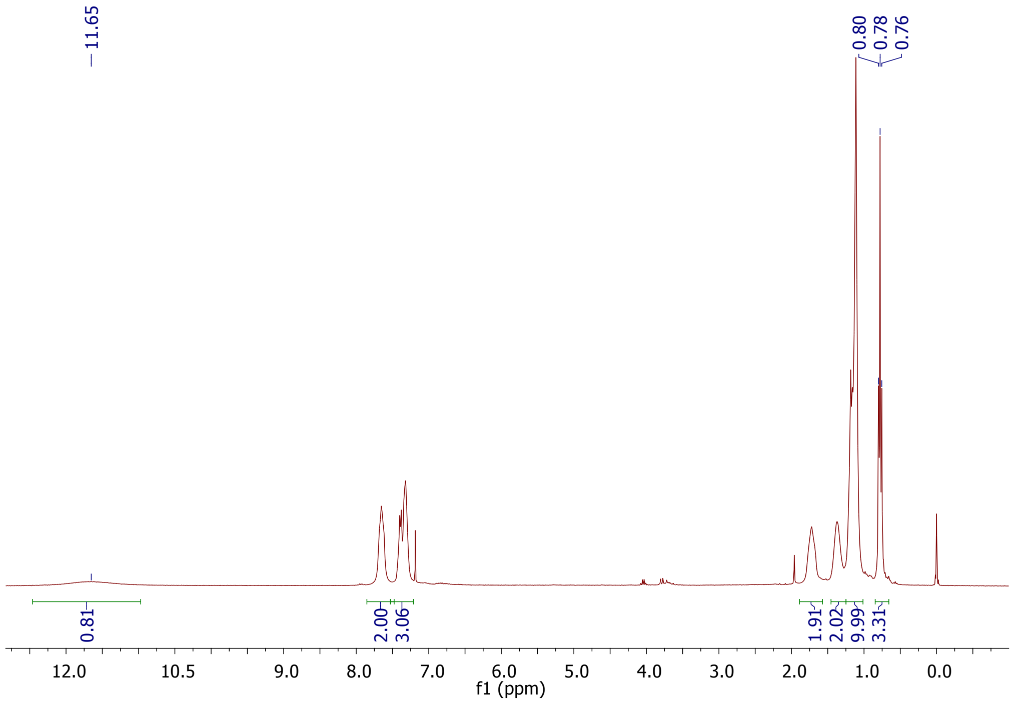

^{13}C NMR Spectrum ($CDCl_3$, 75 MHz)

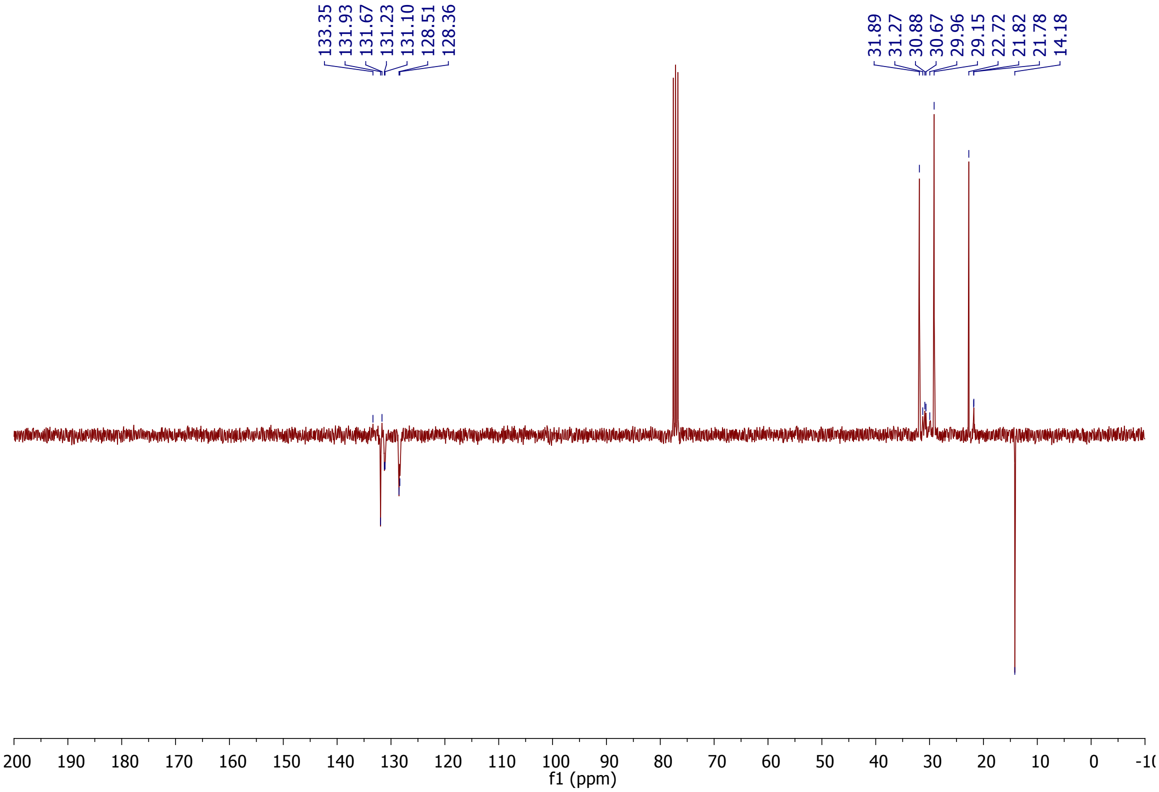

Cyclohexyl (di-octyl)phosphinate 3d

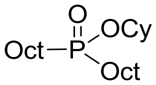

^{31}P-^{1}H decoupled NMR Spectrum ($CDCl_3$, 121 MHz)

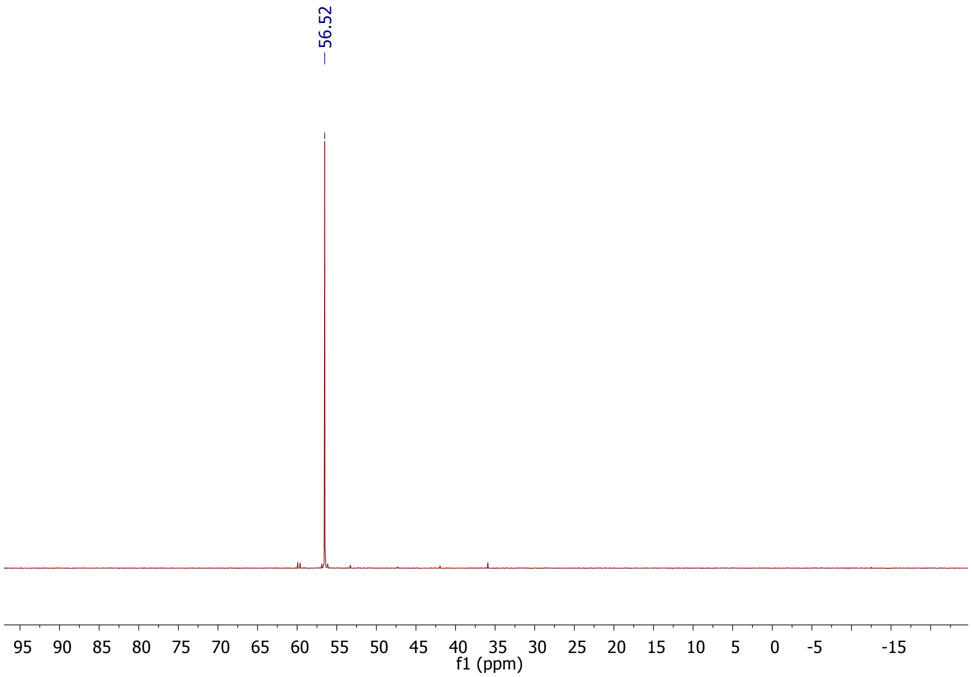

^{31}P-^{1}H coupled NMR Spectrum ($CDCl_3$, 121 MHz)

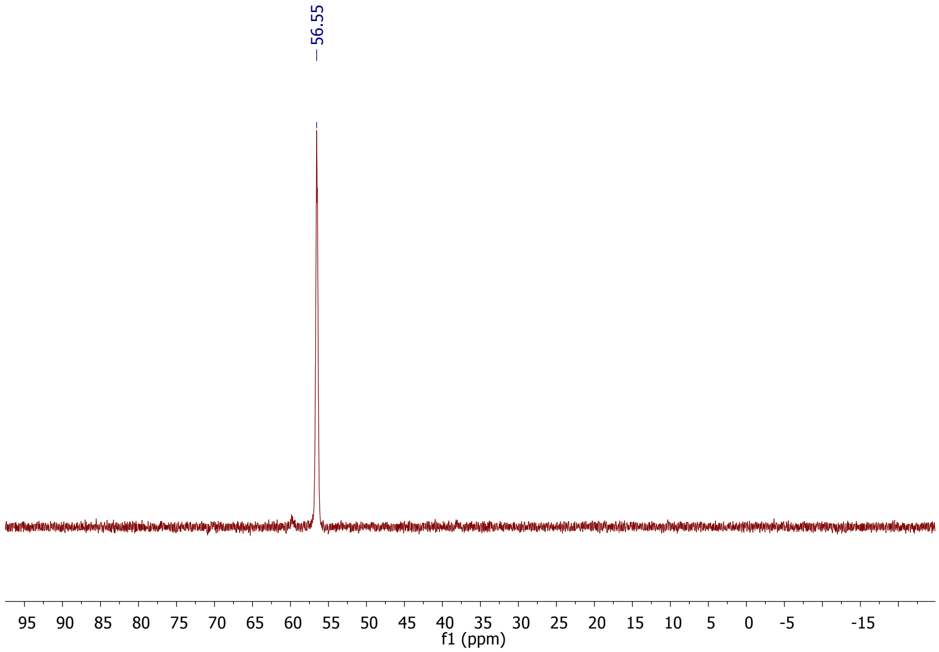

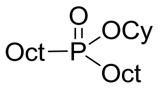

^{1}H NMR Spectrum ($CDCl_3$, 300 MHz)

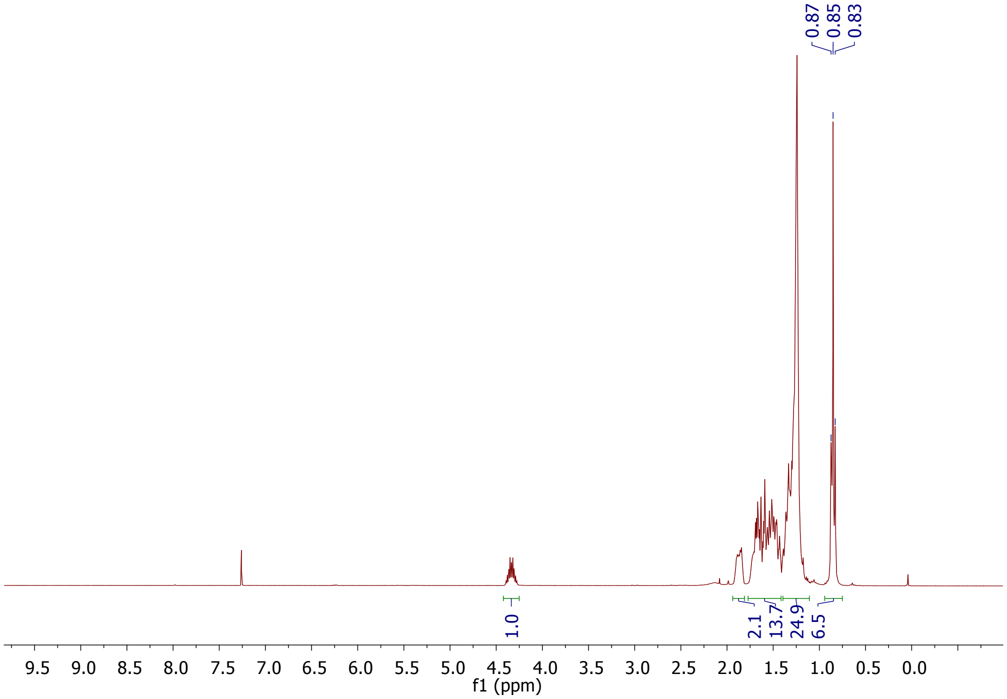

^{13}C NMR Spectrum ($CDCl_3$, 75 MHz)

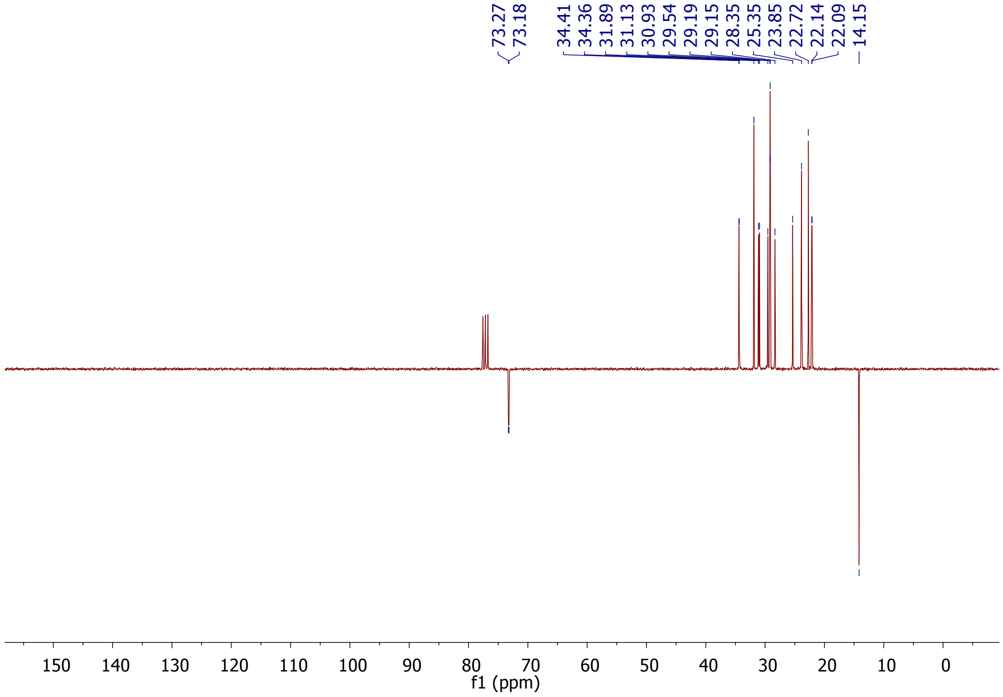

Butyl (di-octyl)phosphinate 3e

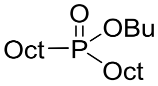

^{31}P-^{1}H decoupled NMR Spectrum ($CDCl_3$, 121 MHz)

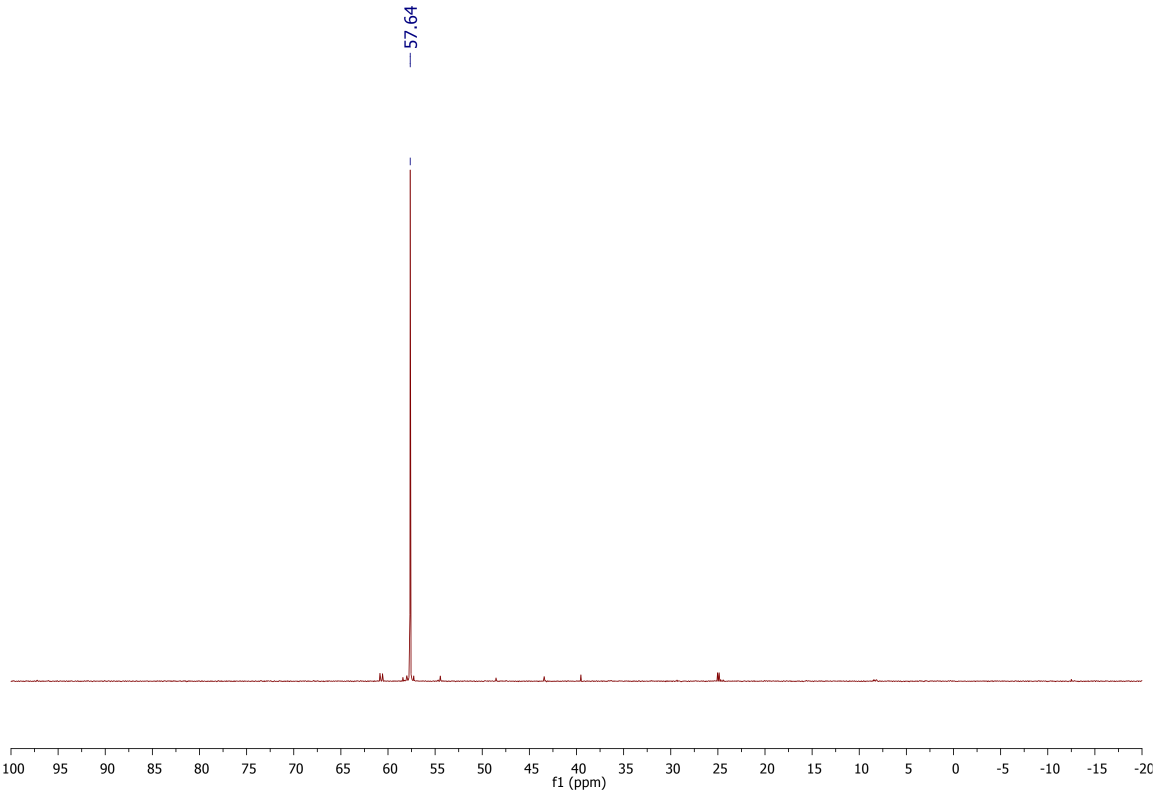

^{31}P-^{1}H coupled NMR Spectrum ($CDCl_3$, 121 MHz)

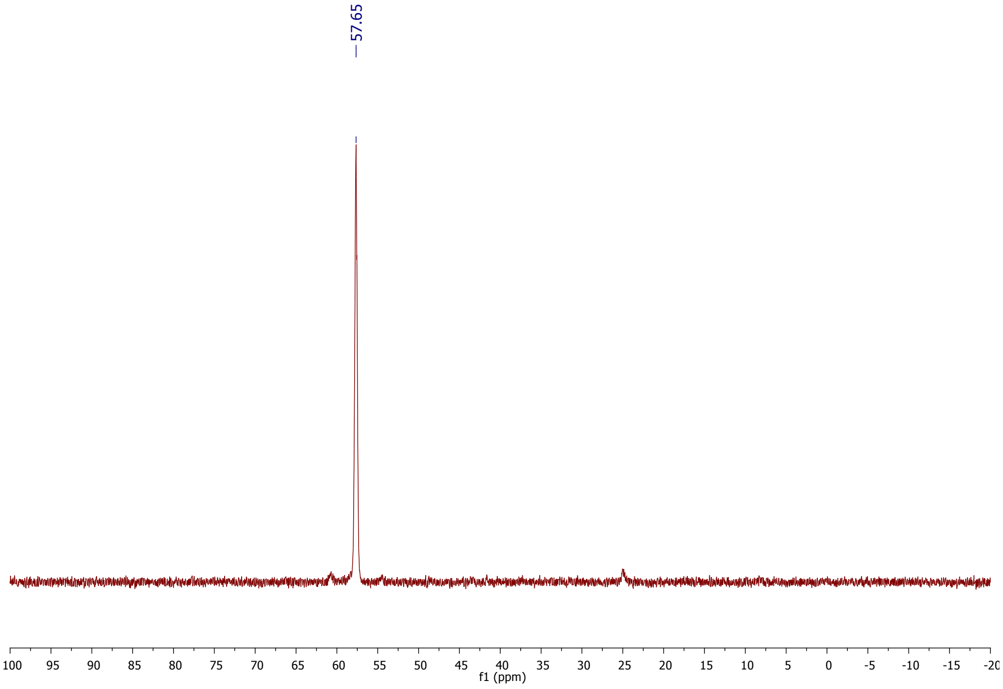

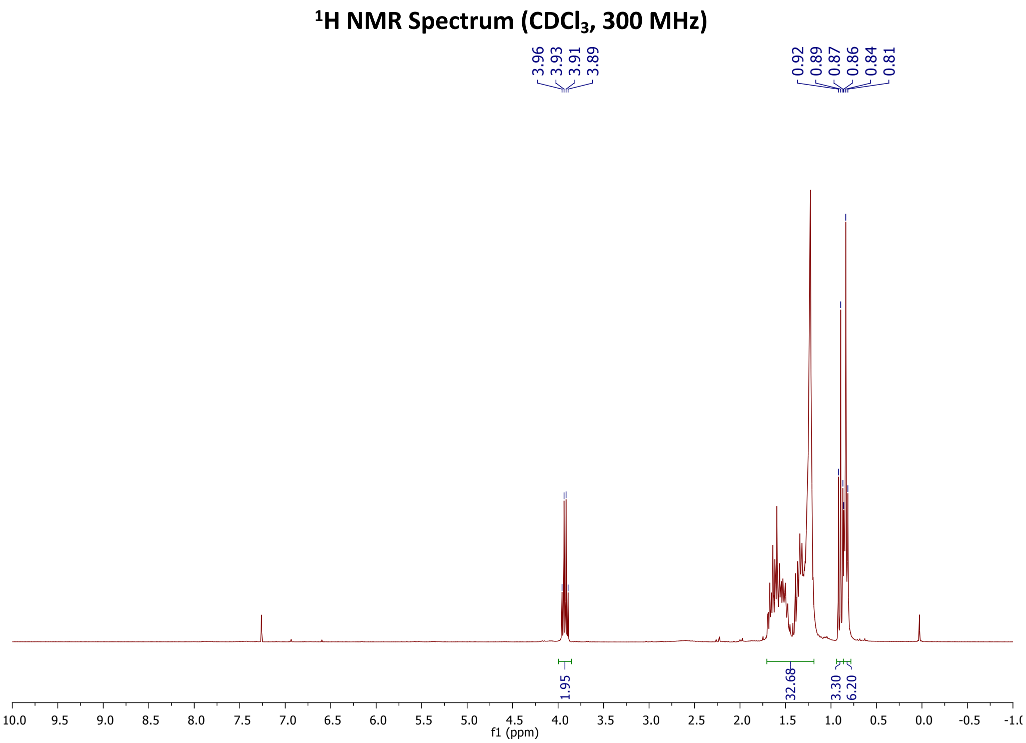

¹H NMR Spectrum (CDCl3, 300 MHz)
3.96
3.93
3.91
3.89
0.92
0.89
0.87
0.86
0.84
0.81
1.95
32.68
3.30
6.20
10.0 9.5 9.0 8.5 8.0 7.5 7.0 6.5 6.0 5.5 5.0 4.5 4.0 3.5 3.0 2.5 2.0 1.5 1.0 0.5 0.0 -0.5
f1 (ppm)

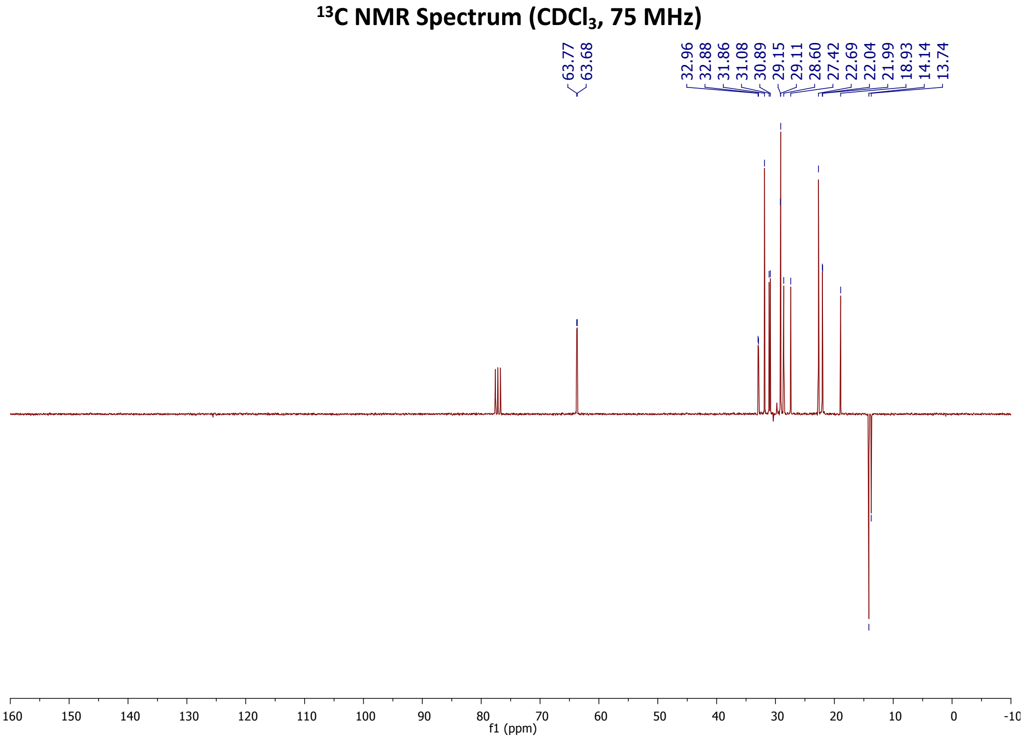

¹³C NMR Spectrum (CDCl3, 75 MHz)
63.77
63.68
32.96
32.88
31.86
31.08
30.89
29.15
29.11
28.60
27.42
22.69
22.04
21.99
18.93
14.14
13.74
160 150 140 130 120 110 100 90 80 70 60 50 40 30 20 10 0
f1 (ppm)

Butyl benzyl(octyl)phosphinate 3f:

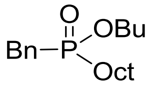

^{31}P-^{1}H decoupled NMR Spectrum (CDCl$_3$, 121 MHz)

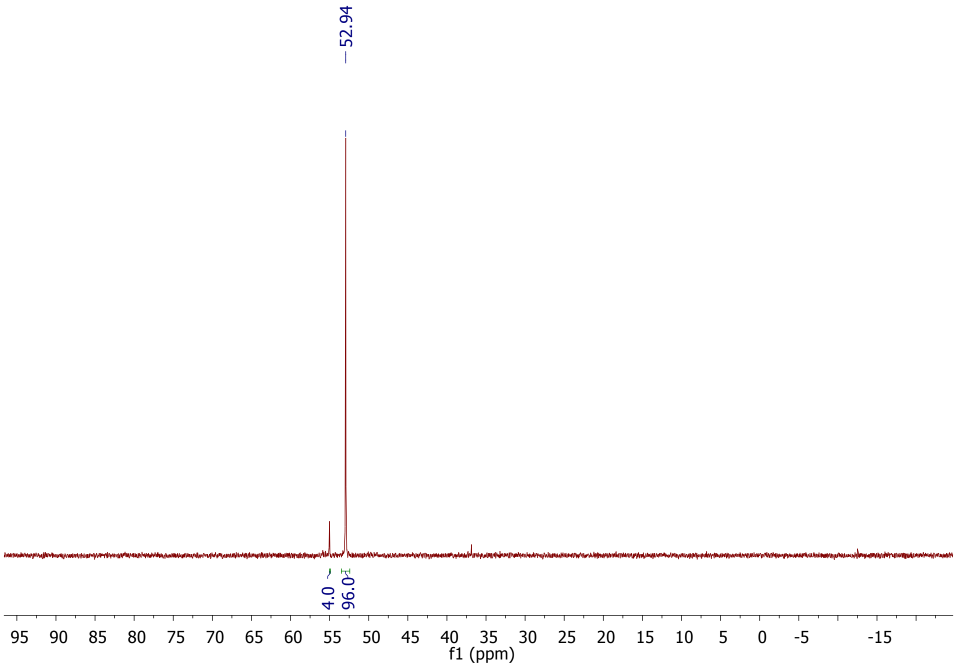

^{31}P-^{1}H coupled NMR Spectrum (CDCl$_3$, 121 MHz)

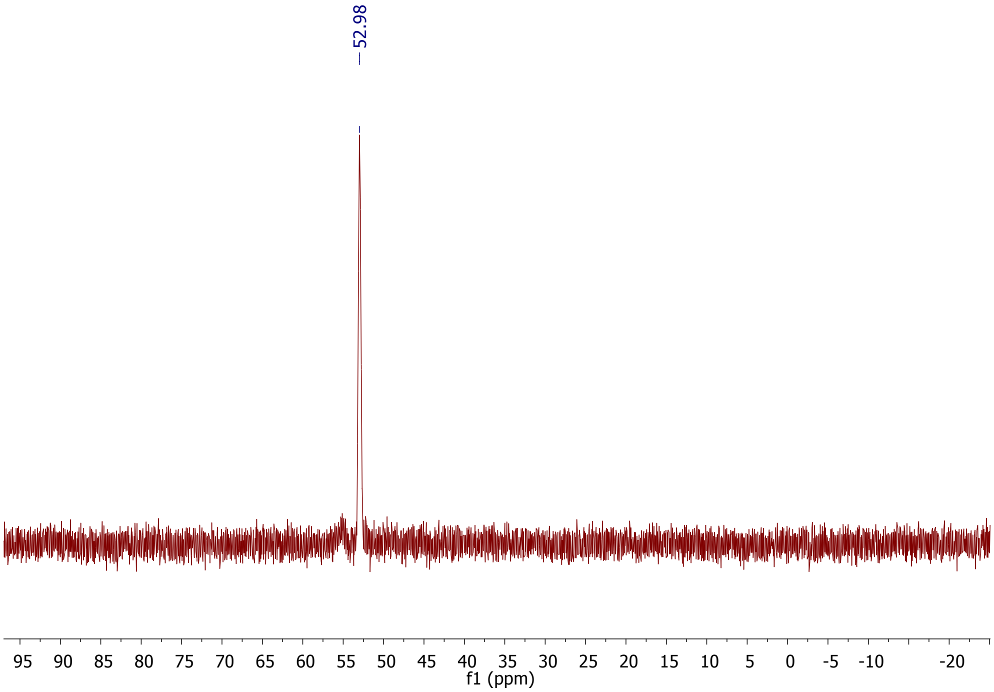

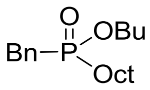

^{1}H NMR Spectrum ($CDCl_3$, 300 MHz)

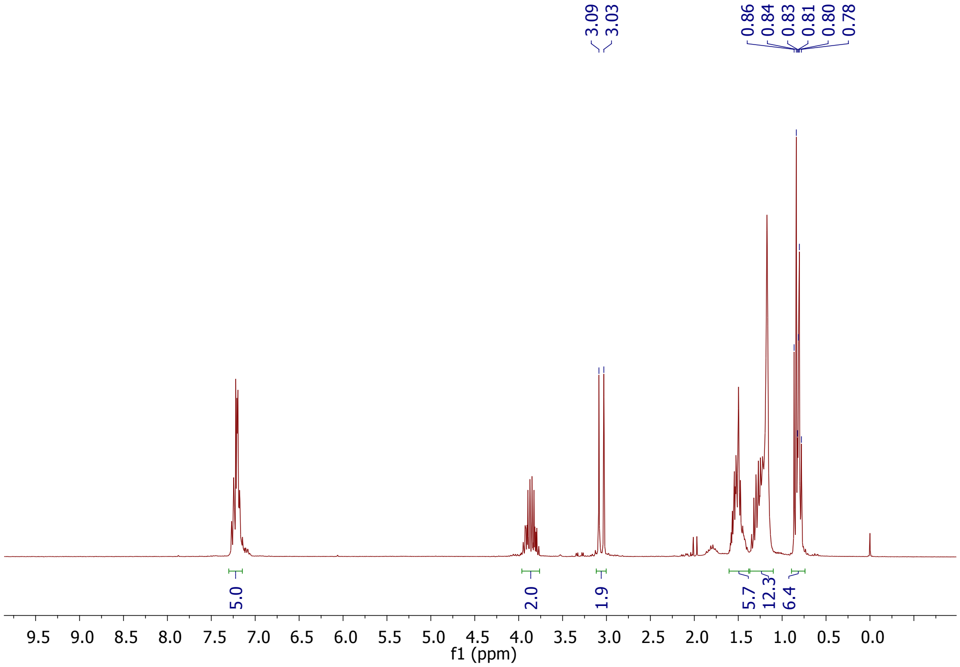

^{13}C NMR Spectrum ($CDCl_3$, 75 MHz)

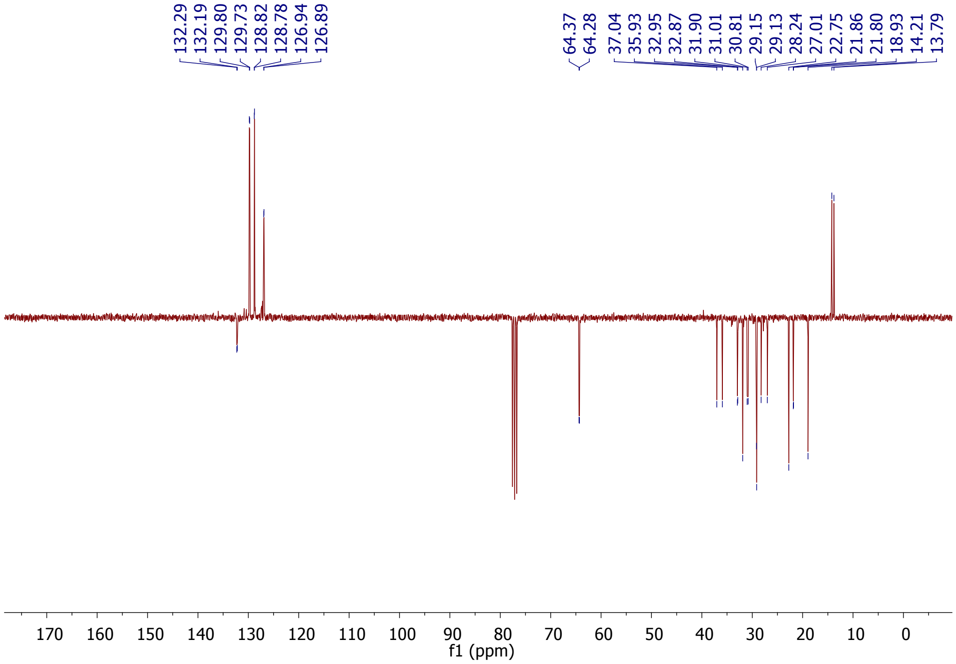

Cyclohexyl hydroxymethyl(octyl)phosphinate 3g

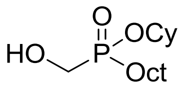

^{31}P-^{1}H decoupled NMR Spectrum ($CDCl_3$, 121 MHz)

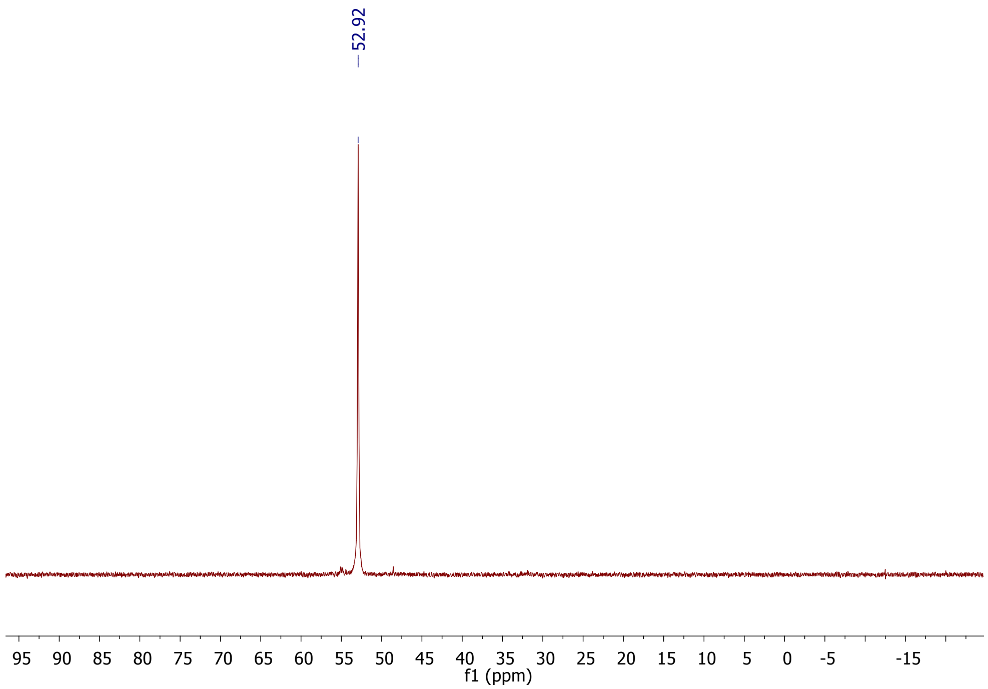

^{31}P-^{1}H coupled NMR Spectrum ($CDCl_3$, 121 MHz)

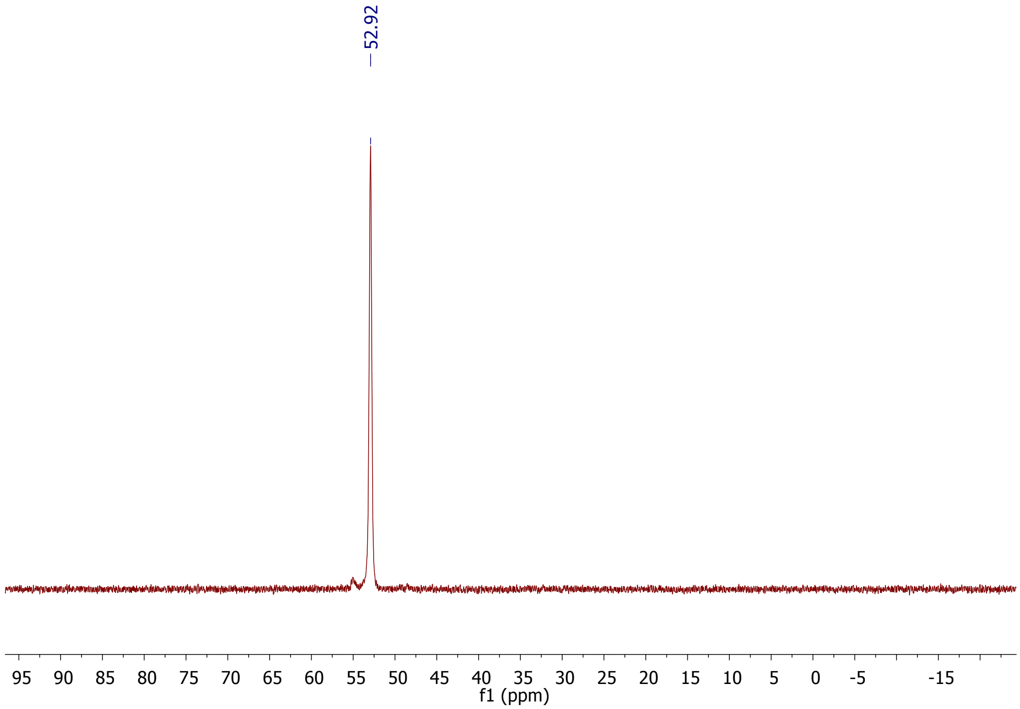

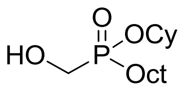

¹H NMR Spectrum (CDCl$_3$, 300 MHz)

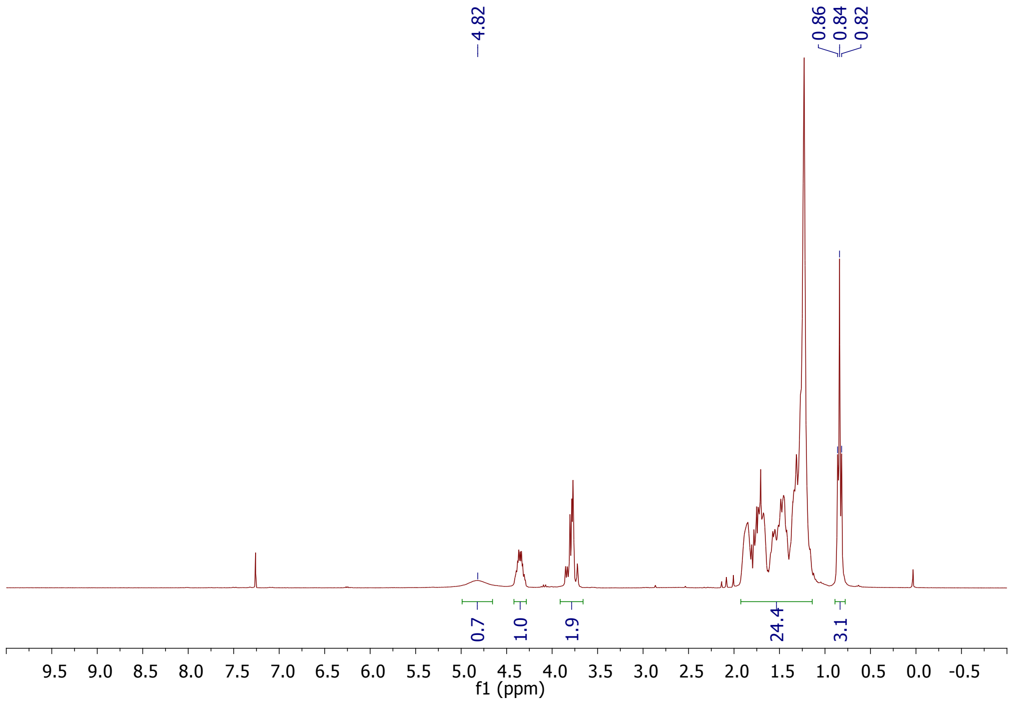

¹³C NMR Spectrum (CDCl$_3$, 75 MHz)

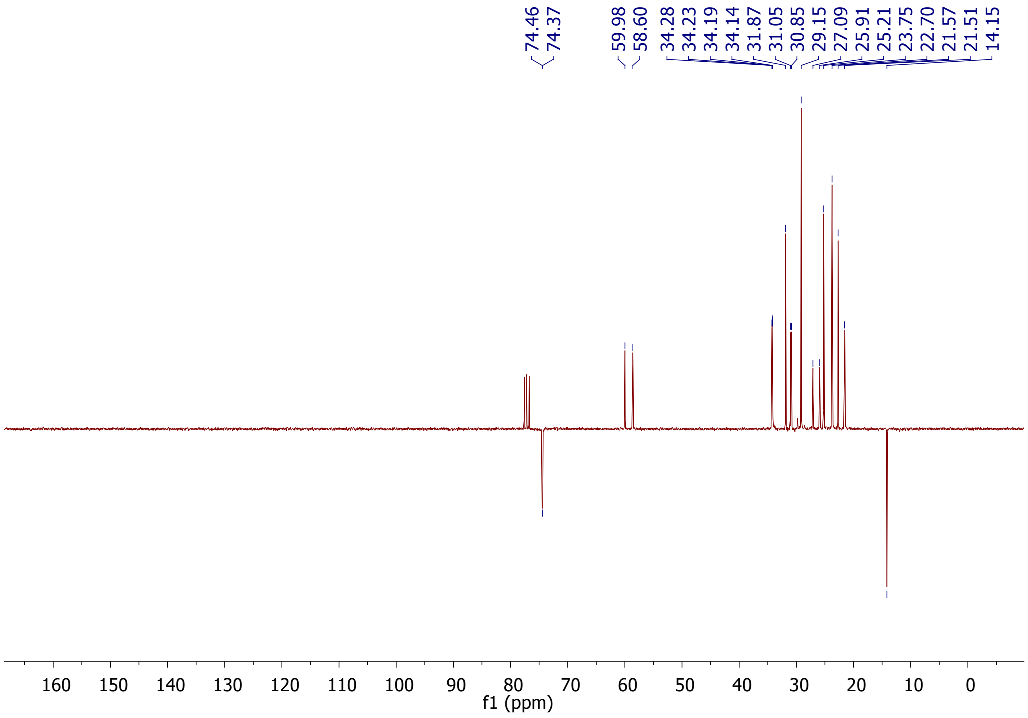

Cyclohexyl phenyl(3-phenylpropyl)phosphinate 3h

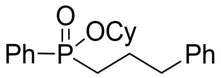

^{31}P-^{1}H decoupled NMR Spectrum ($CDCl_3$, 121 MHz)

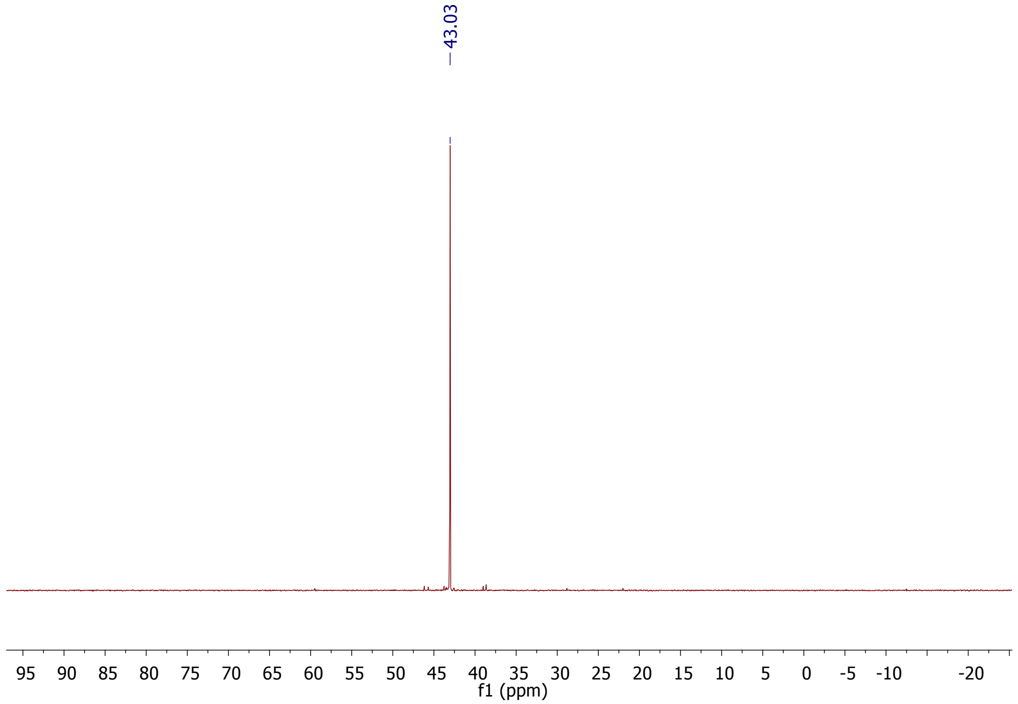

^{31}P-^{1}H coupled NMR Spectrum ($CDCl_3$, 121 MHz)

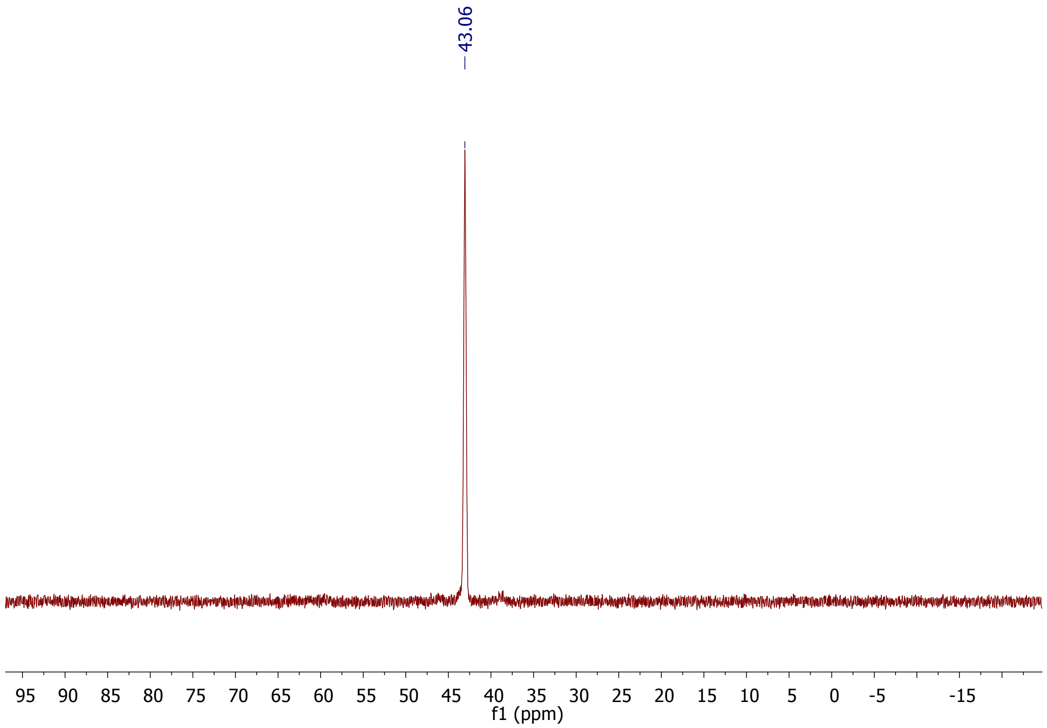

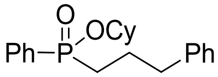

^{1}H NMR Spectrum ($CDCl_3$, 300 MHz)

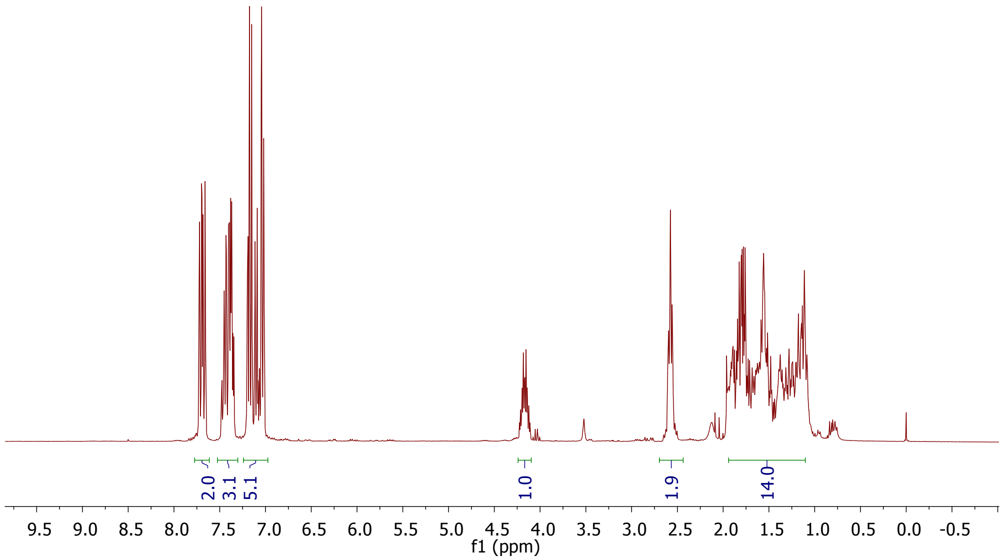

^{13}C NMR Spectrum ($CDCl_3$, 75 MHz)

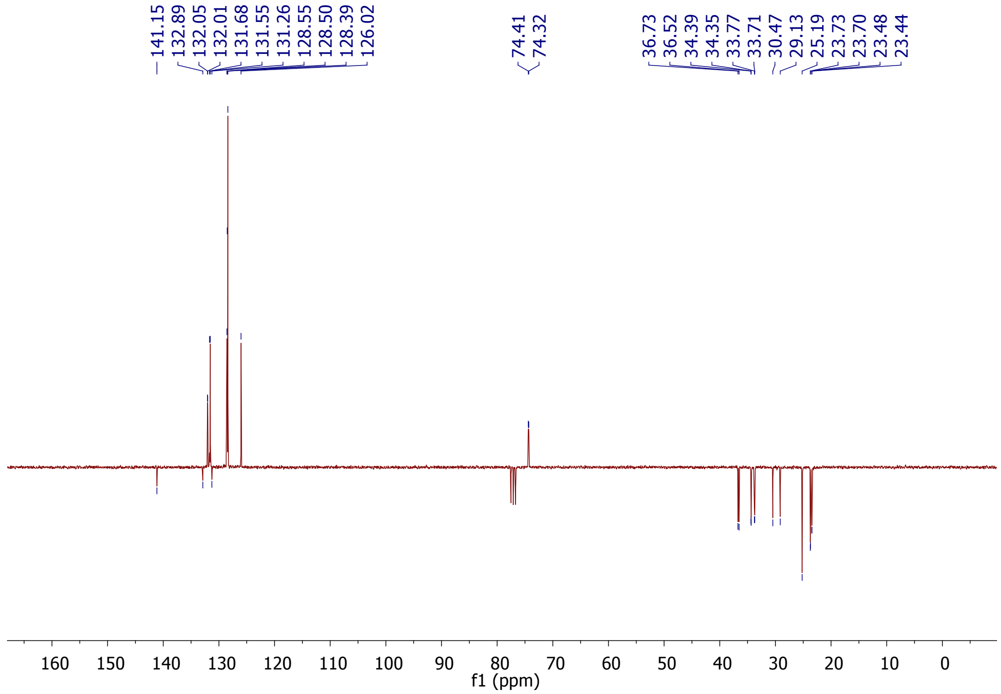

Cyclohexyl (3-methylbutan-2-yl)(phenyl)phosphinate 3i

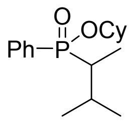

^{31}P-^{1}H decoupled NMR Spectrum ($CDCl_3$, 121 MHz)

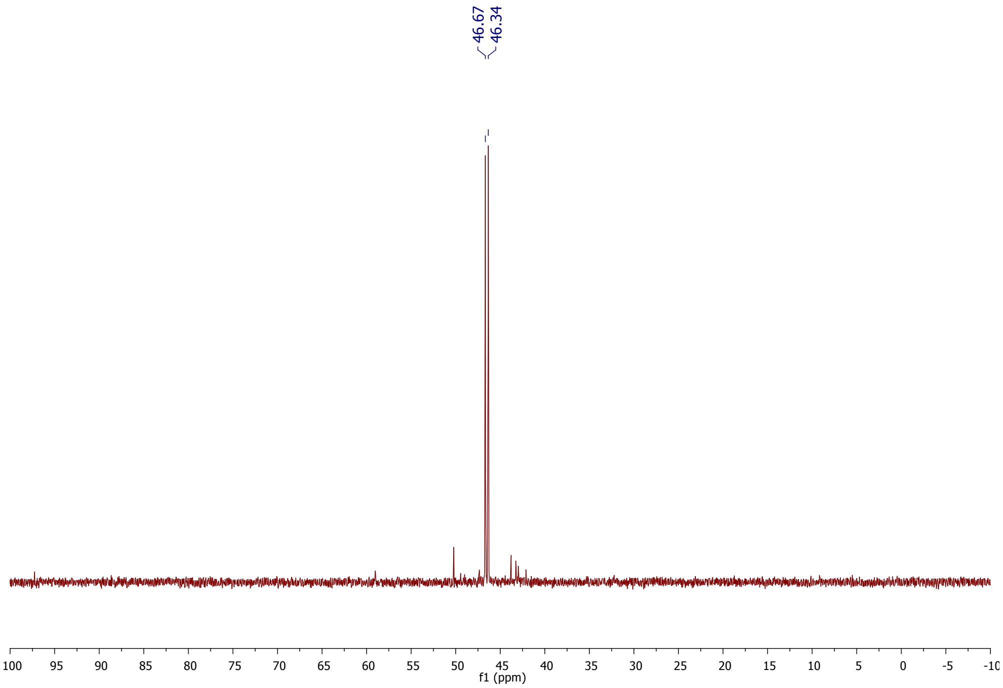

^{31}P-^{1}H coupled NMR Spectrum ($CDCl_3$, 121 MHz)

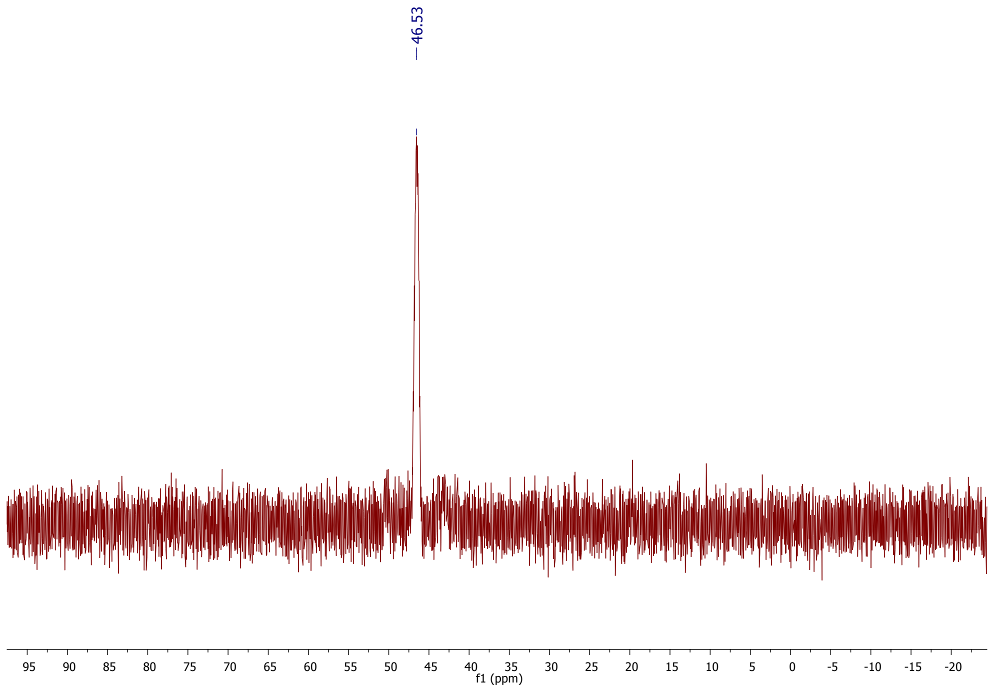

^{1}H NMR Spectrum ($CDCl_3$, 300 MHz)

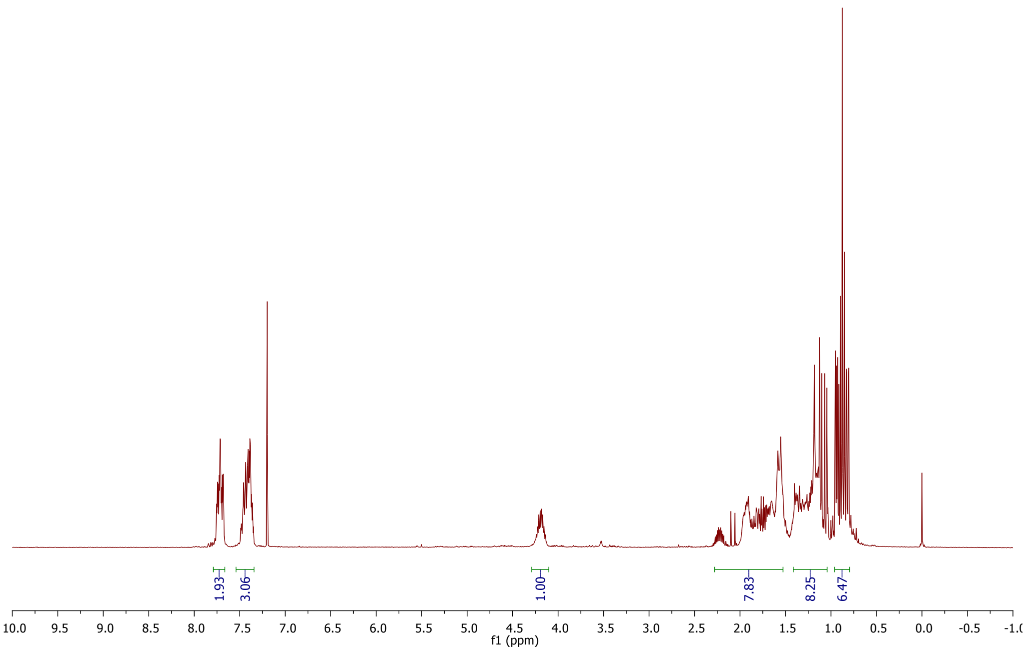

^{13}C NMR Spectrum ($CDCl_3$, 75 MHz)

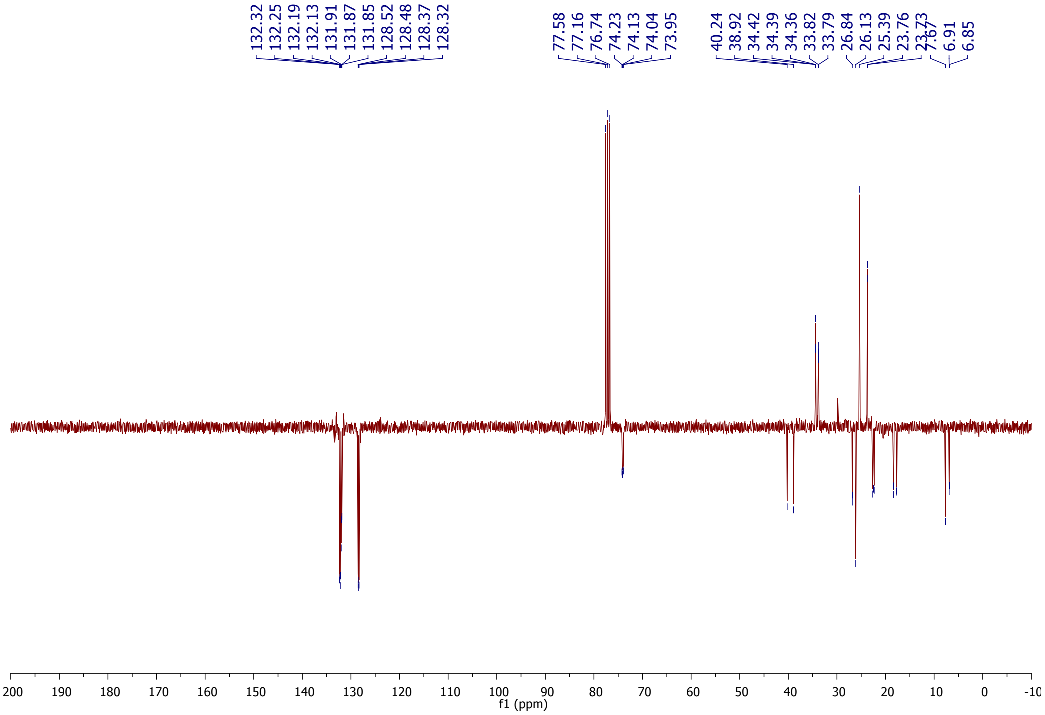

Cyclohexyl (5-bromopentyl)(phenyl)phosphinate 3j

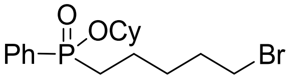

^{31}P-^{1}H decoupled NMR Spectrum ($CDCl_3$, 121 MHz)

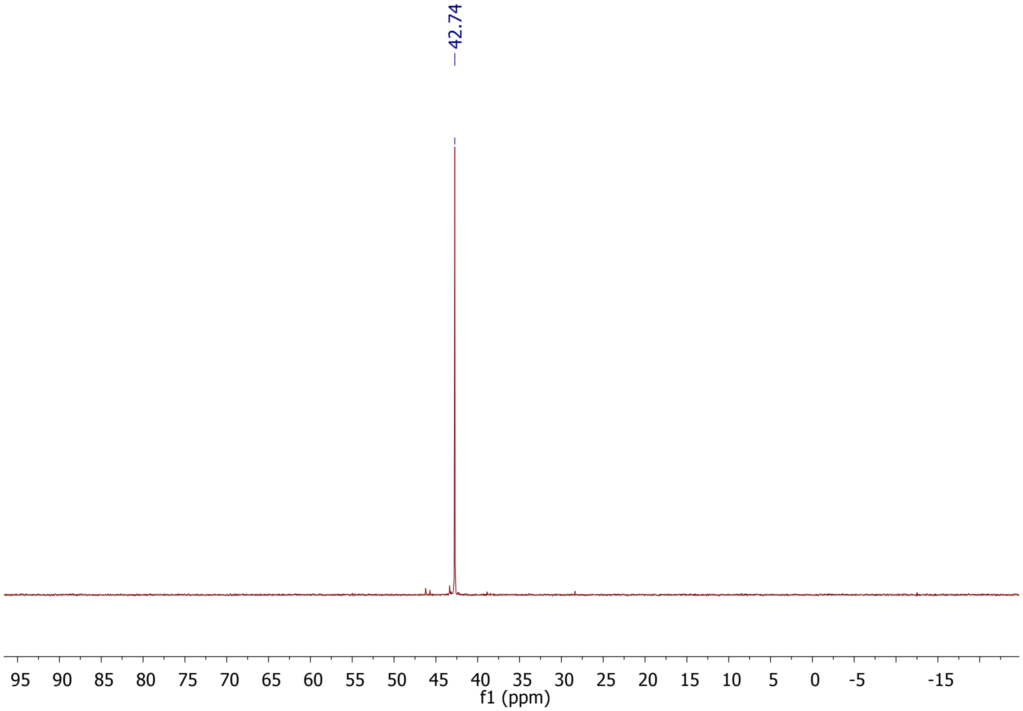

^{31}P-^{1}H coupled NMR Spectrum ($CDCl_3$, 121 MHz)

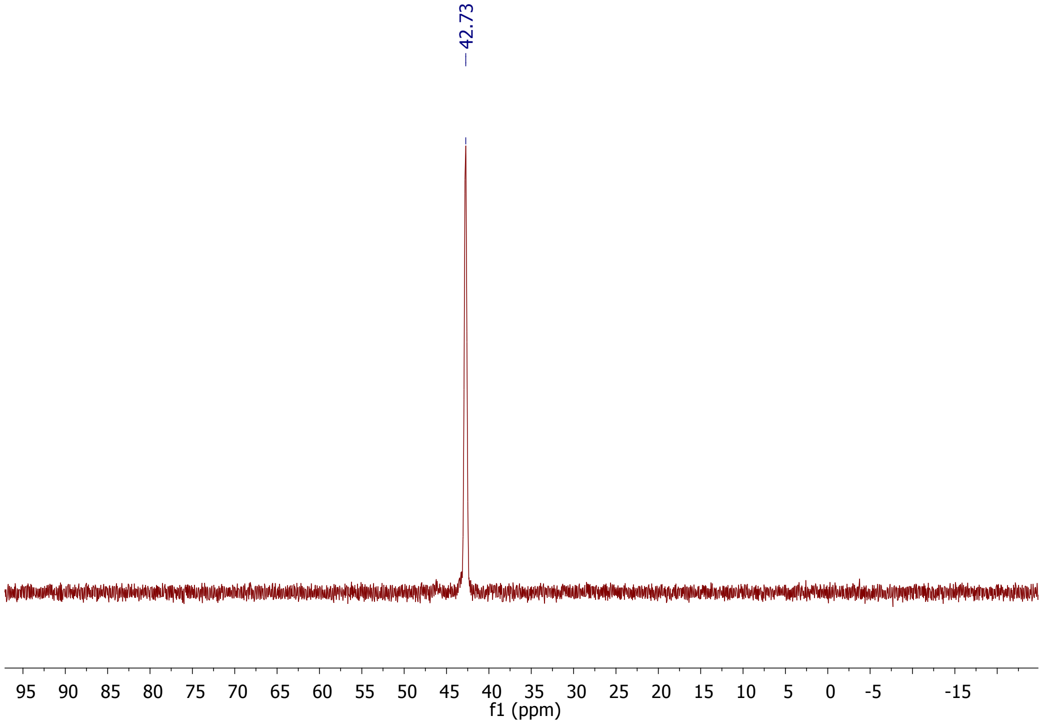

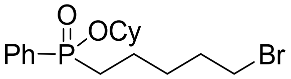

^{1}H NMR Spectrum ($CDCl_3$, 300 MHz)

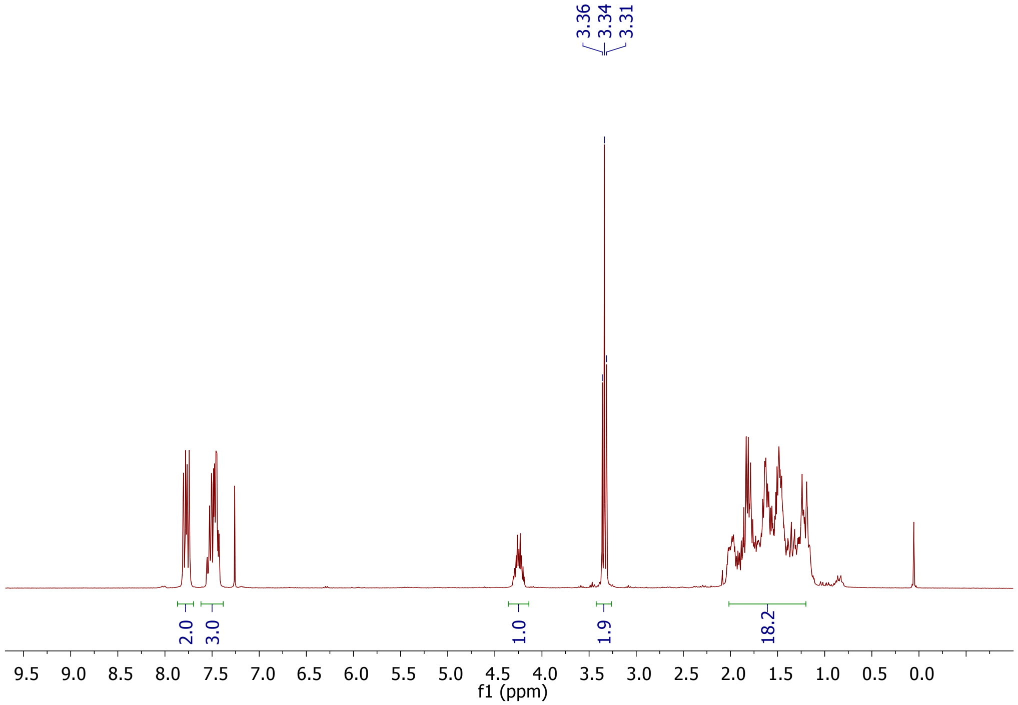

^{13}C NMR Spectrum ($CDCl_3$, 75 MHz)

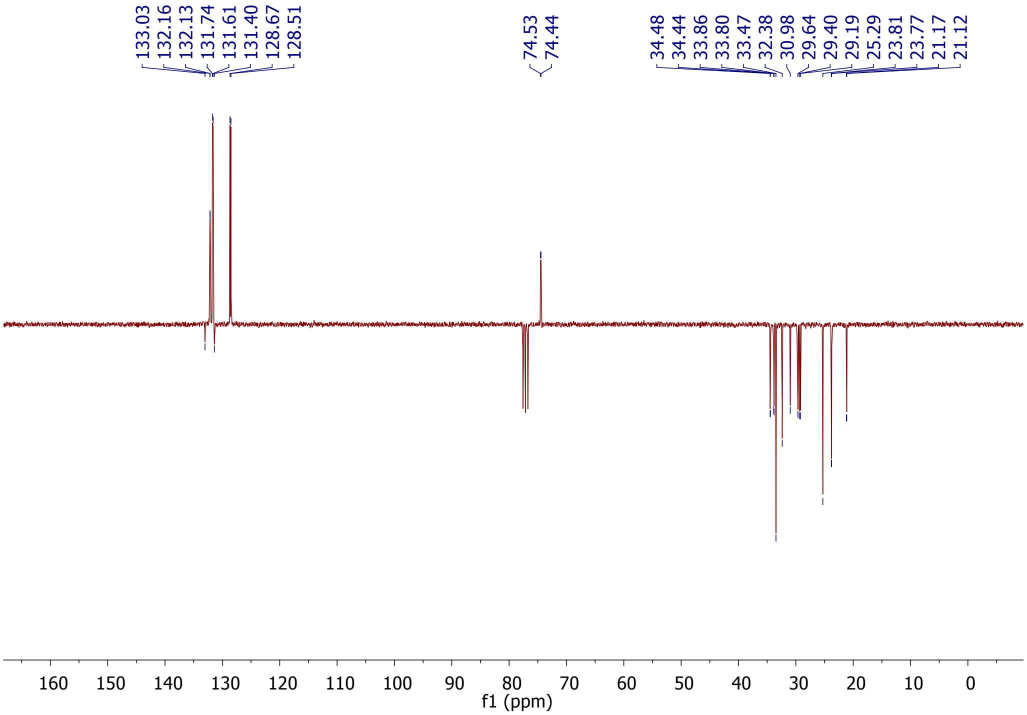

Cyclohexyl phenyl(3-hydroxypropyl)phosphinate 3k

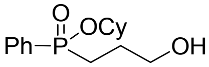

^{31}P-^{1}H decoupled NMR Spectrum ($CDCl_3$, 121 MHz)

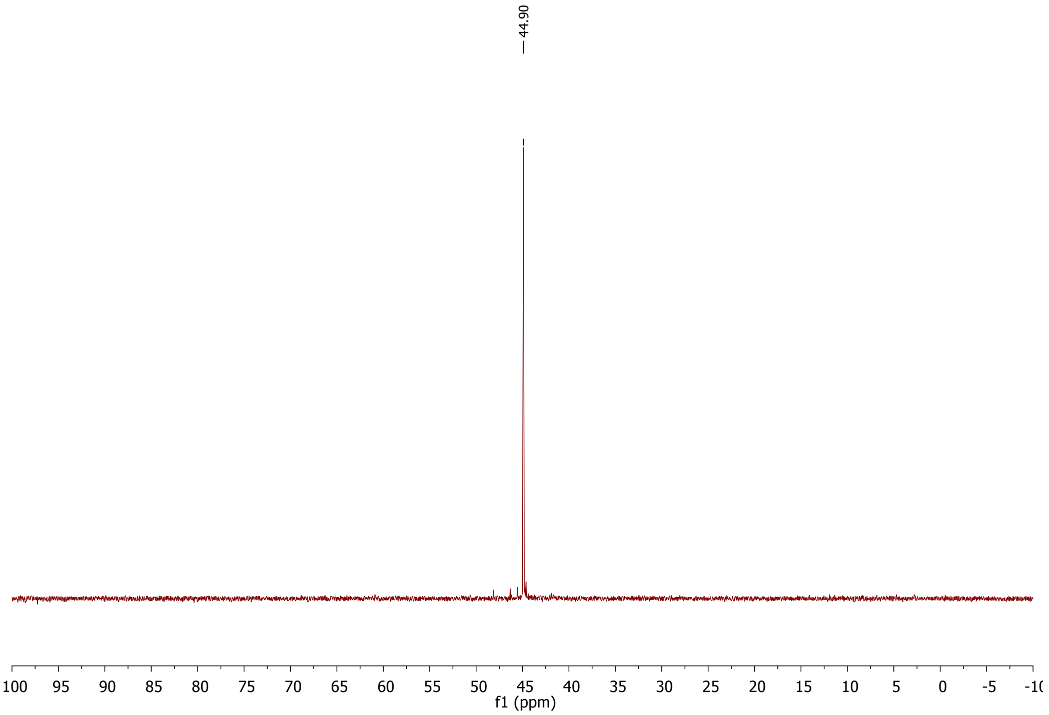

^{31}P-^{1}H coupled NMR Spectrum ($CDCl_3$, 121 MHz)

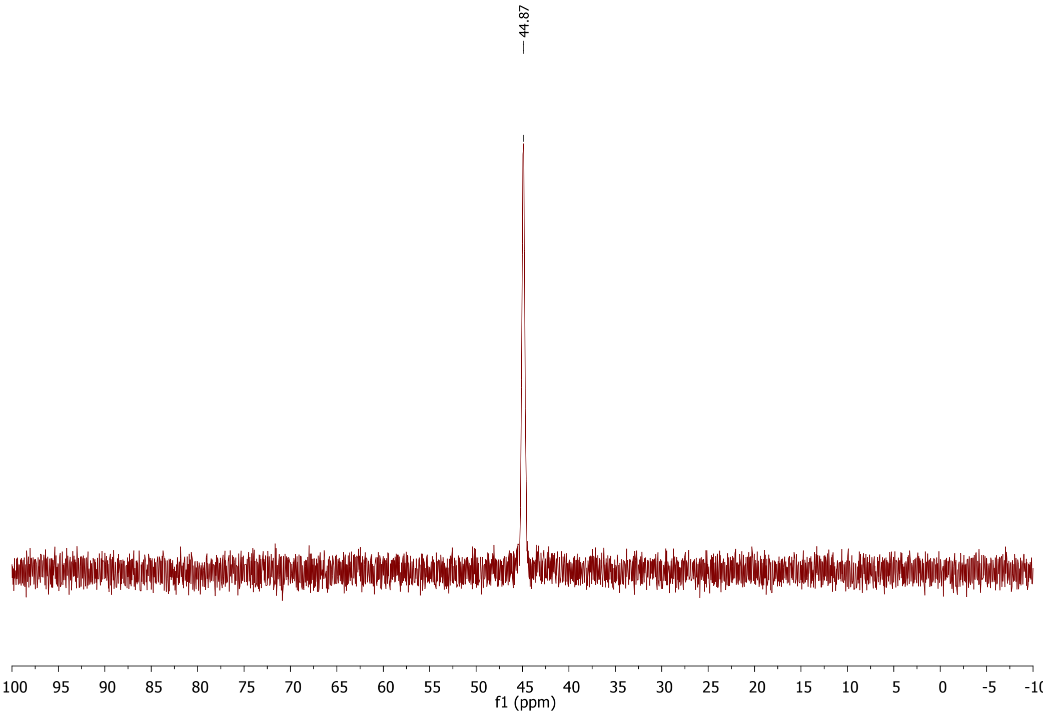

^{1}H NMR Spectrum ($CDCl_3$, 300 MHz)

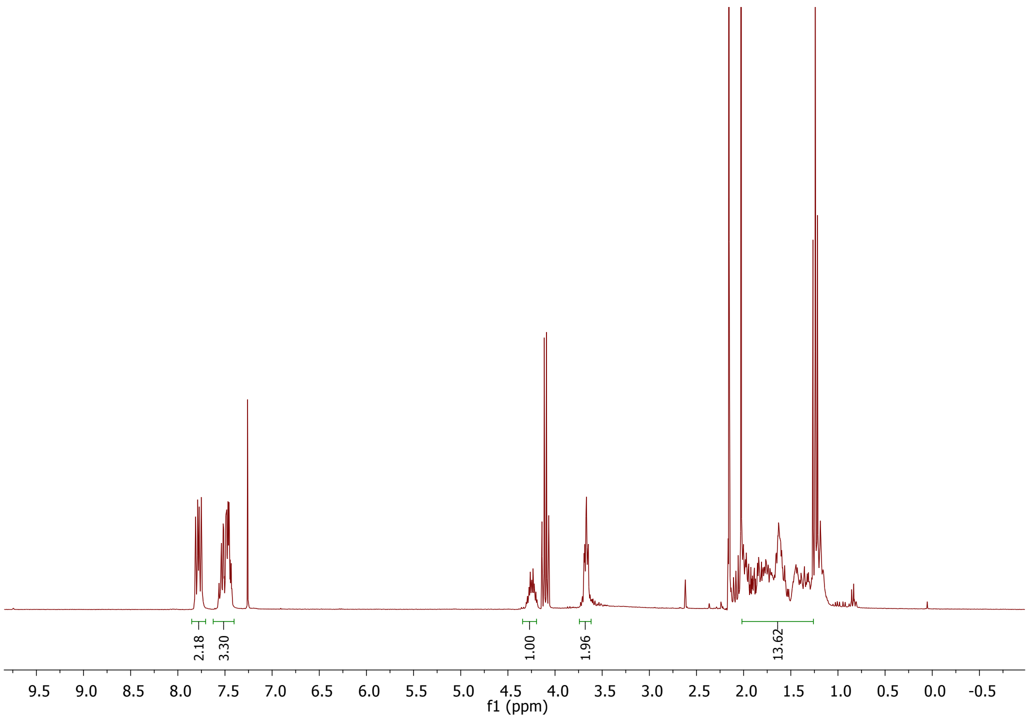

^{13}C NMR Spectrum ($CDCl_3$, 75 MHz)

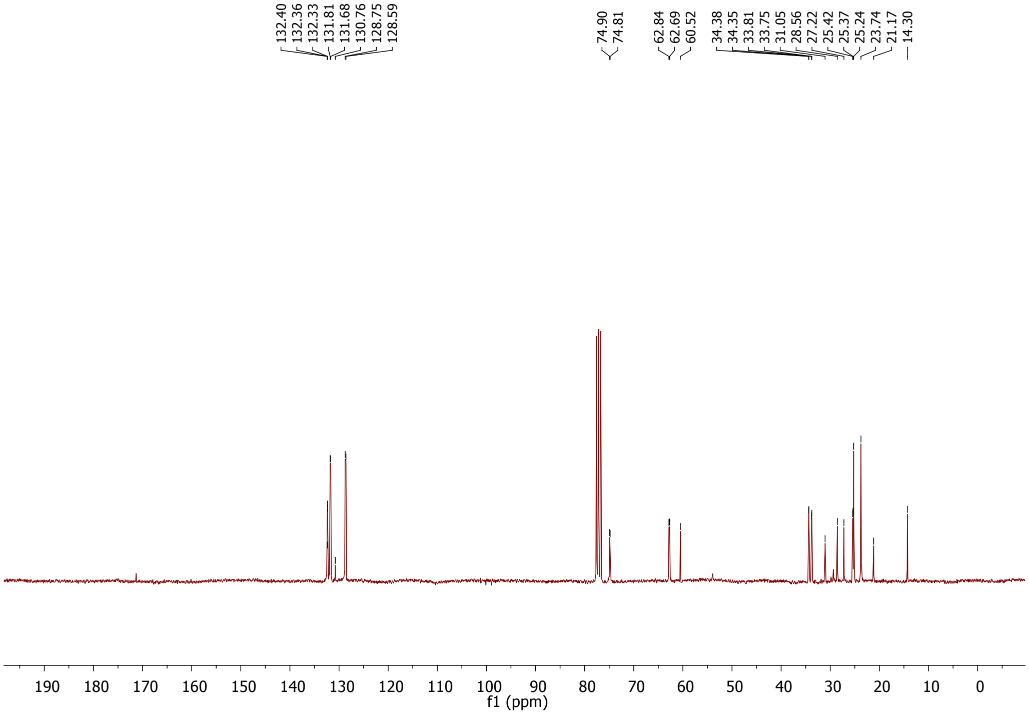

Cyclohexyl (4-hydroxybutyl) (phenyl) phosphinate 3l

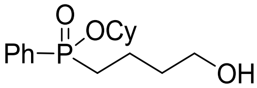

^{31}P-^{1}H decoupled NMR Spectrum ($CDCl_3$, 121 MHz)

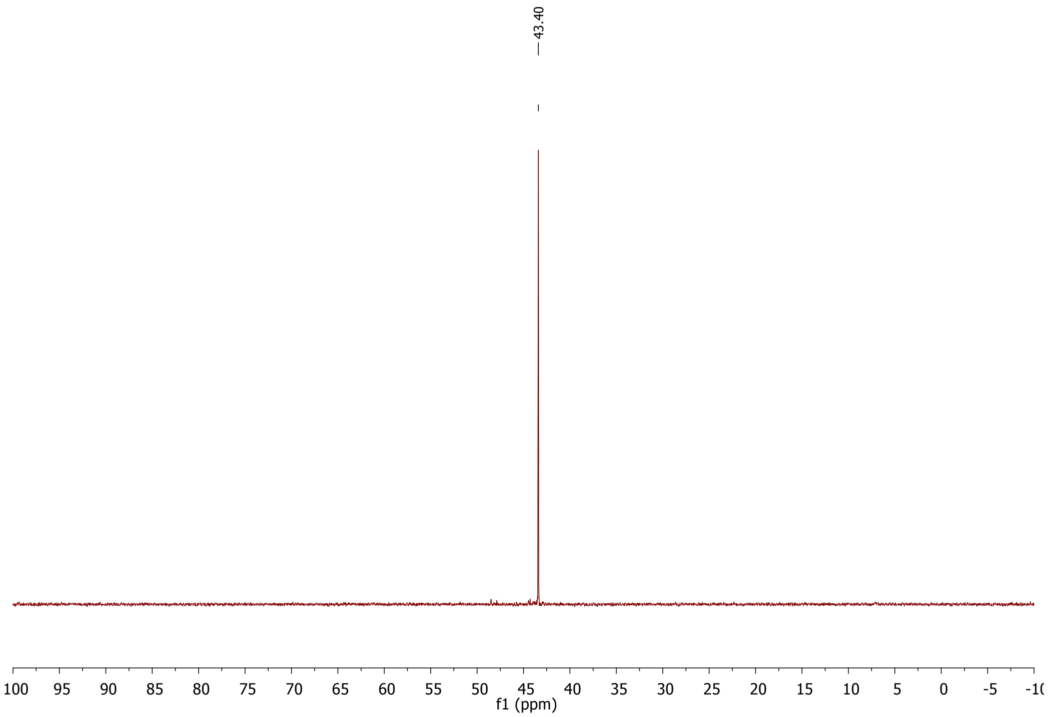

^{31}P-^{1}H coupled NMR Spectrum ($CDCl_3$, 121 MHz)

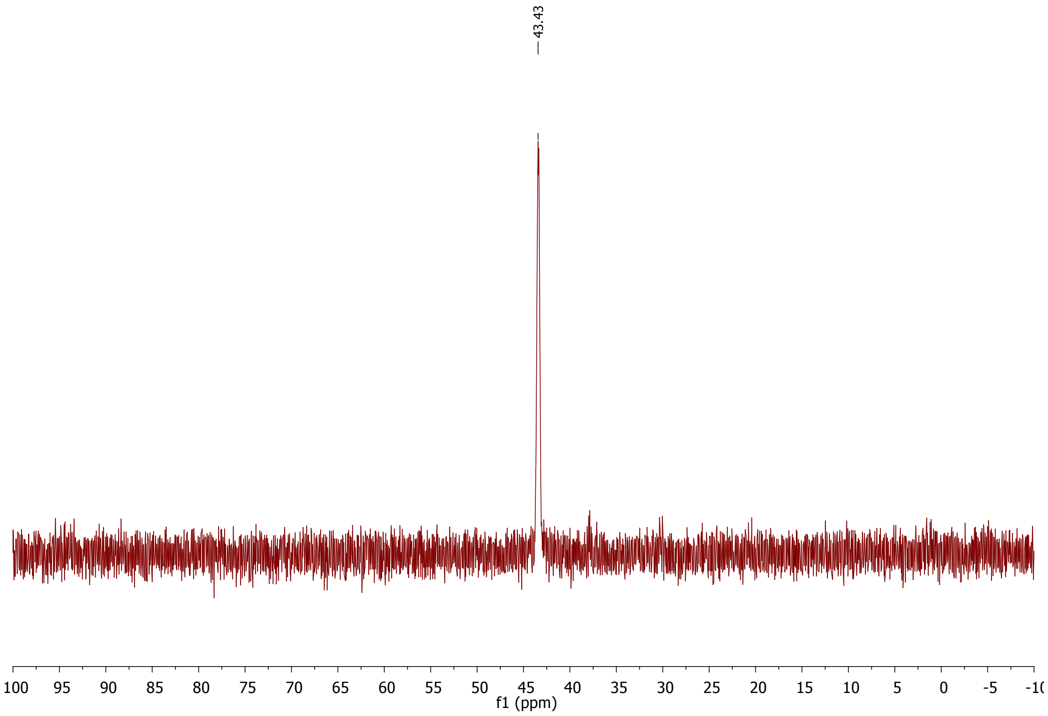

^{1}H NMR Spectrum ($CDCl_3$, 300 MHz)

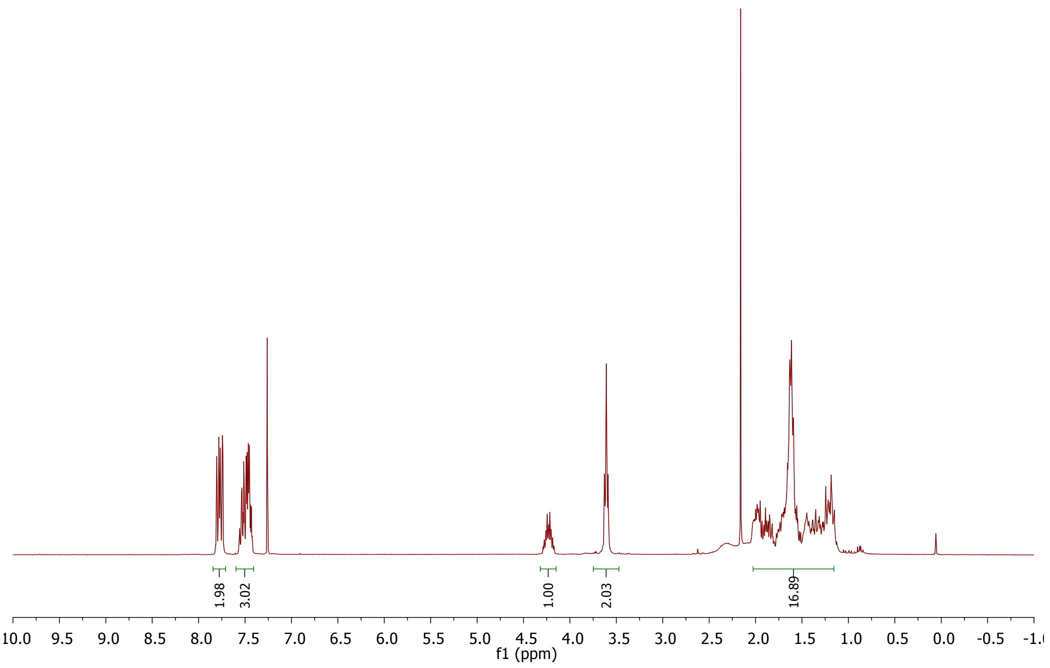

^{13}C NMR Spectrum ($CDCl_3$, 75 MHz)

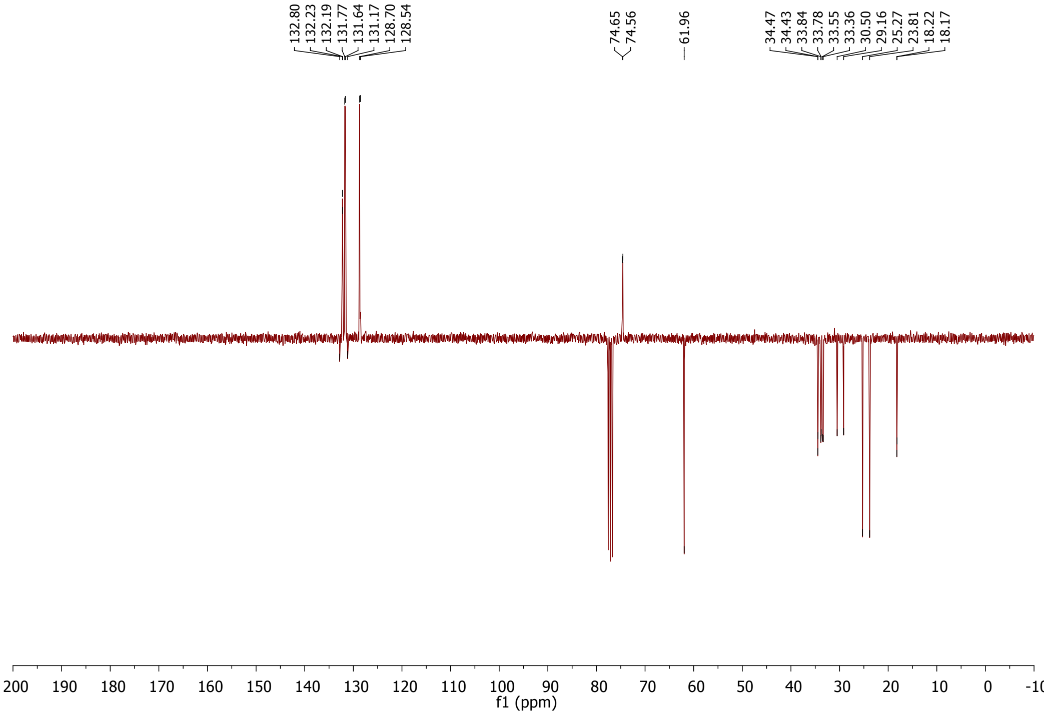

Octyl phosphinic acid 1c

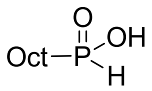

^{31}P-^{1}H decoupled NMR Spectrum ($CDCl_3$, 121 MHz)

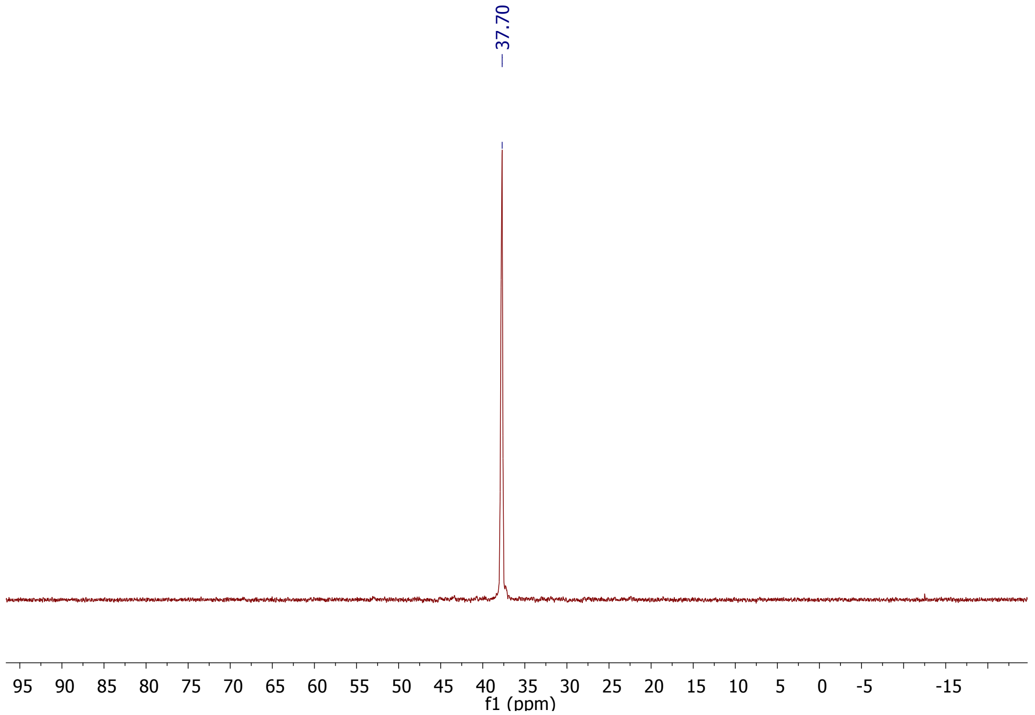

^{31}P-^{1}H coupled NMR Spectrum ($CDCl_3$, 121 MHz)

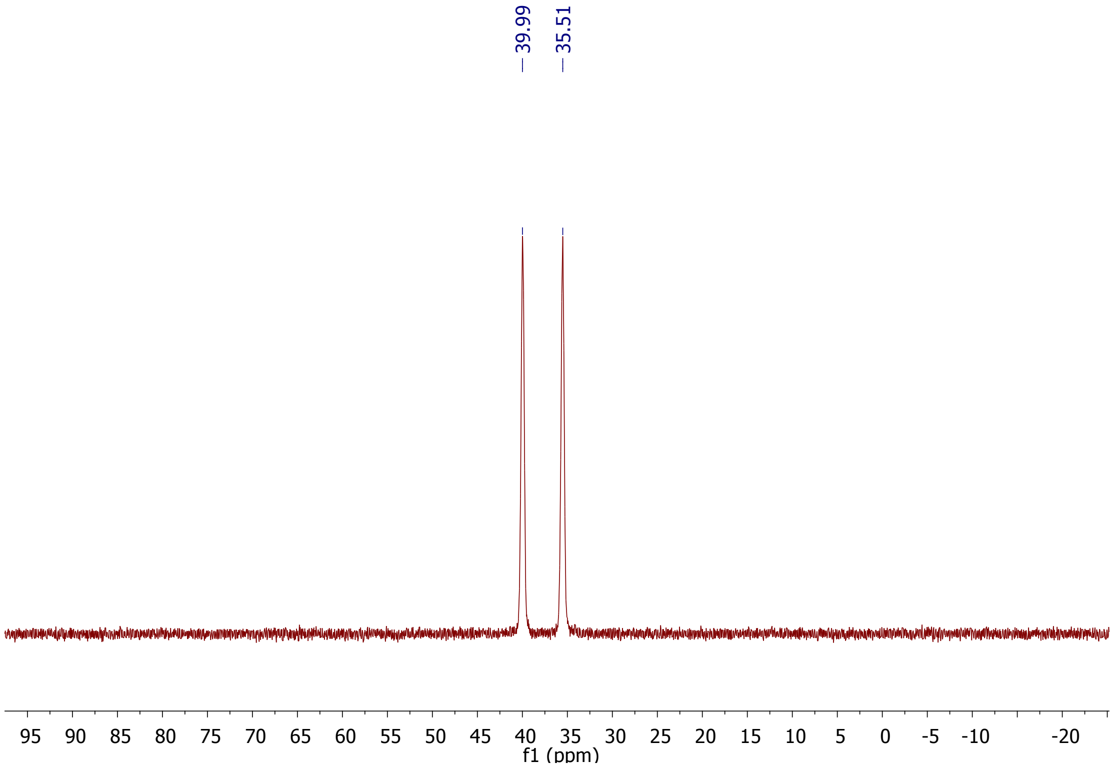

^{1}H NMR Spectrum ($CDCl_3$, 300 MHz)

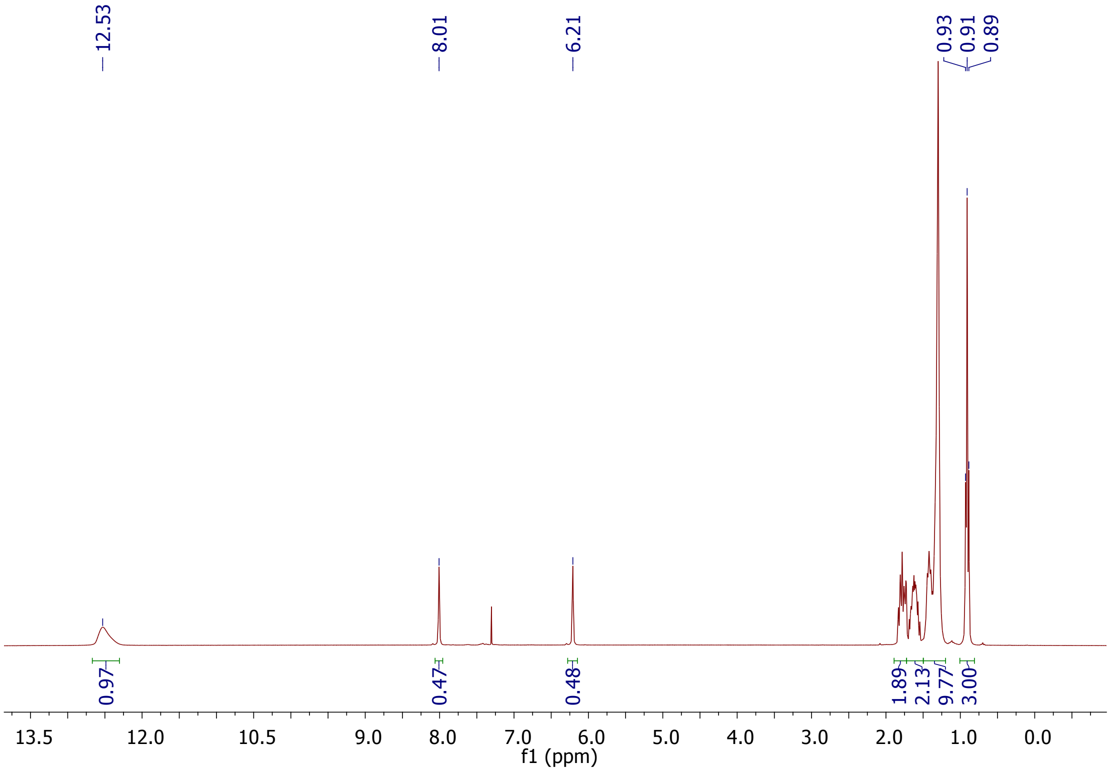

^{13}C NMR Spectrum ($CDCl_3$, 75 MHz)

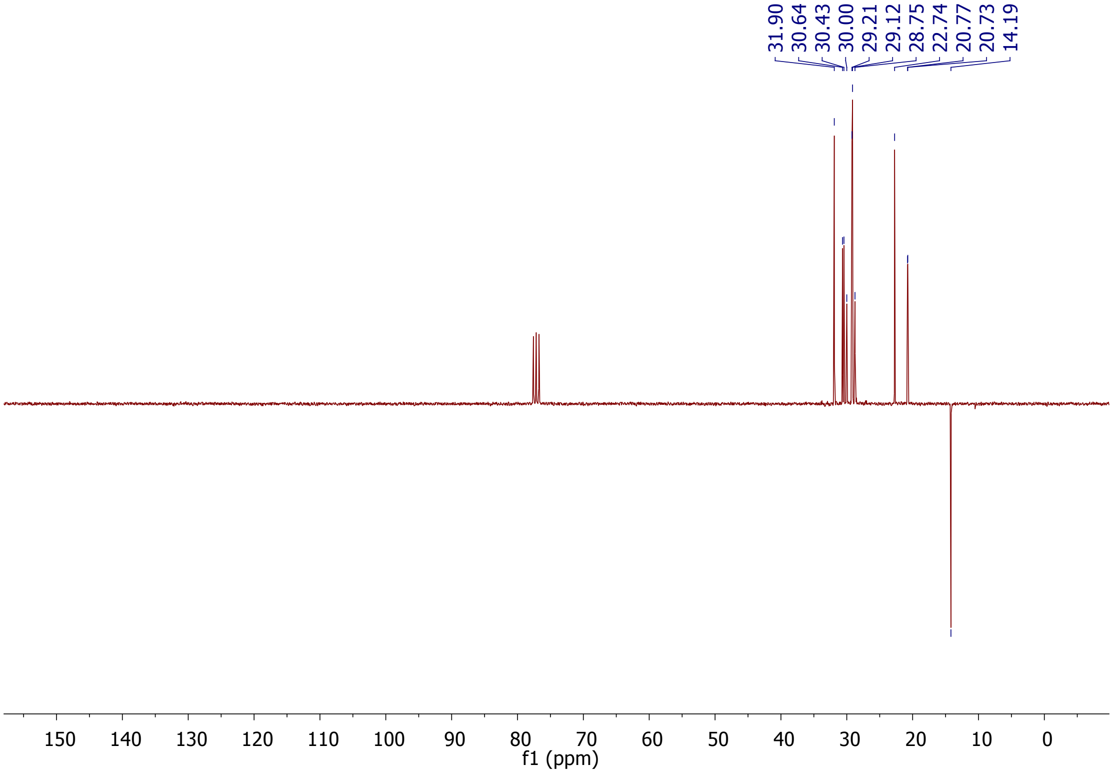

3-Phenylpropyl phosphinic acid 1h

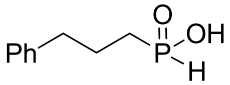

^{31}P-^{1}H decoupled NMR Spectrum ($CDCl_3$, 121 MHz)

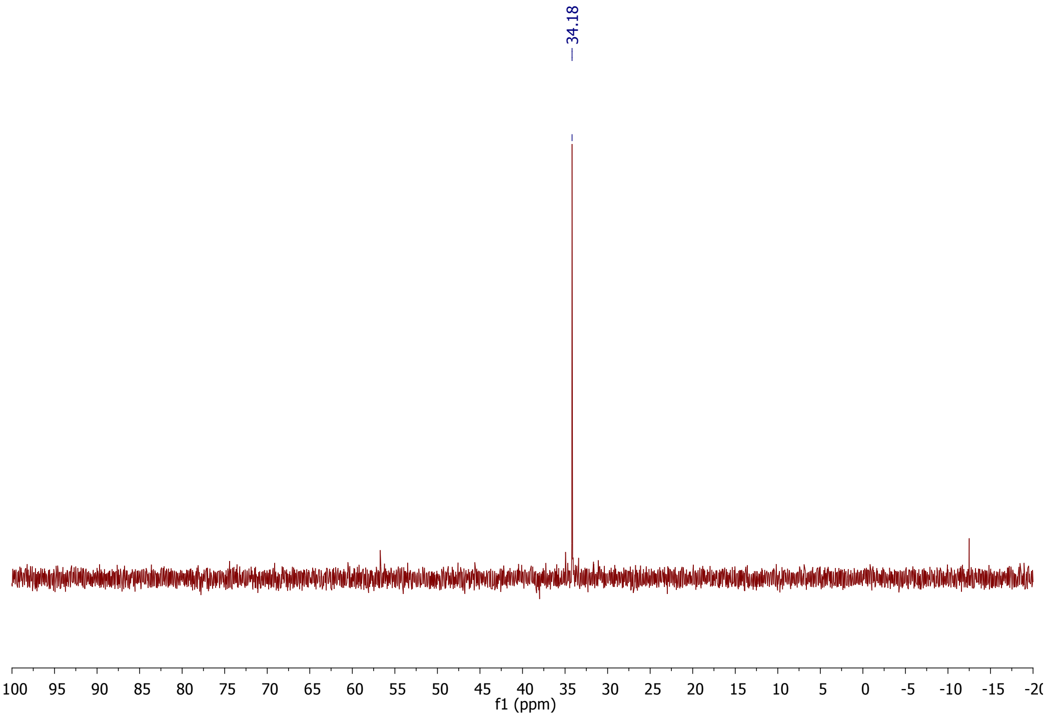

^{31}P-^{1}H coupled NMR Spectrum ($CDCl_3$, 121 MHz)

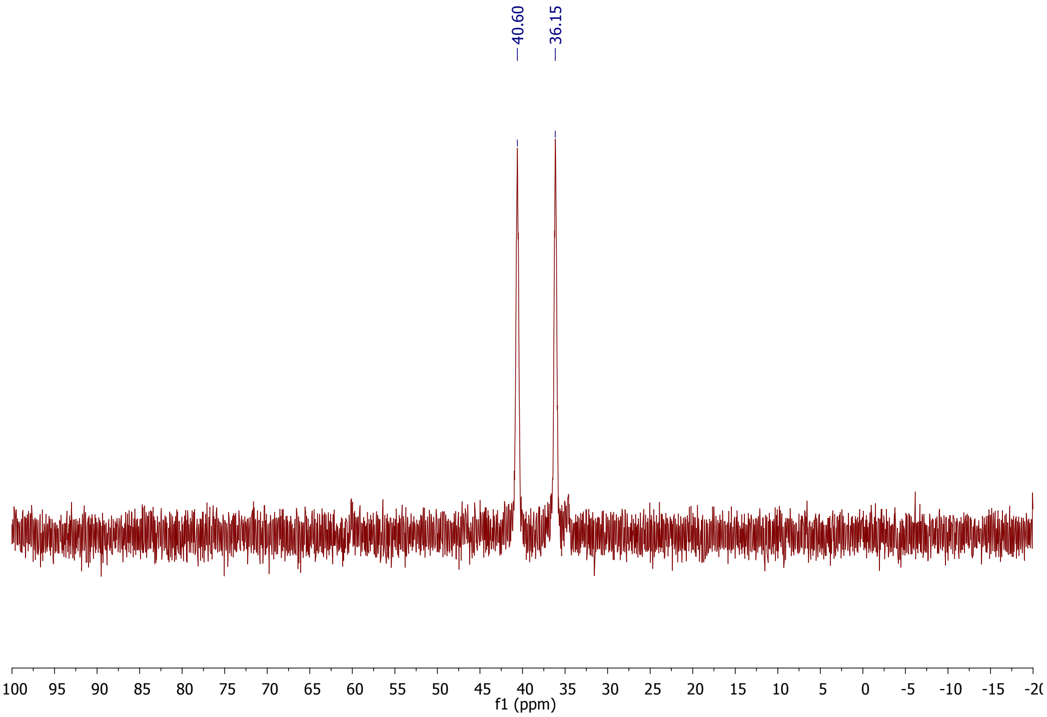

^1H NMR Spectrum ($CDCl_3$, 300 MHz)

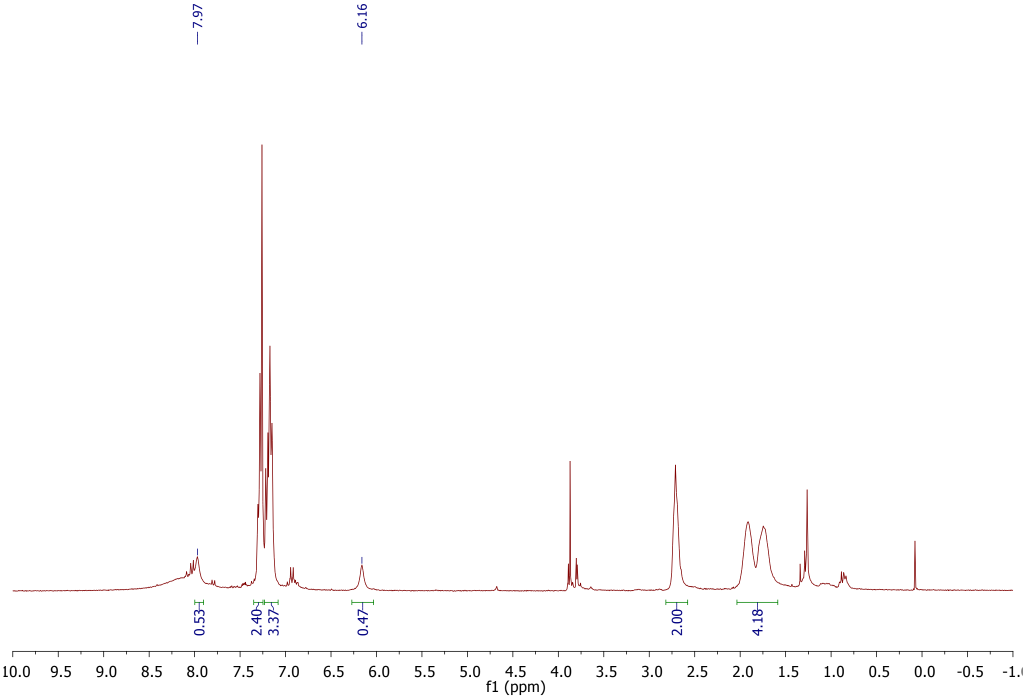

^{13}C NMR Spectrum ($CDCl_3$, 75 MHz)

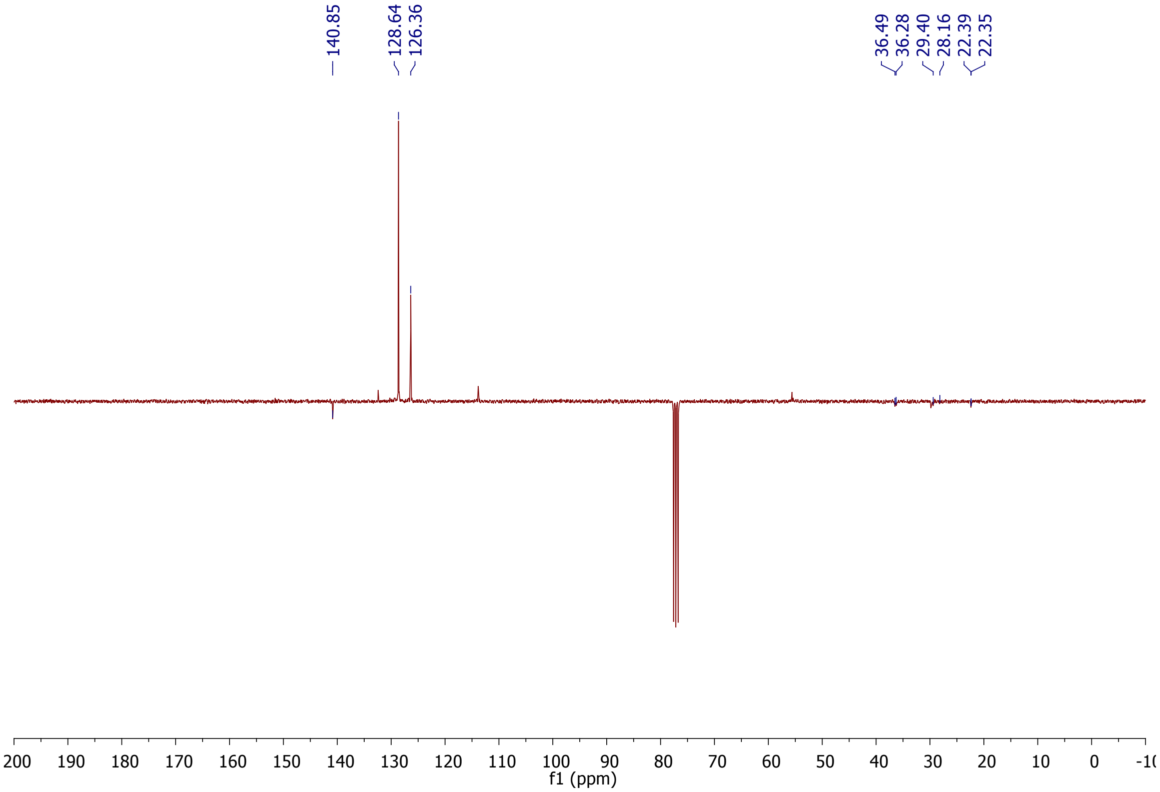

1,2-Dimethylpropyl phosphinic acid 1i

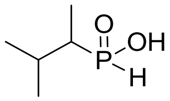

^{31}P-^{1}H decoupled NMR Spectrum ($CDCl_3$, 121 MHz)

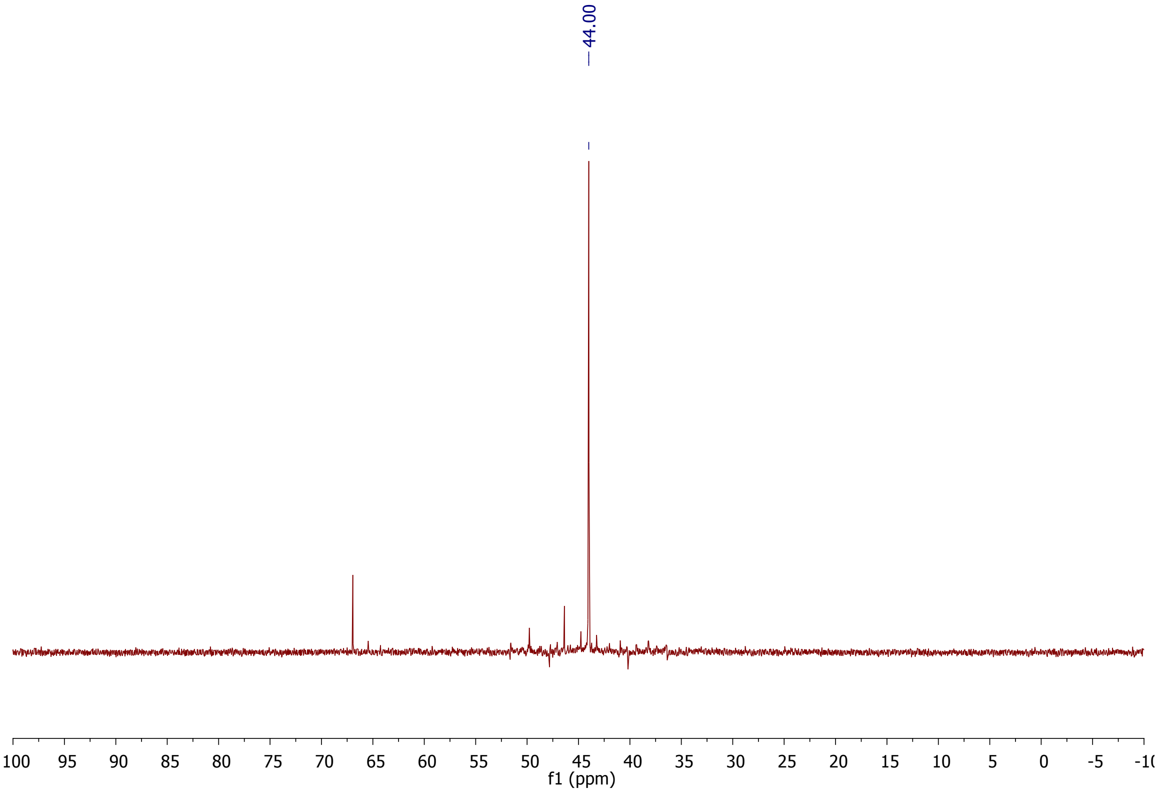

^{31}P-^{1}H coupled NMR Spectrum ($CDCl_3$, 121 MHz)

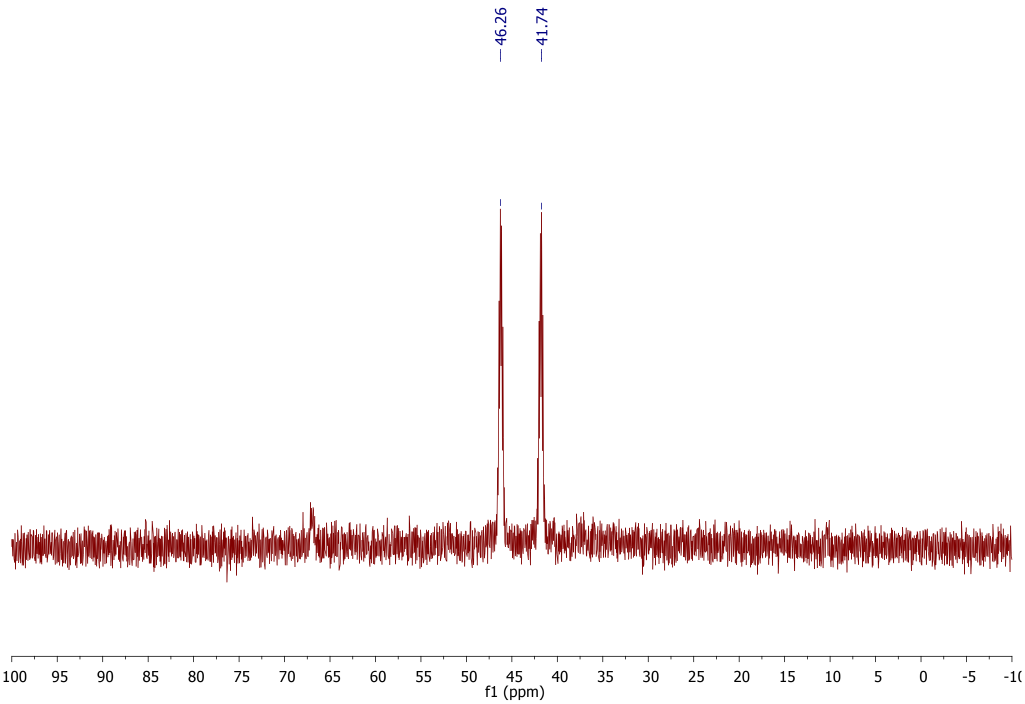

^{1}H NMR Spectrum ($CDCl_3$, 300 MHz)

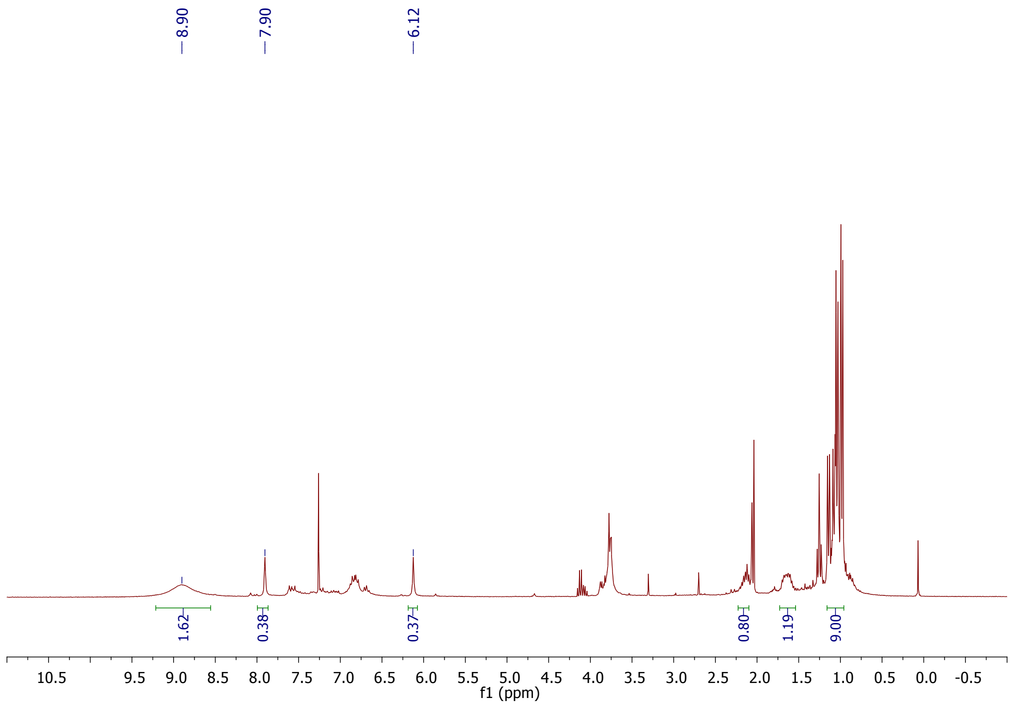

^{13}C NMR Spectrum ($CDCl_3$, 75 MHz)

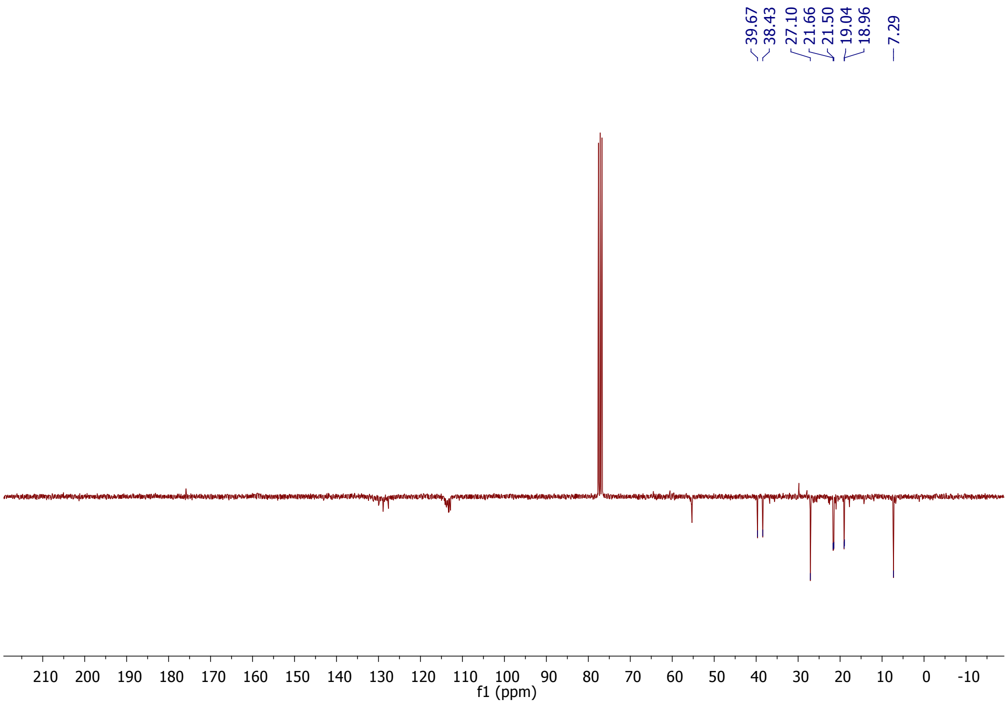

Cyclohexyl phosphinic acid 1k

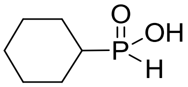

^{31}P-^{1}H decoupled NMR Spectrum ($CDCl_3$, 121 MHz)

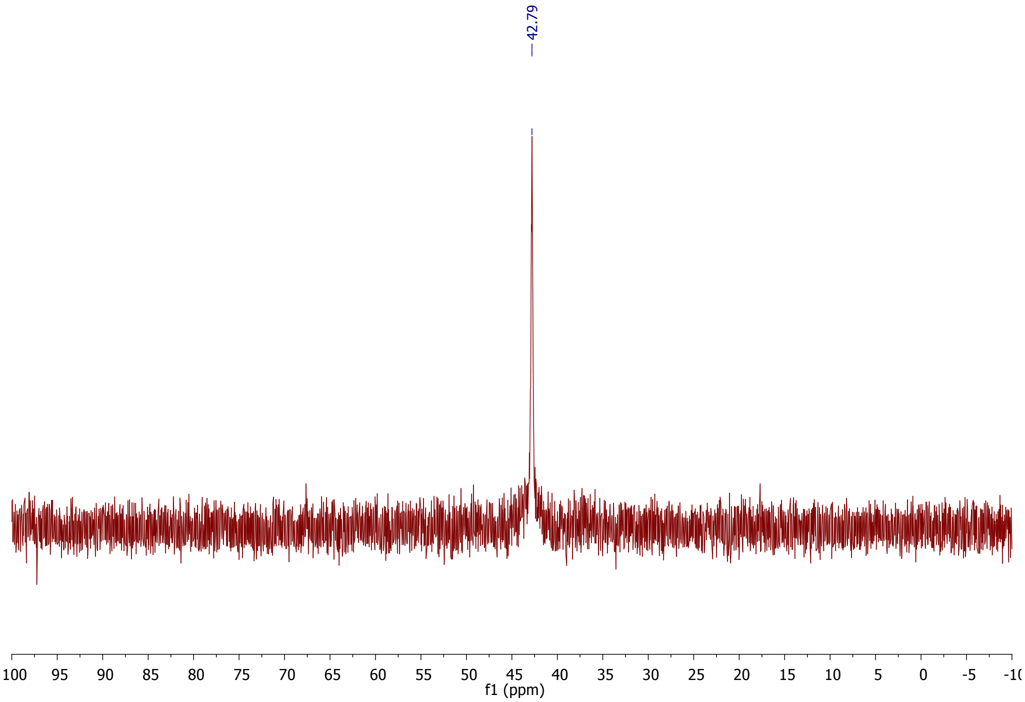

^{31}P-^{1}H coupled NMR Spectrum ($CDCl_3$, 121 MHz)

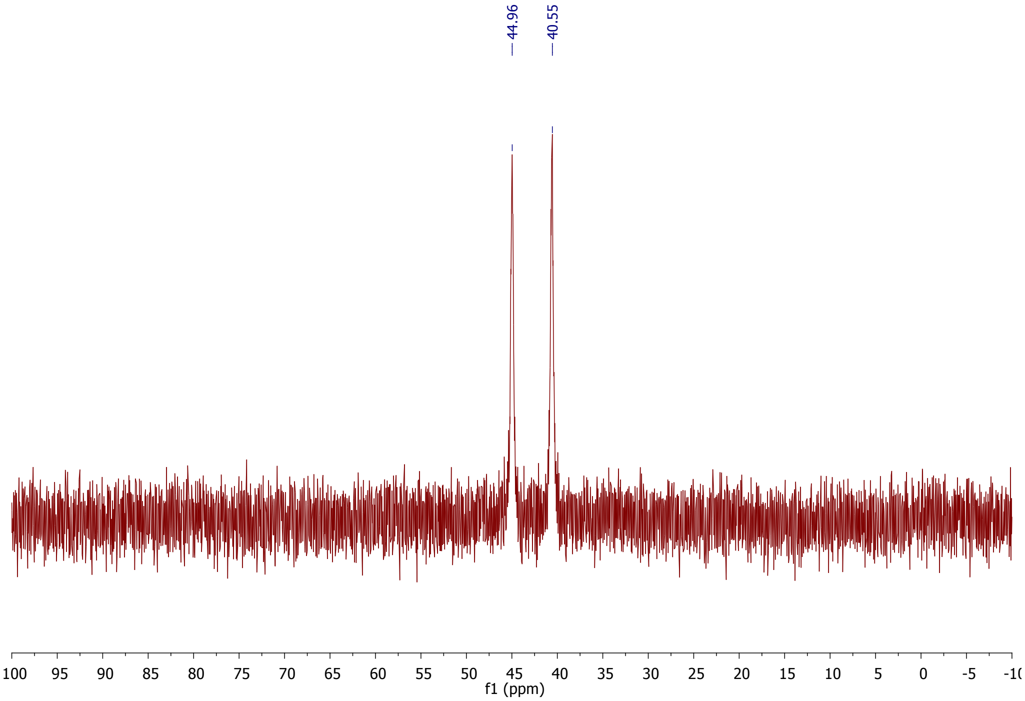

^{1}H NMR Spectrum ($CDCl_3$, 300 MHz)

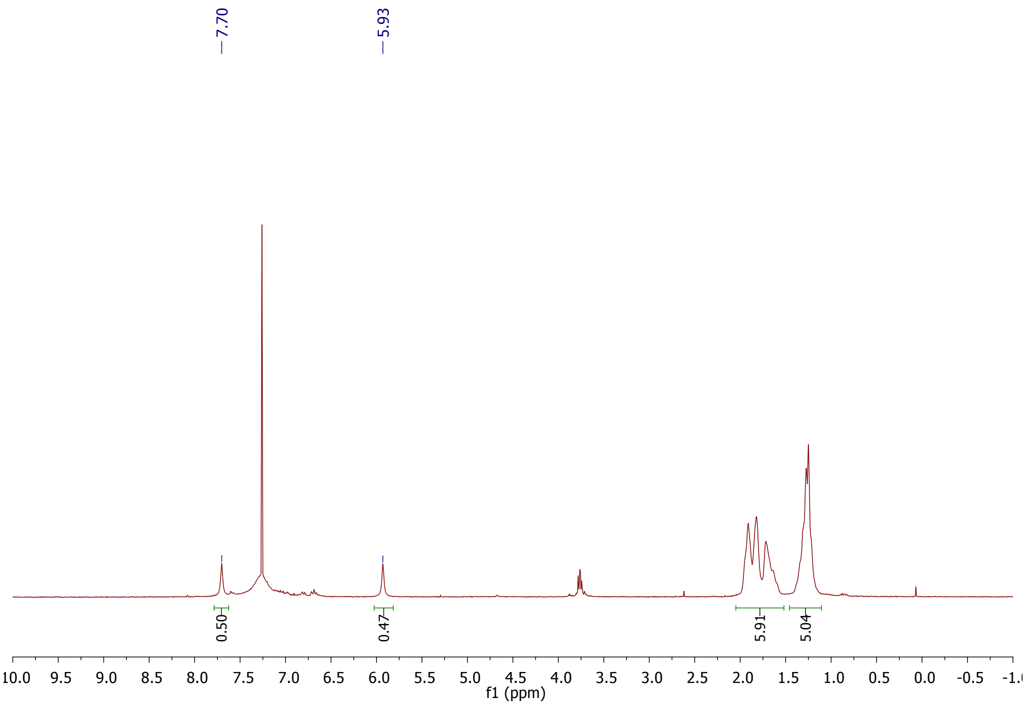

^{13}C NMR Spectrum ($CDCl_3$, 75 MHz)

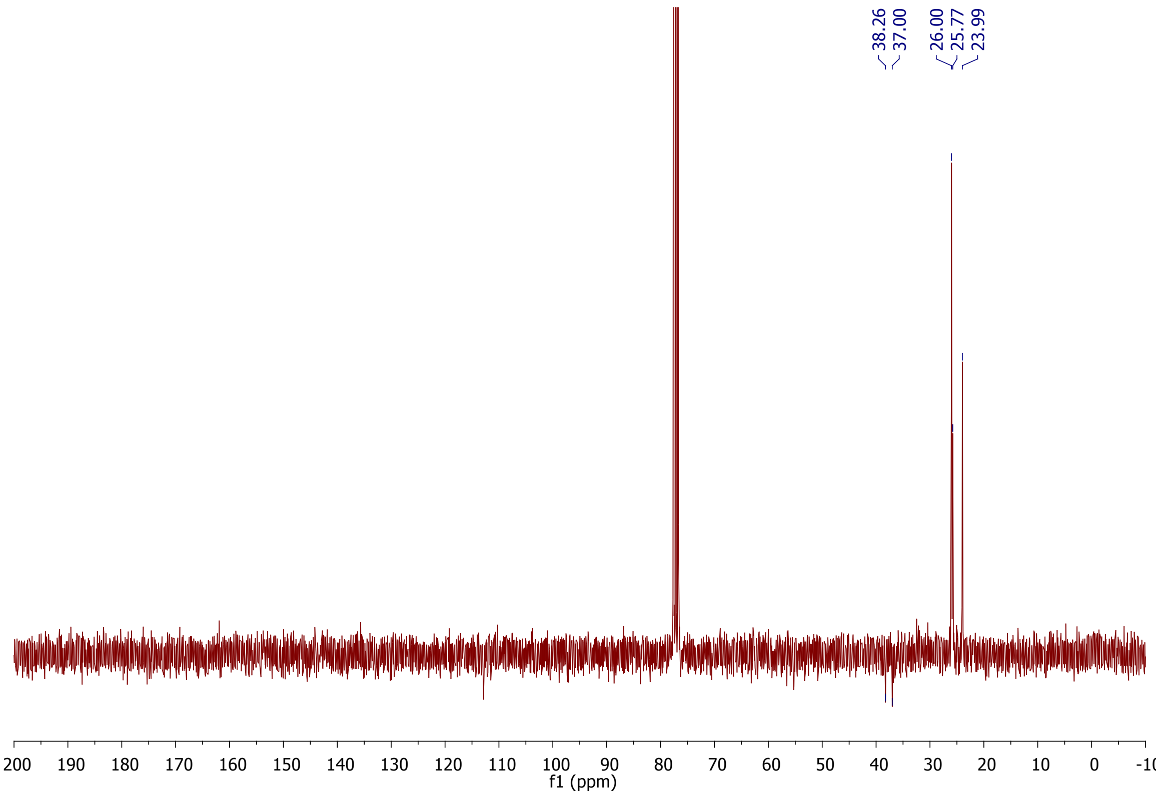

Supplement: RA-008-C7RA12977G-s001 [file RA-008-C7RA12977G-s001.pdf]
